# Paternally multi-generational high-fat diet causes obesity and metabolic disorder through intergenerational DNA methylation

**DOI:** 10.3389/fnut.2025.1680793

**Published:** 2025-10-27

**Authors:** Tianyi Guo, Zuomin Hu, Liyuan Bao, Yunyun Cao, Zhongxing Chu, Mingzhi Wang, Yan Li, Feijun Luo

**Affiliations:** ^1^Shanxi Higher Education Institutions of Science and Technology Innovation Plan Platform, Laboratory of Environmental Factors and Population Health, Key Laboratory of Environmental Pathogenic Mechanisms and Prevention of Chronic Diseases, School of Public Health, Changzhi Medical College, Changzhi, China; ^2^Hunan Key Laboratory of Grain-oil Deep Process and Quality Control, Hunan Key Laboratory of Forestry Edible Resources Safety and Processing, Central South University of Forestry and Technology, Changzhi, China; ^3^Department of Endocrinology, Changzhi Key Laboratory of Precision Medicine for Obesity and Metabolic Diseases, Heping Hospital Affiliated to Changzhi Medical College, Changzhi, China

**Keywords:** high-fat diet, obesity, metabolic disorder, intergenerational inheritance, DNA methylation

## Abstract

**Objective:**

Paternal high-fat diet (HFD) has detrimental effects on offspring. However, the extent of comprehensive damage and the underlying mechanisms associated with sustained multigenerational HFD exposure remain unclear. This study aims to investigate intergenerational progressive accumulation of obesity and glycolipid metabolic disorders, as well as mechanisms mediated by DNA methylation.

**Methods:**

We performed a novel paternally multi-generational HFD consumption model in male C57BL/6 J mice, while excluding maternal gestational effects and any confounding influences from females. The body weight and glycolipid metabolism indicators of each generation of male mice were determined. The intergenerational transmission of CpG methylation and gene expression variation was detected through mRNA microarray, methylated DNA immunoprecipitation (MeDIP)-chip, bisulfite sequencing, RT-qPCR, and Western blot etc. Analysis, to indicate genes involved in glycolipid metabolism related to the intergenerational reprogramming.

**Results:**

The HFD caused intergenerational accumulation of body weight increase, disturbance of glycolipid metabolism, and insulin insensitivity in male offspring. MeDIP/gene-chip results indicated that paternal HFD significantly modified gene expression and DNA methylation profiles in the liver and sperm of offspring. The majority of differential genes exhibited hypermethylation in promoter regions and reduced expression in the liver, which were linked to the glucolipid metabolic signaling pathway. The elevated promoter methylation and expression states of *Spns2*, *Lonp1,* and *Hk1*, which are involved in glycolipid metabolism, were inherited by offspring.

**Conclusion:**

This research shows that paternal sustained multi-generational HFD could induce intergenerational progressive accumulation of obesity and metabolic disorder through DNA methylation regulation, and identifies the target genes related to the intergenerational reprogramming, which provides new insights for the establishment of healthy diets and lifestyles.

## 1 Introduction

The global prevalence of obesity, a multifaceted metabolic disorder, constitutes a persistent health crisis in the 21st century, with nearly half of the adult population worldwide classified as overweight or obese, significantly impacting public health and healthcare systems (1). Obesity is also associated with various metabolic disorders, including dysglycemia, dyslipidemia, steatosis, insulin resistance, and chronic inflammation. These conditions significantly increase the risk of developing type 2 diabetes mellitus (T2DM) and cardiovascular diseases (CVD). Among environmental risk factors, the Western-style high-fat diet (HFD) is a major contributor to obesity and metabolic syndrome (2). The consumption of high-fat, high-energy foods disrupts glycolipid metabolism and leads to abnormal lipid accumulation. Notably, children of obese parents are at an increased risk of developing obesity and metabolic diseases (3). Some evidence suggests that the parental dietary patterns and nutritional status can influence the metabolic phenotypes of their offspring. For instance, the children born in the Dutch Famine in 1944–1945 developed an increased risk of T2DM, cardiovascular diseases, metabolic disorders, and other cognitive dysfunctions compared with control counterparts (4, 5). The evidence from the Overkalix cohort study conducted in a remote Swedish town demonstrated that restrictive access to nutritional foods was associated with mortality rates two generations later. In a gender, paternal grandfather’s food supply was associated with metabolism in grandsons, whereas paternal grandmothers’ food supply was linked to outcomes in their granddaughters (6). These diet-induced alterations in the offspring’s metabolic phenotypes may be mediated by non-genetic factors (7).

Epigenetic mechanisms, which correspond to the concept of genetics, encompass DNA methylation, histone modification, chromatin remodeling, and non-coding RNA-mediated changes in gene expression without DNA sequence alteration, and provide a potential scientific explanation for the intergenerational inheritance of disease phenotypes (8). Parental exposure to environmental factors (e.g., chemical substances, diet) alters epigenetic marks (9). If these modifications persist in the gametes and escape reprogramming during gametogenesis and fertilization, the resulting detrimental effects can be transmitted to subsequent generations (10). The DNA methylation, referring to the addition of a methyl group to the fifth carbon of cytosine within CpG dinucleotides in gene promoter regions, regulates gene expression. This process is extensively implicated in obesity and glycolipid metabolism (11). Particularly, the obesity or HFD consumption could modulate the DNA methylation of various genes involved in metabolic imbalance (12–15). Certain methylation states may be maintained, leading offspring to exhibit parental-like gene expression patterns (16), accompanied by excess weight and metabolic disorders. For example, maternal HFD exposure increased body size and reduced insulin sensitivity intergenerationally, implicating heritable epigenetic reprogramming of metabolism-associated genes (17, 18). However, it is difficult to distinguish the maternal environment’s influence on epigenetics from direct *in utero* exposure effects. To eliminate confounding gestational effects, investigating the paternal lineage is crucial, as fathers contribute primarily spermatozoa. Paternal dietary patterns (19) or disease (20) disrupt offspring glucolipid metabolism. However, most studies report metabolic phenotypes transmission within two generations (15), often without observing a consistent offspring obese phenotype (increased body weight). Crucially, key unresolved questions include: How many consecutive generations of HFD induction are required to induce significant, stable metabolic abnormalities, particularly obesity in offspring, and the mechanistic basis for persistent alterations in DNA methylation and gene expression patterns remains poorly defined.

In this research, a uniquely novel paternally multi-generational HFD consumption model was performed in C57BL/6 J mice, to investigate the cumulative impact of consecutive HFD exposure on offspring. Male mice were maintained on HFD across generations and bred with normal-diet females. Male offspring of each generation from pairings were selected as subjects until significant weight gain and stable phenotypic changes were observed in offspring. Genome-wide and promoter-specific DNA methylation changes in glycolipid metabolism genes, alongside corresponding expression shifts, revealed underlying molecular mechanisms. These results would elucidate the mechanistic basis for the intergenerational inheritance of increased risk of obesity and metabolic disorders induced by HFD. Furthermore, they provide an important scientific theoretical basis and guidance to inform healthy diet and lifestyle establishment, which have profound implications for human and our progeny’s health.

## 2 Materials and methods

### 2.1 Animal experiment

The C57BL/6 J mice (male, 8 weeks old) were obtained from Hunan SJA Laboratory Animal Co., Ltd. (SLAC, Changsha, Hunan, China). The animal care and experimental protocols were in accordance with the requirements of the Guidelines for the Care and Use of Experimental Animals, and were approved by the Hunan Laboratory Animal Center (Hunan Drug Safety Evaluation Research Center Co., Ltd.; HNSE2021(5)068, Liuyang, Changsha, Hunan, China) and the office of Animal Experiment Ethics at the Central South University of Forestry and Technology. The temperature, humidity, and light–dark cycle were maintained at 25 ± 2 °C, (55 ± 5) %, and a 12-h day-night cycle. The hygiene conditions were inspected regularly to provide an appropriate environment for experimental animals. The operation of animals was limited during the whole experiment. The animals suffered less pain according to the pain classification.

**Figure 1:**
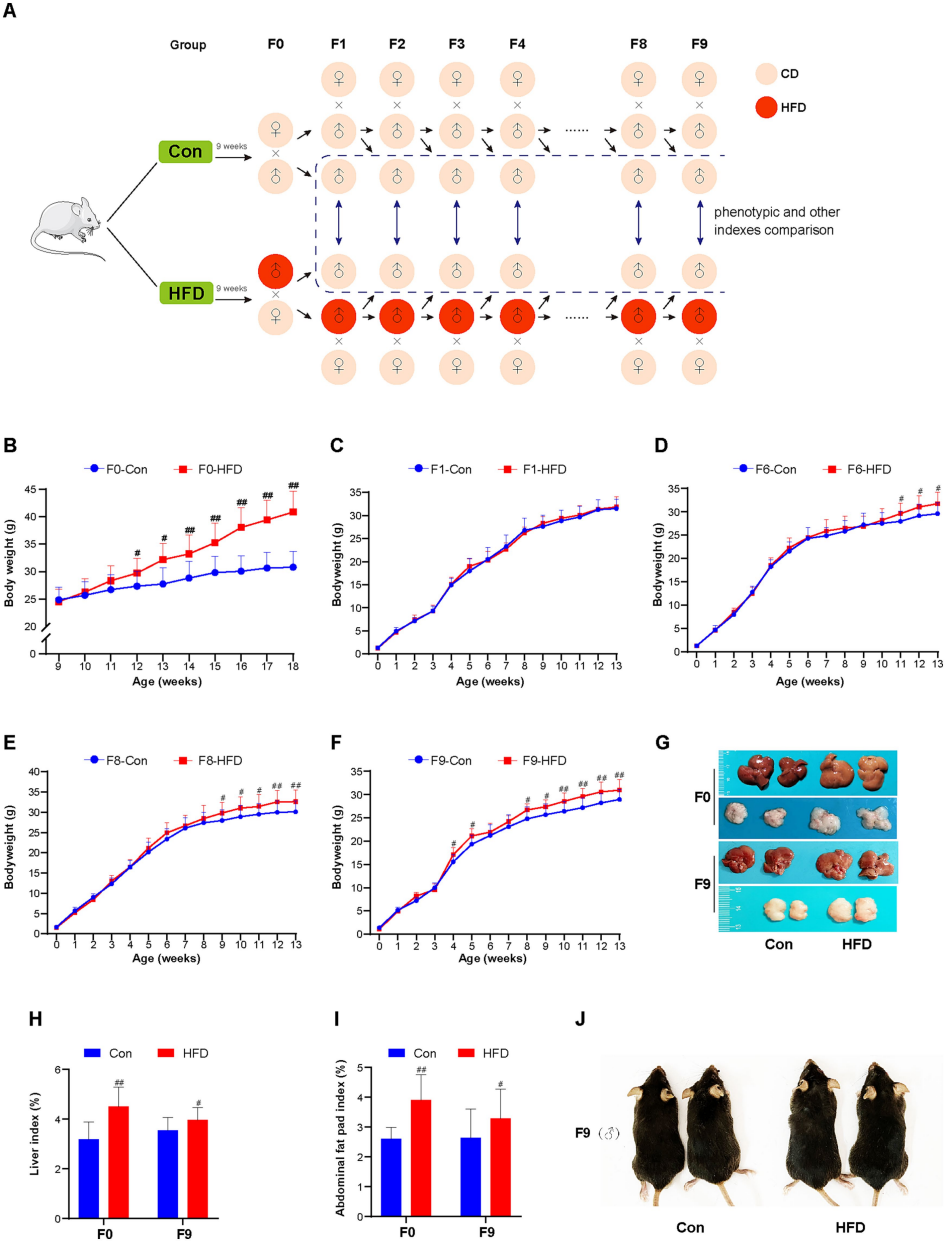
Paternal sustained multi-generational HFD induces obesity phenotype in the founder and offspring. **(A)** Schematic depiction of the experimental protocol. F0 male mice were randomly divided into 2 groups (*n* = 20) fed with CD or HFD. Then the CD/HFD even-weight F0 males were bred with CD-fed, normal-weight independent line females to generate F1 offspring. After weaning, the F1 mice designated for breeding continued on either CD or HFD for 9 weeks, matching their paternal diet. Subsequently, the even-weight F1 males were also bred with CD-fed, normal-weight independent line females to obtain F2 offspring. This breeding and feeding protocol was maintained for subsequent generations. In addition, for the male mice not selected for breeding, they were included in phenotypic analyses and maintained on CD post-weaning. The body weights were recorded weekly. Male offspring body weights were compared between Control (Con) and HFD lineages within the same generation until a statistically significant inter-lineage difference emerged. The other indices of non-reproductive male mice were measured at 12 weeks of age in each generation. **(B)** Body weight trajectories of F0 male mice (Con, HFD: *n* = 20, respectively). **(C–F)** Body weight trajectories of F1, F6, F8 and F9 male mice (F1: Con *n* = 20, HFD *n* = 20; F6: Con *n* = 15, HFD *n* = 17; F8: Con *n* = 19, HFD *n* = 18; F9: Con *n* = 20, HFD *n* = 20). **(G)** Representative images of F0 and F9 mice liver and abdominal fat pad tissue. **(H)** Liver index (liver: body weight) of F0 and F9 mice. **(I)** Abdominal fat pad index (fat pad: body weight) of F0 and F9 mice. **(J)** Representative images of body size changes in F9 mice. The data were presented as mean ± SD, # *p* < 0.05, ## *p* < 0.01 compared with the Con group. CD, control diet; HFD, high-fat diet.

All mice were fed with laboratory basal diet (control diet; SLAC, Changsha, Hunan, China) and water without limitation for 1 week. After the adaptation period, F0 male mice were randomly divided into 2 groups (20 mice per group) fed a control diet (CD; wheat 38.0%, maize 20.0%, soybean powder 18.0%, fish powder 10.0%, wheat bran 5.0%, soybean oil 3.0%, maltodextrin 2.0%, and other 2.0% of minerals and vitamins; The CD contains 69% carbohydrate, 20% protein, and 10% fat) or HFD (79.6% CD, addition of 1.0% cholesterol, 0.2% propylthiouracil, 0.2% bile acid sodium, 5.0% egg yolk powder, 10.0% lard, 4.0% whole milk powder. The HFD contains about 54.5% carbohydrates, 19.5% protein, and 24% fat) (21) (SLAC, Changsha, Hunan, China) to mimic a Western-style fast-food for 9 weeks. Then the CD/HFD even-weight F0 males were bred with CD-fed, normal-weight independent line females, to generate F1 offspring. After weaning, the F1 mice designated for breeding continued on either CD or HFD for 9 weeks, matching their paternal diet. Subsequently, the even-weight F1 males were also bred with CD-fed, normal-weight independent line females to obtain F2 offspring. This breeding and feeding protocol was maintained for subsequent generations. In addition, for the male mice not selected for breeding, they were included in phenotypic analyses and maintained on CD post-weaning. The body weights were recorded weekly. Male offspring body weights were compared between Control (Con) and HFD lineages within the same generation until a statistically significant inter-lineage difference emerged. The other indices of non-reproductive male mice were measured at 12 weeks of age in each generation (Figure 1A). The litter sizes were equalized to the same pup quantity limit between-litter variation due to competition for nutrients postpartum. Mice were anesthetized and euthanized via CO_2_ with gradually increasing concentration to 30% chamber volume /min flow rate and 8–9 L/min volume flowrate according to the volume of the anesthesia box (CL-1000 euthanasia system for small animals, Shanghai Yuyan Instruments Co., Ltd., Shanghai, China) at the end of the experiments period, according to the AVMA guidelines for the euthanasia of animals. Respiration and eye color of each animal were observed continuously. At least 2–3 min of sufficiently high concentration CO_2_ was maintained after cessation of breathing in all animals. A calibrated CO₂ concentration monitoring module was employed to track and record real-time variations in gas concentration within the chamber. Flow meters were regularly calibrated to ensure accurate flow rates; blood and tissues were collected for subsequent measurement or stored at −80 °C.

### 2.2 Glucose and insulin tolerance tests

The oral glucose tolerance test (OGTT) was conducted following an overnight fast. Mouse feces should be removed immediately to prevent coprophagia. The mice received an oral gavage of glucose (2 g/kg body weight; Sigma Aldrich Co., St. Louis, MO, United States). The blood glucose levels were measured from the tail caudal vein using Accu Check Advantage Glucometer (Roche, Basel, Switzerland) at 0, 15, 30, 60, and 120 min post-administration. Serum insulin concentrations were determined using an ELISA kit (Jianglaibio Co., Shanghai, China). The insulin tolerance test (ITT) was performed after a 5-h fast. The mice were administered intraperitoneally insulin (Actapid; Novo Nordisk, Bagsvaerd, Denmark) 0.75 U/kg body weight. Tail blood glucose was measured at 0, 30, 60, 90, and 120 min post-injection. The area under the curve (AUC) was calculated using the trapezoidal method.

### 2.3 Biochemical assays

Blood was collected by eyeball enucleation after anesthesia and stored overnight at 4 °C. Serum was separated by centrifugation at 1,500 × g for 15 min at 4 °C. For the liver tissue, weighed samples were homogenized in 9 volumes of cold normal saline. Homogenates were centrifuged at 1,500 × g for 10 min at 4 °C, and supernatants were collected. The protein concentration was determined using the bicinchoninic acid (BCA) assay. The biochemical parameters of serum or tissue supernatant were measured and detected using commercial assay kits and automated chemistry analyzer (Rayto Life and Analytical Sciences Co., Ltd., Shenzhen, Guangdong, China), including triglyceride (TG), total cholesterol (TC), high-density lipoprotein cholesterol (HDL-C), low-density lipoprotein cholesterol (LDL-C) and glycosylated serum protein (GSP) level.

### 2.4 Histopathological analysis

The liver tissues were fixed in 4% (v/v) paraformaldehyde solution (pH 7.0–7.5) for 24 h, while abdominal adipose tissue was fixed in fat fixative for 24 h. Then the tissues were dehydrated through a graded ethanol series and were embedded in melted wax. Solidified paraffin blocks were sectioned at 4 μm thick slices. After dewaxing, sections were stained with hematoxylin and eosin (H&E; Solarbio Science & Technology Co., Beijing, China). The fixed and dehydrated liver tissues were embedded in optimal cutting temperature compound (Sakura, Torrance, CA, United States) and sectioned into slices, followed by Oil Red O stain. Histopathological images were captured using a microscopy imaging system (Nikon Corporation, Tokyo, Japan).

### 2.5 RNA isolation and microarray expression profiling

The liver tissues RNA isolation, microarray analysis were performed by Aksomics Co. (Shanghai, China) using the Mouse 12 × 135 K Gene Expression Array (Roche NimbleGen, Basel, Switzerland). Liver tissues were ground with liquid nitrogen pre-cooled mortars and pestles. Total RNA for microarray analysis was extracted from liver powder using Transzol Up (Transgen, Beijing, China). RNA quantity and quality were measured by NanoDrop ND-1000. RNA integrity was assessed using standard denaturing agarose gel electrophoresis. Double-strand cDNA (ds-cDNA) was synthesized from 5 μg of total RNA using an Invitrogen SuperScript ds-cDNA synthesis kit (Invitrogen, Carlsbad, CA, United States). ds-cDNA was cleaned and labeled in accordance with the NimbleGen Gene Expression Analysis protocol (NimbleGen Systems, Inc., Madison, WI, United States). Microarrays were hybridized with Cy3-labeled ds-cDNA in NimbleGen hybridization buffer/hybridization component A in a hybridization chamber (Hybridization System-NimbleGen Systems, Inc., Madison, WI, United States). After being washed in an ozone-free environment, the slides were scanned using the Axon GenePix 4000B microarray scanner (Molecular Devices Co., Sunnyvale, CA, United States). Expression data were normalized through quantile normalization and the Robust Multichip Average (RMA) algorithm included in the NimbleScan software. All gene-level files were imported into Agilent GeneSpring GX software (version 12.1; Agilent Technologies Inc., Santa Clara, CA, USA) for further analysis (22). Differentially expressed gene (DEG) was identified via t-test filtering between two groups. Threshold of *p*-value < 0.05 and fold change > 1.5 or fold change < 0.667 was designated as significant DEGs.

### 2.6 Real-time quantitative PCR and Western blot

The hepatic total RNA was reverse-transcribed into cDNA (cDNA reverse transcription kits, TransGen Biotech Co., Ltd., Beijing, China), followed by a real-time quantitative polymerase chain reaction (RT-qPCR) using the CFX96 Real-Time PCR system (Bio-Rad Laboratories Inc., Hercules, CA, United States) and SYBR Select Master Mix (TransGen Biotech Co., Ltd., Beijing, China) according to protocol. The PCR conditions and relative gene expression analysis were performed according to published methods (23). The liver tissues (100 mg) were ground in a liquid nitrogen pre-cooled mortar, and the total protein was extracted with RIPA buffer. The concentrations of protein were determined using the bicinchoninic acid (BCA) protein assay kit (Beyotime Biotech Inc., Shanghai, China) with a Nanodrop ultramicro spectrophotometer instrument. The polyclonal antibody SPNS2, LONP1, HK1, *β*-ACTIN, and anti-mouse/rabbit IgG HRP conjugate were purchased from Abcam Inc. (Waltham, MA, United States) and Cell Signaling Technology, Inc. (Danvers, MA, United States). The methods and processes of SDS-PAGE gel electrophoresis, membrane transfer, antibody incubation, imaging, and relative expression quantity of the target protein were performed as described previously (21). The same samples used for microarray analysis were used for RT-qPCR and Western blot analysis. Primers of genes were designed using Primer Premier 6.0 software (Premier Ltd., Canada), the sequences of them are listed as follows: I*rs3*: forward 5′-AGC CCA AGT ATG AGG ACC GAA TG-3′, reverse 5′-GCA CAG AGC CCA GCA TAG GAA AC-3′; *Slc2a4*: forward 5′-GAC TGA CTC CAT ACA GCC TAC TG-3′, reverse 5′-TAG ATG CTA ATC CCA AGA CAG CC-3′; *Prkag3*: forward 5′-TCC TGA CTA TGA CCG AAG CCA GTG-3′, reverse 5′-GCA TTG CTC TGC GTG ATC TTG TG-3′; *Apoe*: forward 5′-GAA GCC TAT GTC AAC GCC TCT G-3′, reverse 5’-TGC AAG ATT CGA TGG TCT AGT TCC-3′; *Pdx1*: forward 5′-CGG ACA GGA GAC AGT CAA GGA AG-3′, reverse 5′-CGC AGC GTG GTG ATG GAG AA-3′; *Vamp2*: forward 5′-TGT CTA TGG TTC CCT GGC TTC TGT-3′, reverse 5’-ATC CTG GTG TGG CGT CTT GTG T-3′; *Cpt1a*: forward 5’-TCA AAG ACT GAA AGA CTC CTG G-3′, reverse 5′-TTT TCT CCA ACA CAA CGA TGA CG-3′; *Pparg*: forward 5′-GCG AGG GCA AGC AGA CGG ATA TAG-3′, reverse 5′-CTG TGG TTC AGG CAG CGG GAA A-3′; *Inppl1*: forward 5′-AGC CTC CTG CGA CCT CAC AA-3′, reverse 5′-TCC TGC TGC CAC CGA ATG TTG-3′; *Irak1*: forward 5′-CGC TTC CCT CAT CCT CCT GCT ACA-3′, reverse 5′-TTC ACT TCC TCC TCG GTG GCT TCC-3′; *Lrp1*: forward 5′-GGC TGT GCT CTG AAT GAC TCT CC-3′, reverse 5′-TGG TTC CTG TCG TCC AGT AGA TC-3′; *Atb1p3*: forward 5′-CCA TCC GCT TGC TCC TGG AAT AC-3′, reverse 5′-TGC TCT GGG TGA CCT TGT GTG AC-3′; *Spns2*: forward 5′-GGT CCC AGC CAC TAA GAG AG-3′, reverse 5′-GAG CAC GGT GAA GAT AGA GG-3′; *Lonp1*: forward 5′-AGA AGC GTG TCC TGG AGT TCA TTG C-3′, reverse 5′-CTG CCA CAT CTG TCA TGC CAC CAA C-3′; *Hk1*: forward 5′-CCT CCG TCA AGA TGC TGC CAA C-3′, reverse 5′-GGT GTC GTA GAC CTC AGA CTC CAT −3′; *β-actin*: forward 5′-TCA CTA TTG GCA ACG AGC GGT TC-3′, reverse 5′-AGC ACT GTG TTG GCA TAG AGG TCT-3′.

### 2.7 Sperm isolation, DNA extraction, and MeDIP-chip

The caudal epididymis was dissected from sacrificed mice and punctured in pre-warmed (37 °C) sperm isolation buffer (Earle’s Balanced Salt Solution, 25 mM Hepes, 48.5 mM bovine serum albumin) within a Petri dish. After being incubated for 30 min, the supernatant was removed and centrifuged (3,000 g for 5 min), then washed with PBS and water. The samples were incubated in somatic cell lysis buffer, and the supernatant was harvested (24). The sperm used for the methylated DNA immunoprecipitation (MeDIP) study were exactly the fathers of offspring used for microarray expression profiling.

Genomic DNA (gDNA) was extracted using a DNeasy Blood & Tissue Kit (Qiagen, Hilden, Germany). The purified gDNA was quantified and quality assessed by the nanodrop ND-1000. Then the gDNA was sonicated to ~200–1,000 bp with a Bioruptor sonicator (Diagenode, Liege, Belgium). Mouse monoclonal anti-5-methylcytosine (5 mC) antibody (Diagenode) was used for immunoprecipitation of sonicated gDNA. The immunoprecipitated DNA was diluted and purified. The NimbleGen Dual-Color DNA Labeling Kit was used for DNA labeling according to the NimbleGen MeDIP-chip protocol (Nimblegen Systems, United States). Input DNA and MeDIPed DNA were hybridized using Cy3 and Cy5, respectively. And it was hybridized to the Arraystar 4 × 180 K Mouse RefSeq Promoter Microarray chip (Arraystar Inc., Rockville, MD, United States). After the chips were washed, they were scanned by the Agilent Scanner G2505C (Agilent Technologies Inc., Santa Clara, CA, United States). Median-centering, quantile normalization, and linear smoothing were performed by Bioconductor packages Ringo, limma, and MEDME. From the normalized log2-ratio data, a sliding-window (1,500 bp) peak-finding algorithm provided by NimbleScan v2.5 (Roche-NimbleGen) was applied to analyze the MeDIP-chip data. NimbleScan detected peaks by searching for at least 2 probes above a *p*-value minimum cutoff (−log10) of 2. Peaks within 500 bp of each other are merged. The obtained peaks in each sample would be annotated with the information based on the promoter and CpG density. Based on CpG ratio, GC content and CpG-rich region length, promoters are divided into the following three categories: 1. High CpG-density Promoter (HCP): A range of 500 bp region exists in the promoter between −0.7 kb (upstream 0.7 kb) and + 0.2 kb (downstream 0.2 kb) of transcription initiation site (TSS), the (G + C)-fraction in this range is ≥ 0.55, and the observed/expected (O/E) of CpG is ≥ 0.6. 2. Low CpG-density Promoter (LCP): The promoter does not contain the 500 bp length region of CpG O/E ≥ 0.4. 3. Intermediate CpG-density Promoter (ICP): The promoter is not defined as HCP or LCP.

### 2.8 Bioinformatics analysis

Unsupervised hierarchical cluster analysis of DEGs was performed to demonstrate the different patterns displayed in the heat map. Data were pre-standardized as z scores across samples. GO (Gene Ontology) and KEGG (Kyoto Encyclopedia of Genes and Genomes) pathway enrichment analysis (25) of DEGs and differentially methylated genes (DMGs) were performed using R-based on the hypergeometric distribution.

### 2.9 Bisulfite sequencing

The same samples used in microarray analysis were used for bisulfite sequencing. Liver tissues were pulverized with a liquid nitrogen pre-cooled mortar and pestle. Total gDNA was extracted using DNeasy Cell & Blood & Tissue Kit (Servicebio Co., Ltd., Wuhan, China), and was purified and recycled using General-purpose DNA purification and recycling kit (Servicebio). Bisulfite conversion was performed using the Methylation-Gold Kit (ZYMO RESEARCH, Irvine, CA, United States). Converted DNA was amplified by PCR using primers designed with Methprimer software.^1^ The amplification and sequencing region covers the location of the methylation peak of DMGs. The PCR products were cloned into the PCR-TOPO vector using the pSWE-Topo Zero Cloning Kit (Servicebio). Then the receptive bacteria were prepared and transformed. After PCR identification of the bacterial solution, 10 randomly selected clones for each sample underwent sequencing.

### 2.10 Statistical analysis

Each index was measured three times. Statistical analyses were processed by SPSS 25.0 (Chicago, IL., United States) or GraphPad Prism 8 software (La Jolla, CA, United States). Results were presented as the mean ± standard deviation (SD). A two-tailed Student’s t-test was used to determine a significant difference between two groups. *p* < 0.05 was considered statistically significant.

## 3 Results

### 3.1 Paternally multi-generational HFD caused obesity in offspring

Male mice were fed an HFD over multiple generations, and their body weights were recorded weekly. Among F0 founders, HFD-fed mice showed significantly elevated body weight compared to controls from the third week onward. After 9 weeks of HFD consumption, HFD group mice reached a weight of 40.86 ± 3.83 g versus 30.80 ± 2.86 g in the Con group (*p* < 0.01; Figure 1B). HFD led to an increased liver weight ratio and abdominal fat pad accumulation (*p* < 0.01), accompanied by a smoother, greasier, and more yellowish color liver surface (Figures 1G–I). These alterations confirm the successful establishment of the HFD-induced obesity mouse model.

The CD/HFD males were bred with CD-fed females to produce offspring. Body weight differences were compared between Con and HFD lineage mice of each generation, which were not used for breeding but for phenotypic comparison. No significant difference in body weight was observed between the two groups in the F1 generation (Figure 1C). Paternal HFD did not alter the body weight of the first-generation offspring. Notably, it was found that HFD-line descendants exhibited incremental weight accumulation (Supplementary Figure S1), with statistically significant increases first emerging in F6 (Figure 1D). Paternal HFD exposure progressively amplified intergenerational weight gain. Until the F8 and F9 generation offspring, HFD-line mice sustained a post-weaning weight elevation, exceeding controls by 5–9% in adulthood (*p* < 0.01; Figures 1E,F). Mice in the F9 HFD group demonstrated a significant increase in body size (Figure 1J). After dissection, F9 HFD-line mice additionally displayed elevated relative liver mass, abdominal fat pad accumulation (*p* < 0.05), and a slightly white and greasy surface of livers (Figures 1G–I). Those cumulative findings demonstrate the acquired obesity of progeny following sustained paternal-line HFD exposure.

### 3.2 Paternally multi-generational HFD disrupts glucose homeostasis in offspring

**Figure 2:**
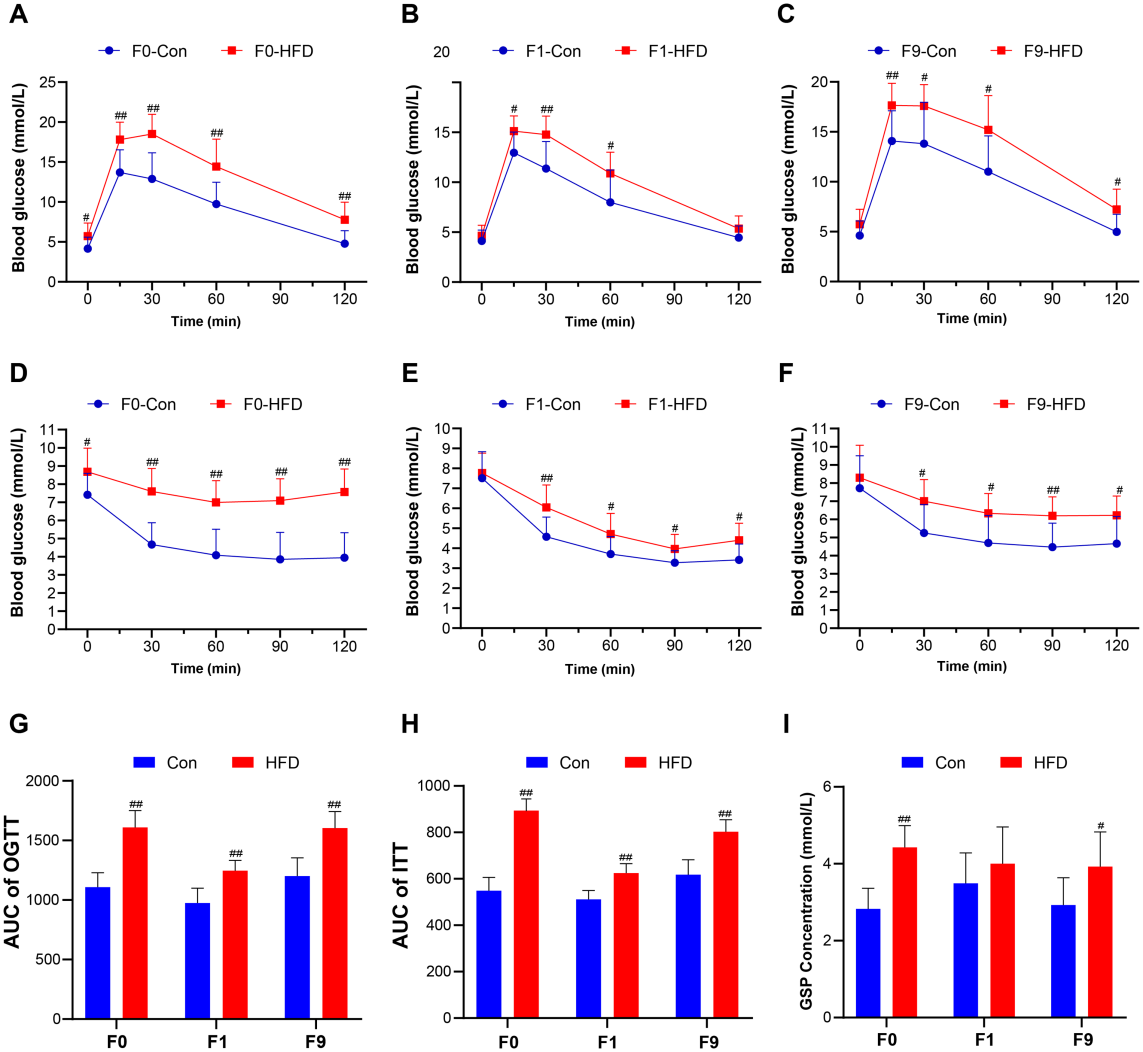
Paternal sustained multi-generational HFD disrupts glucose tolerance and insulin sensitivity in founder and offspring. The OGTT and ITT were performed to evaluate the mice’s glucose homeostasis. **(A–C)** Blood glucose content during OGTT in F0, F1, and F9 male mice (*n* = 10). **(D–F)** Blood glucose content during ITT in F0, F1, and F9 male mice (*n* = 10). **(G)** AUC of OGTT in F0, F1, and F9 male mice. **(H)** AUC of ITT in F0, F1, and F9 male mice. **(I)** Fasting blood GSP concentration of F0, F1, and F9 male mice (*n* = 10). The data were presented as mean ± SD, # *p* < 0.05, ## *p* < 0.01 compared with the Con group. OGTT, Oral glucose tolerance test; ITT, Insulin tolerance test; AUC, Area under the curve; GSP, Glycosylated serum protein.

Glucose homeostasis was assessed via OGTT and ITT. As anticipated, HFD-fed founders exhibited impaired glucose tolerance and reduced insulin sensitivity versus controls (Figures 2A,D). Subsequent generational analysis revealed that HFD induced glucose intolerance (Supplementary Figure S2) and reduced insulin sensitivity (Supplementary Figure S3) in all generations. Different from the obese phenotype, F1 HFD male offspring maintained normal fasting glucose but displayed elevated peak glycemia during OGTT (Figure 2B). Concomitantly, the ITT indicated decreased insulin sensitivity in HFD F1 offspring (Figure 2E). Moreover, obese F9 HFD-line mice demonstrated exacerbated metabolic dysfunction, supported by a higher fasting blood glucose levels (Con 4.60 ± 1.03 mM, HFD 5.74 ± 1.42 mM), glucose peak (Con 14.07 ± 2.89 mM, HFD 17.64 ± 2.1 mM, *p* < 0.01; Figure 2C) and AUC (Con 1201.00 ± 152.60, HFD 1604.00 ± 137.70, *p* < 0.01; Figure 2G) during OGTT, up-regulated fasting GSP (Con 2.93 ± 0.71 mM, HFD 3.93 ± 0.90 mM; Figure 2I), and also accompanied by a more sluggish blood glucose drop rate (Figure 2F) and increased AUC (Con 618.00 ± 64.39, HFD 803.10 ± 51.52, *p* < 0.01; Figure 2H) in ITT. These results illustrate that the sustained paternally multi-generational HFD induces progressive intergenerational dysregulation of glucose homeostasis.

### 3.3 Paternal sustained multi-generational HFD causes dyslipidemia and lipid accumulation in offspring

**Figure 3:**
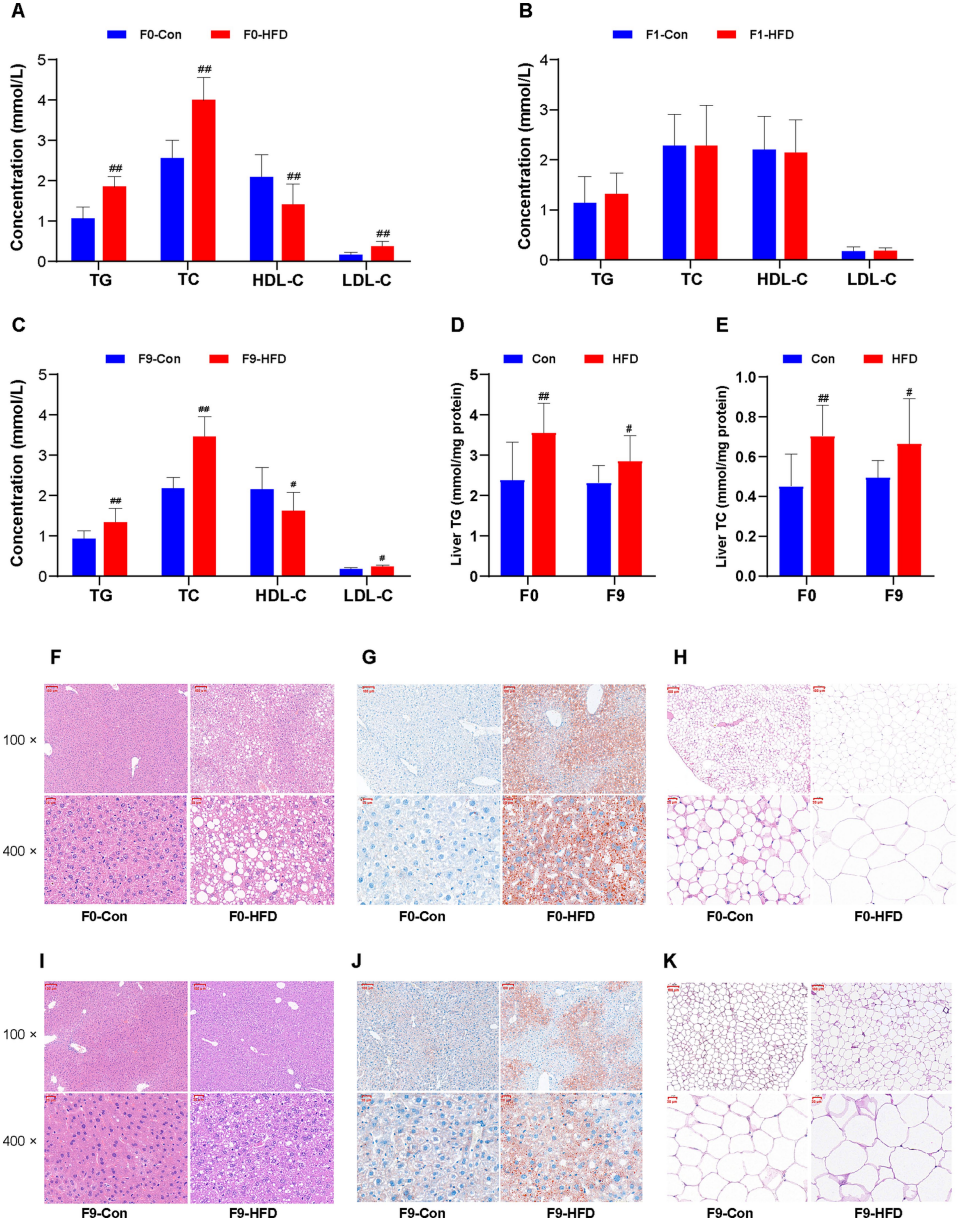
Paternal sustained multi-generational HFD causes dyslipidemia and lipid accumulation in the founder and offspring. The biochemical indices of serum or tissue supernatant were measured and detected using commercial assay kits and an automated chemistry analyzer. The liver and abdominal adipose tissues were stained with H&E or oil red O and photographed at 100 × or 400 × with a microscopy imaging system. **(A–C)** The levels of serum lipids (TG, TC, HDL-C, and LDL-C) in F0, F1, and F9 male mice, respectively (*n* = 10). **(D)** The levels of liver TG in F0 and F9 male mice (*n* = 10). **(E)** The levels of liver TC in F0 and F9 male mice (*n* = 10). **(F,I)** The liver histopathological images stained with H&E of F0 and F9 male mice. **(G,J)** The liver histopathological images stained with oil red O of F0 and F9 male mice. **(H,K)** The abdominal adipose tissues’ histopathological images stained with H&E of F0 and F9 male mice. The data were presented as mean ± SD, # *p* < 0.05, ## *p* < 0.01 compared with the Con group. TG, Triglyceride; TC, Total cholesterol; HDL-C, High-density lipoprotein cholesterol; LDL-C, Low-density lipoprotein cholesterol; H&E, Hematoxylin and eosin.

Serum levels of TG, T-CHO, HDL-C, and LDL-C were analyzed using commercial assay kits and automatic biochemical instruments. The HFD-fed F0 founders displayed obviously increased TG, TC, LDL-C, and reduced HDL-C content (Figure 3A). Similarly, resembling the obese phenotype, F1 offspring showed no significant difference in the blood lipids (Figure 3B), whereas progressive dyslipidemia emerged in subsequent generations. For the HFD F9 offspring that developed obvious obesity, all serum biomarkers were affected significantly, especially the TG (Con 0.97 ± 0.22 mmol/L, HFD 1.38 ± 0.36 mmol/L, *p* < 0.01) and TC (Con 2.22 ± 0.29 mmol/L, HFD 3.50 ± 0.51 mmol/L, *p* < 0.01; Figure 3C). These results demonstrate that sustained paternal-line multigenerational HFD induces intergenerational dyslipidemia.

H&E and Oil Red O staining were used to morphologically assess HFD-induced lipid accumulation in F0 and F9 mice. The histological analysis of HFD F0 livers revealed pronounced hepatic steatosis (accumulation of lipids in the liver) and hypertrophy of abdominal adipose tissue, characterized by the presence of numerous macro vesicular lipid droplets (Figure 3F), expanded oil red O positive staining areas (Figure 3G), hypertrophy, and irregular arrangement of adipocytes (Figure 3H). These pathological features were recapitulated in obese F9 descendants (Figures 3I–K). In addition, consistent with the serum lipids indexes, the hepatic TG (Con 2.32 ± 0.43 mmol/mg protein, HFD 2.85 ± 0.64 mmol/mg protein, *p* < 0.05; Figure 3D), TC (Con 0.50 ± 0.09 mmol/mg protein, HFD 0.67 ± 0.22 mmol/mg protein, *p* < 0.05; Figure 3E) contents were significantly elevated in HFD F9 mice. These biochemical results support the histological analysis.

### 3.4 Paternally multi-generational HFD alters hepatic gene expression profile in offspring

**Figure 4:**
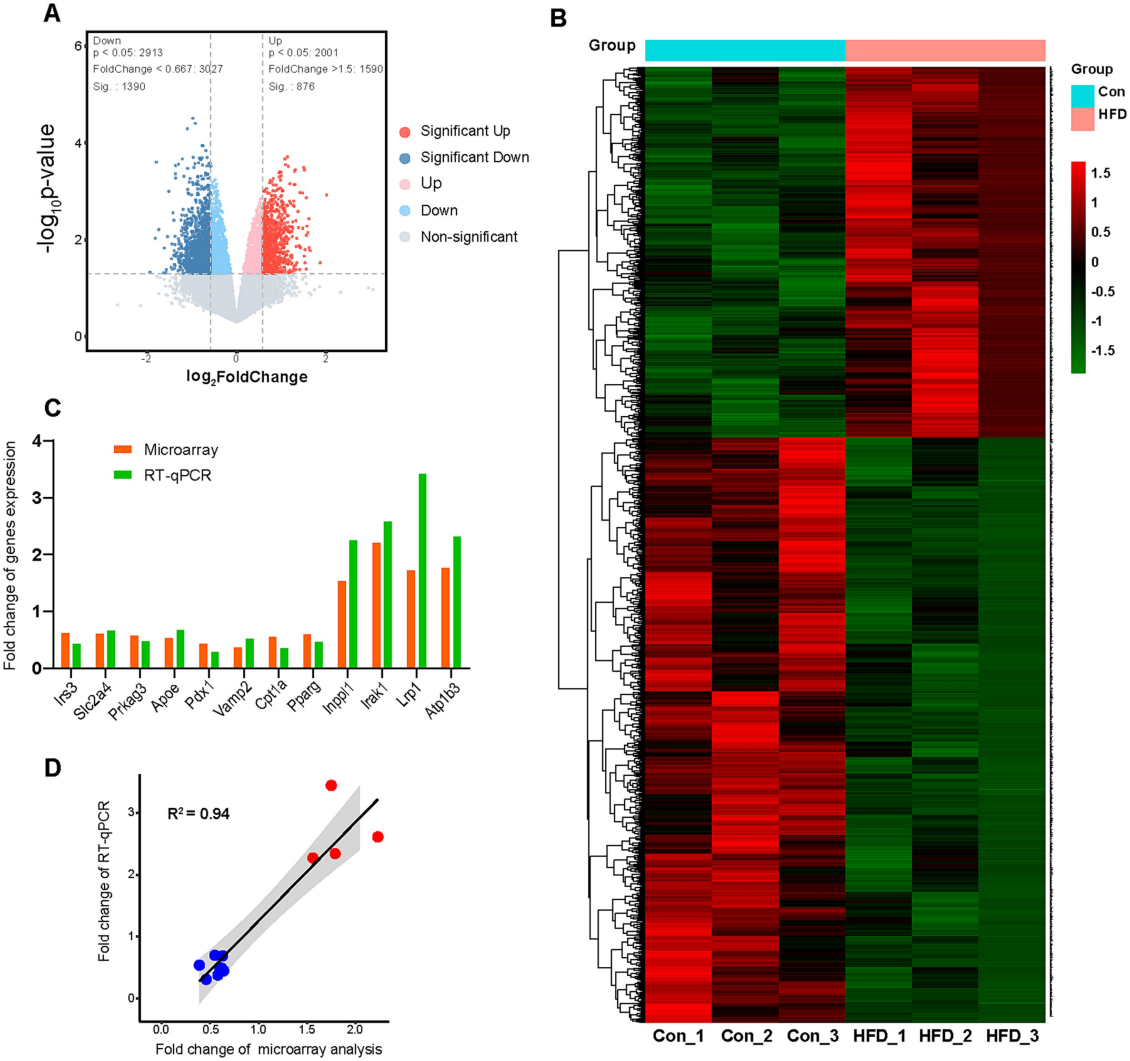
Paternal sustained multi-generational HFD alters hepatic gene expression profile in offspring. The F9 male mice hepatic microarray analyses were performed to identify gene expression profile variation. The expression levels were compared among the Con and HFD line groups (*n* = 3). These genes would be regarded as DEGs if their expression data in the HFD line group were up-regulated at least 1.5 fold or down-regulated less than 0.667 fold compared with Con mice (*p* value < 0.05). Some key genes associated with the glucolipid metabolic process were further validated by RT-qPCR assay. **(A)** The volcano map of DEGs. **(B)** The heat map about unsupervised hierarchical cluster analysis of DEGs. **(C)** The fold change comparison of validated genes in the microarray and RT-qPCR assay. **(D)** Correlation analysis of the Pearson coefficient between microarray and RT-qPCR assay. DEGs, Differentially expressed genes; RT-qPCR, Real-time quantitative polymerase chain reaction.

The hepatic microarray analyses were performed to identify variations in the gene expression profile of F9 offspring. The expression levels were compared among the Con and HFD line groups. Genes were considered differentially expressed genes (DEGs) if their expression in HFD-line mice was either up-regulated (≥1.5-fold) or down-regulated (≤0.667-fold) compared with Con mice, with a concomitant *p*-value < 0.05. A total of 4,000 transcripts were screened, among which approximately 2000 were identified as DEGs. Among these, 876 genes were up-regulated and 1,390 genes were down-regulated in the HFD mice compared to the Con group (Figure 4A). The DEGs’ names, fold change, and other information were listed in Supplementary Table S1. Unsupervised hierarchical clustering of the DEGs revealed distinct expression patterns, as visualized in a heat map (Figure 4B), indicating distinct differences in the hepatic mRNA profile of HFD-exposed F9 offspring. Several key genes associated with the glucolipid metabolic process were selected to be further validated by RT-qPCR assay. The relative expression of insulin receptor substrate 3 (*Irs3*), solute carrier family 2 member 4 (*Slc2a4*), protein kinase, AMP-activated, gamma 3 non-catalytic subunit (*Prkag3*), apolipoprotein E (*Apoe*), pancreatic and duodenal homeobox 1 (*Pdx1*), vesicle-associated membrane protein 2 (*Vamp2*), carnitine palmitoyltransferase 1a (*Cpt1a*) and peroxisome proliferator activated receptor gamma (*Pparg*) in HFD group were found to be down-regulated by 0.45, 0.69, 0.50, 0.70, 0.31, 0.54, 0.38 and 0.49 folds measured by RT-qPCR, while 0.64, 0.63, 0.60, 0.55, 0.46, 0.39, 0.58 and 0.62 from gene chip, respectively (Figure 4C). Up-regulated fold of inositol polyphosphate phosphatase-like 1 (*Inppl1*), interleukin-1 receptor-associated kinase 1 (*Irak1*), low density lipoprotein receptor-related protein 1 (*Lrp1*), and ATPase, Na^+^/K^+^ transporting, beta 3 polypeptide (*Atp1b3*) by HFD were 2.27, 2.61, 3.44, and 2.34 in RT-qPCR, and 1.56, 2.23, 1.75, and 1.79 in gene chip (Figure 4C). Pearson coefficient between microarray expression profile and RT-qPCR in correlation analysis was 0.94 for those certified genes (Figure 4D), indicating the reliability of the microarray assay.

**Figure 5:**
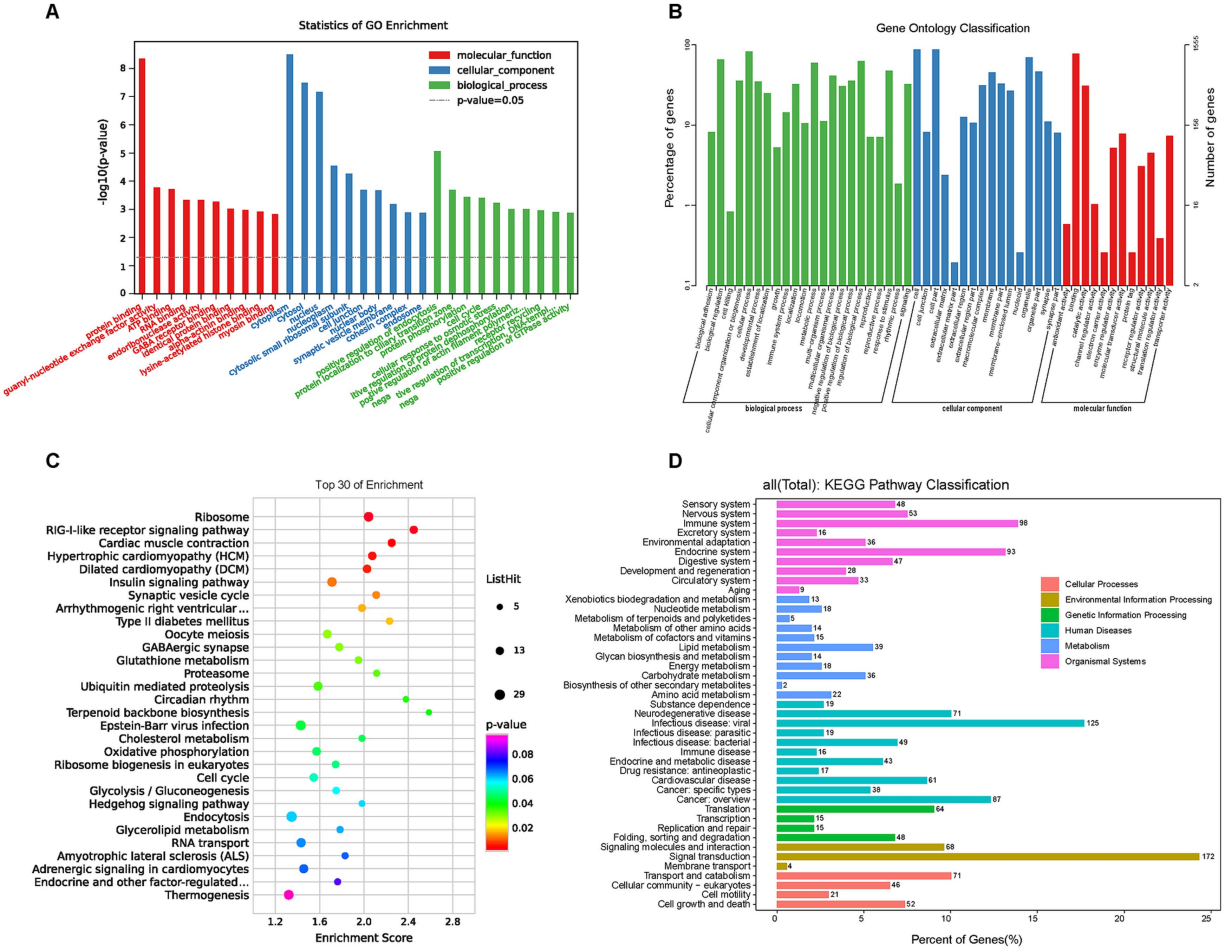
GO and KEGG enrichment analysis of DEGs. GO and KEGG pathway enrichment analysis of DEGs was performed using R-based on the hypergeometric distribution. **(A)** The most significant top 10 GO terms in the three categories (BP, CC, and MF) of DEGs. **(B)** The GO barplot category of DEGs in level 2. **(C)** The KEGG bubble plot for the top 30 signal pathways of DEGs. **(D)** The KEGG classification in level 2 of DEGs. GO, Gene ontology; KEGG, Kyoto encyclopedia of genes and genomes; DEGs, Differentially expressed genes; BP, Biological process; CC, Cellular component; MF, Molecular function.

GO and KEGG enrichment analyses were performed to further investigate signal pathways associated with the DEGs. About 2000 DEGs were mapped to typical terms cytoplasm, cytosol, nucleus, synaptic vesicle membrane (cellular component, CC); protein binding, ATP binding, RNA binding (molecular function, MF), and protein localization, protein phosphorylation, cell cycle, transcription regulation (biological process, BP; Figure 5A). For the level 2 GO enrichment, the DEGs were mainly involved in cell, membrane, organelle (CC), binding, catalytic, enzyme regulator, and translation regulator activity (MF), and biological regulation, cellular process, metabolic process, signaling (BP; Figure 5B). Pathway analysis of DEGs was conducted based on the KEGG database. Notably, canonical pathways associated with obesity and metabolism with the largest number of DEGs were identified, such as the insulin signaling pathway, T2DM, glutathione metabolism, circadian rhythm, cholesterol metabolism, glycolysis/gluconeogenesis, insulin secretion, and AMPK signaling pathway. (Figure 5C). At the level 2 KEGG enrichment, the DEGs were mainly enriched in signal transduction, signaling molecules and interaction (environmental information processing), cardiovascular diseases, endocrine and metabolic diseases (human diseases), endocrine system, immune system (organismal systems), and lipid metabolism, carbohydrate metabolism, energy metabolism (metabolism; Figure 5D). These results indicate that HFD primarily regulates genes related to glucolipid and energy metabolism in offspring.

### 3.5 Paternally multi-generational HFD affects the DNA methylation profile of offspring

**Figure 6:**
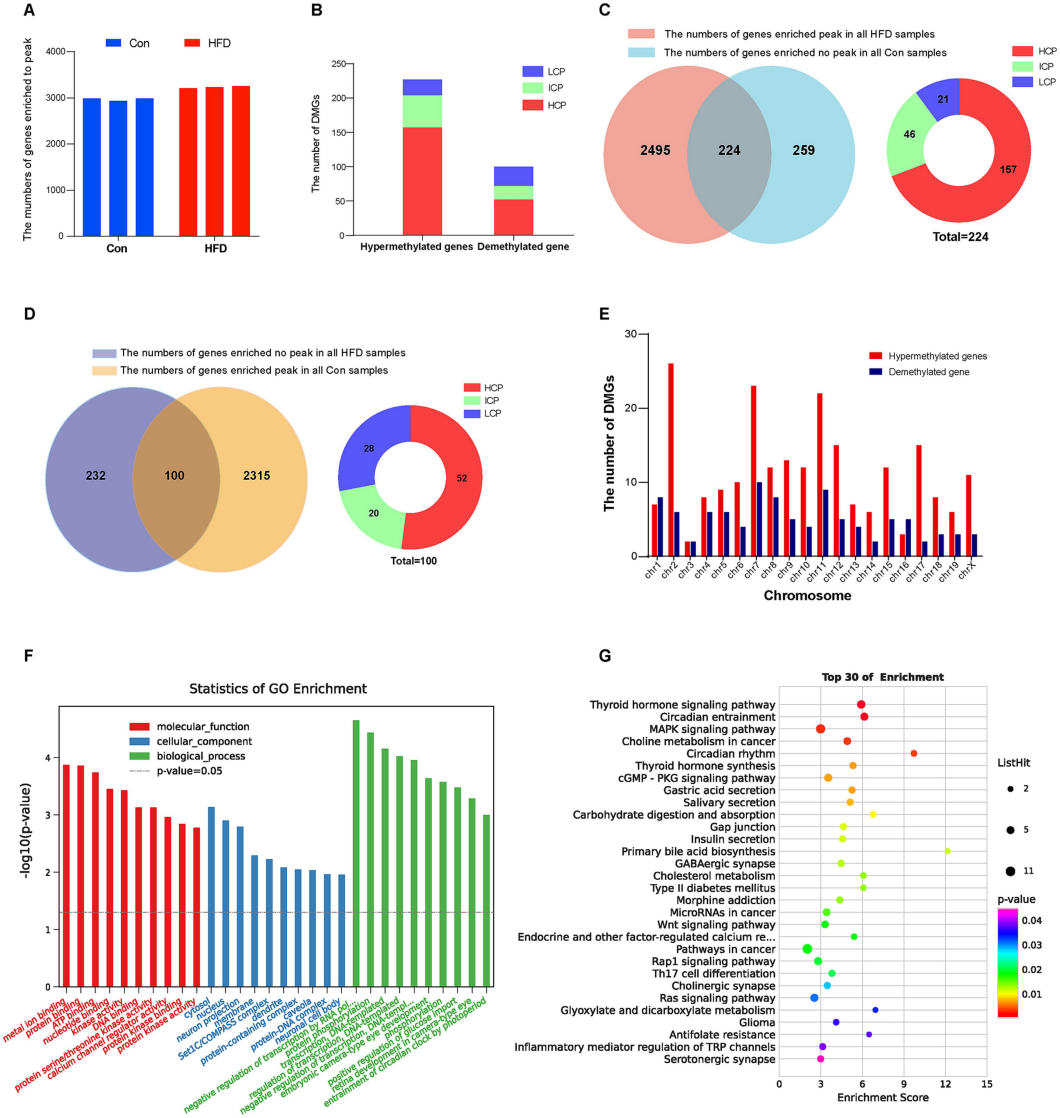
Paternal sustained multi-generational HFD affects the DNA methylation profile in the gene promoter region of offspring. The MeDIP-chip analysis was performed to identify the DNA methylation variation in the gene promoter region of F8 offspring sperm (*n* = 3). The peak-finding algorithm provided by NimbleScan was applied to search for methylated peaks within the genes’ promoter region. The peak in each sample was obtained and annotated with the information based on the promoter and CpG density. GO and KEGG pathway enrichment analysis of DMGs was performed using R-based on the hypergeometric distribution. **(A)** The number of peaks or enriched genes in each sample. **(B)** The number of hypermethylated and demethylated genes induced by HFD. **(C)** Statistics and distribution of HFD-induced hypermethylated genes. **(D)** Statistics and distribution of HFD-induced demethylated genes. **(E)** Distribution of DMGs in the chromosome. **(F)** The most significant top 10 GO terms in the three categories (BP, CC, and MF) of DMGs. **(G)** The KEGG bubble plot for the top 30 signal pathways of DMGs. DMGs, Differentially methylated genes; HCP, High CpG-density promoter; ICP, Intermediate CpG-density promoter; LCP, Low CpG-density promoter.

The MeDIP-chip analysis was performed to identify the DNA methylation variation in the gene promoter region of F8 offspring sperm, which were the fathers of F9 employed in the microarray expression profiling assay. From the MeDIP-chip data, the peak-finding algorithm provided by NimbleScan was applied to search methylated peaks within gene promoters. The peak in each sample was obtained and annotated with the information based on the promoter and CpG density. A total of 3,847 gene promoter regions were enriched at the peak. The number of enriched peaks in each sample from both groups is shown in Figure 6A. For the genes that were enriched to methylated peaks on the promoter region in all three HFD group samples but none in the Con group, they were regarded as being hypermethylated by HFD; otherwise, they were demethylated by HFD. Statistical analysis identified 224 DMGs that were hypermethylated (Figures 6B,C) by HFD, while 100 were demethylated (Figures 6B,D). Most promoter types of DMGs were HCP and ICP (Figures 6C,D). The chromosome distribution of each DMG was shown in Figure 6E. The DEGs with more details, including peak information, were listed in Supplementary Table S2. GO and KEGG enrichment were analyzed to further investigate signal pathways of DMGs. The DMGs mapped to the top 30 GO terms, such as cytosol, nucleus (CC); metal ion binding, protein binding, ATP binding, kinase activity (MF), and regulation of transcription, DNA-templated, protein phosphorylation (BP). (Figure 6F). Similar to DEGs, many DMGs were enriched in insulin secretion, T2DM, circadian rhythm, and cholesterol metabolism pathways associated with obesity and metabolism (Figure 6G). These results indicate that HFD primarily leads to gene promoters’ hypermethylation in offspring related to glucolipid and energy metabolism.

### 3.6 DEGs and DMGs co-analysis of altered methylation of key genes in offspring

**Figure 7:**
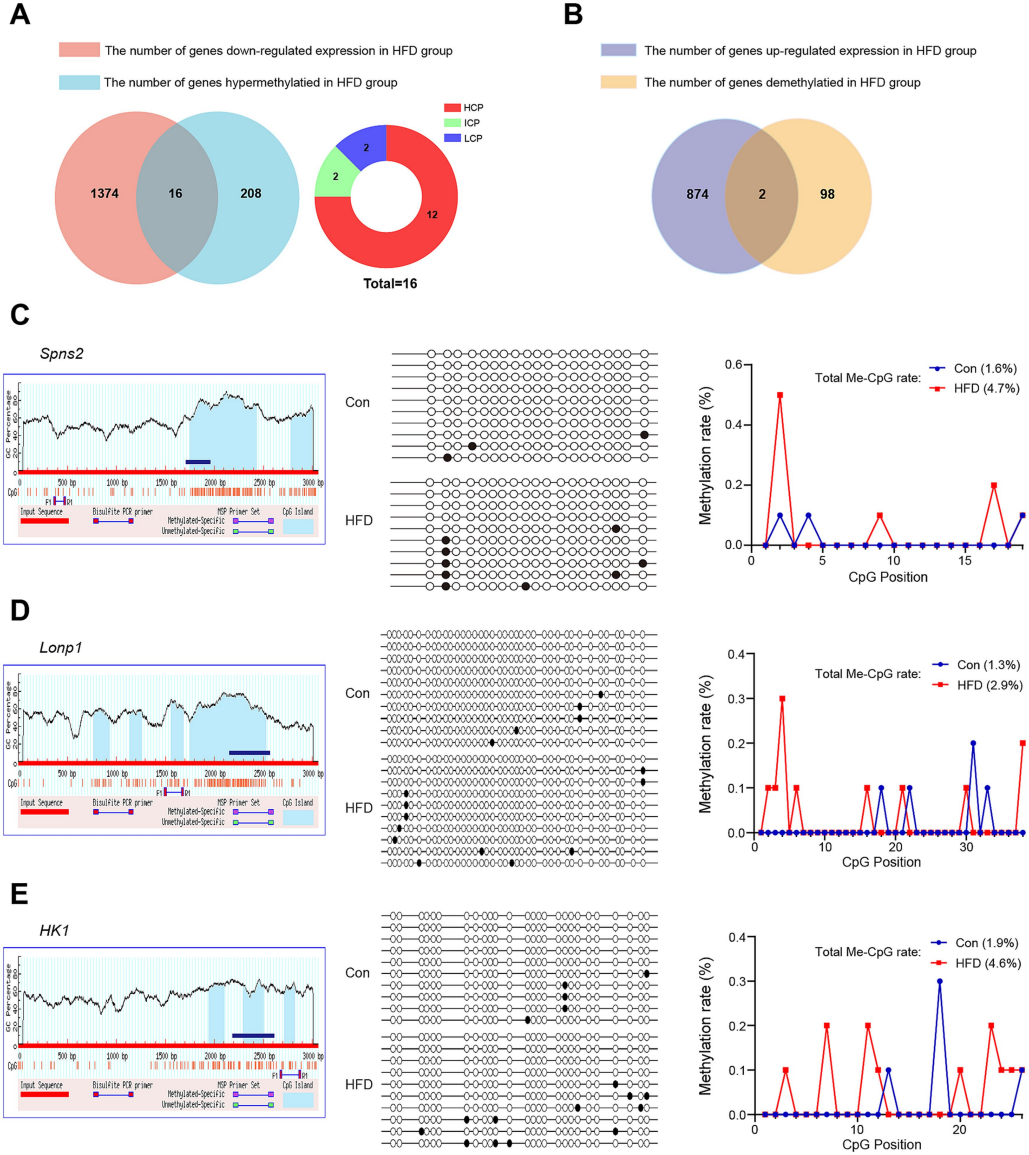
DEGs and DMGs co-analysis and verification of critical genes in offspring. Based on DEGs and DMGs, the genes were selected whose expression pattern was consistent with the promoter methylation trend. The promoter methylation levels of target genes related to glycolipid metabolism (*Spns2*, *Lonp1,* and *Hk1*) in the liver of F9 male mice were further detected by bisulfite sequencing. **(A)** Statistics and distribution of HFD-induced hypermethylated and down-regulated expression genes. **(B)** Statistics of HFD-induced demethylated and up-regulated expression genes. **(C–E)** The bisulfite sequencing region, results, and Me-CpG rate of *Spns2*, *Lonp1,* and *Hk1* genes, respectively. The blue bars represent the sequencing region. White circles represent unmethylated CpGs, while black circles represent methylated CpGs. Me-CpG, Methylated CpG.

**Figure 8:**
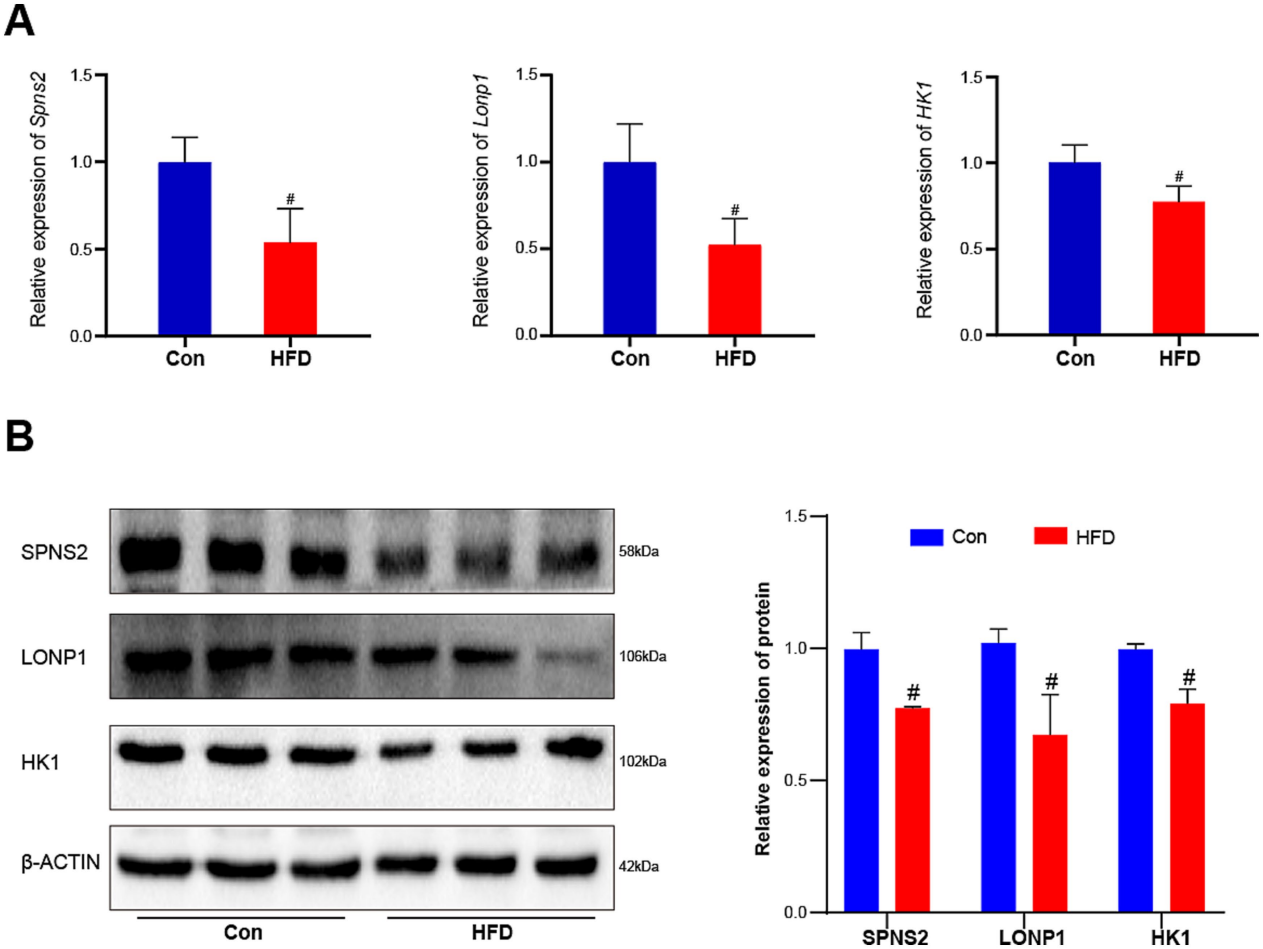
Paternal sustained multi-generational HFD reduces the expression of three critical genes in offspring. The relative expression levels of these target genes’ mRNA and protein in F9 male mice liver were measured by RT-qPCR and western blot (*n* = 3). **(A)** The relative expression levels of *Spns2*, *Lonp1,* and *Hk1* genes’ mRNAs. **(B)** The relative protein expression levels of SPNS2, LONP1, and HK1. The data were presented as mean ± SD, # *p* < 0.05 compared with the Con group.

Transcriptional downregulation positively correlated with promoter hypermethylation levels. Based on DEGs and DMGs, the genes were selected whose expression pattern was consistent with the trend of promoter methylation. There were 16 target genes that exhibited HFD-induced promoter hypermethylation with concordant expression suppression (Figure 7A), while only 2 genes showed an opposite trend (Figure 7B). Most promoter types of target genes were HCP (Figure 7A). More detailed information on these genes is listed in Tables 1, 2. For the F9 offspring, the progeny of sperm used for MeDIP-chip analysis, bisulfite sequencing confirmed elevated methylation at *Spns2*, *Lonp1*, and *Hk1* promoters in HFD-line mice versus controls, validating MeDIP-chip data. Paternal HFD altered some CpG site methylation rates (Figures 7C–E). Correspondingly, the RT-qPCR assay confirmed significant downregulation of *Spns2*, *Lonp 1,* and *Hk1* transcripts of the HFD line F9 mice liver (Figure 8A). Western blot analysis revealed that the protein expression levels of SPNS2, LONP1, and HK1 were significantly decreased in HFD mice; their relative expression levels decreased to 0.77 ± 0.01-fold, 0.67 ± 0.15-fold, and 0.79 ± 0.06-fold compared with the control group (Figure 8B; Supplementary Figure S4). These results verify that changes in DNA methylation and gene expression patterns can be inherited progressively intergenerational by offspring from the paternal generation with HFD consumption.

**Table 1:** The target genes whose expression pattern was consistent with the promoter methylation trend (Hypermethylated and down-regulated expression in HFD group).

Gene ID	Gene name	Promoter classification	Chromosome	Peak start	Peak end	Peak length	Strand	Peak to TSS	TSS	TTS
269,959	Adamtsl3	HCP	chr7	89,483,326	89,484,595	1,269	+	−242	89,484,203	89,762,958
214,230	Pak6	HCP	chr2	118,503,002	118,503,237	235	+	138	118,502,981	118,523,756
216,892	Spns2	HCP	chr11	72,303,357	72,303,796	439	−	−170	72,303,406	72,265,139
69,191	Pdia2	HCP	chr17	26,336,366	26,336,616	250	−	−459	26,336,032	26,332,943
170,625	Snx18	HCP	chr13	114,408,683	114,408,935	252	−	−37	114,408,772	114,382,386
74,142	Lonp1	HCP	chr17	56,765,827	56,766,086	259	−	369	56,766,326	56,753,721
15,275	Hk1	ICP	chr10	61,802,208	61,802,853	645	−	638	61,803,169	61,731,602
668,253	Dleu2	HCP	chr14	62,301,129	62,301,835	706	−	−272	62,301,210	62,221,672
13,388	Dll1	HCP	chr17	15,512,748	15,513,000	252	−	−87	15,512,787	15,504,317
238,988	Erc2	HCP	chr14	28,435,505	28,435,765	260	+	8	28,435,627	29,291,723
208,439	Klhl29	HCP	chr12	5,382,002	5,382,413	411	−	280	5,382,488	5,084,273
71,602	Myo1e	HCP	chr9	70,055,051	70,055,490	439	+	114	70,055,156	70,247,874
66,320	Tmem208	HCP	chr8	107,849,379	107,849,633	254	+	−757	107,850,263	107,852,957
240,186	Zfp438	HCP	chr18	5,333,951	5,334,395	444	−	264	5,334,437	5,210,028
320,111	Prr18	ICP	chr17	8,533,831	8,534,080	249	+	352	8,533,603	8,536,978
18,071	Nhlh1	LCP	chr1	173,987,241	173,987,487	246	−	363	173,987,727	173,982,422

**Table 2:** The target genes whose expression pattern was consistent with the promoter methylation trend (Demethylated and up-regulated expression in HFD group).

Gene ID	Gene name	Promoter classification	Chromosome	Peak start	Peak end	Peak length	Strand	Peak to TSS	TSS	TTS
66,251	Arfgap3	HCP	chr15	83,180,217	83,180,463	246	-	337	83,180,677	83,130,169
328,580	Tubgcp6	HCP	chr15	88,954,110	88,954,365	255	-	−657	88,953,580	88,929,527

## 4 Discussion

**Figure 9:**
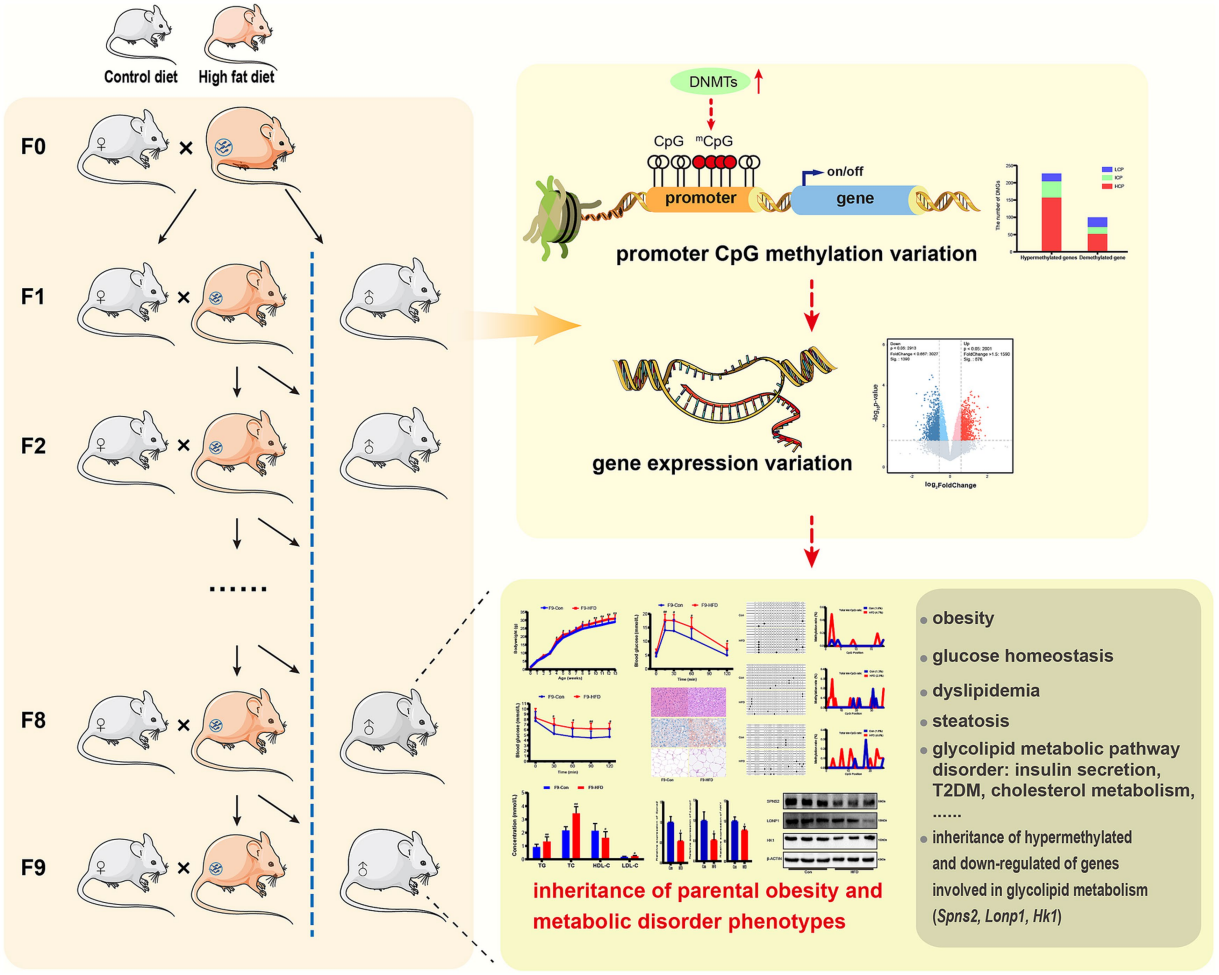
Paternally multi-generational high-fat diet causes obesity and metabolic disorder through intergenerational DNA methylation. We performed a novel paternally multi-generational HFD consumption model in male C57BL/6J mice, while excluding maternal gestational effects and any confounding influences from females. The CD/HFD males were bred with CD-fed to obtain offspring, for the male mice not selected for breeding were included in phenotypic analyses and maintained on CD post-weaning. MeDIP/gene-chip, results indicated that paternal HFD significantly modified gene expression and DNA methylation profiles in liver or sperm of offspring. Majority of differential genes exhibited hypermethylation in promoter regions and reduced expression in liver, which were linked to glucolipid metabolic signaling pathway, and elevated promoter methylation and expression states of genes implicated in glycolipid metabolism (Spns2, Lonp1 and Hk1) were inherited by offsprings. These HFD induced changes caused intergenerational accumulation of body weight increase, disturbance of glycolipid metabolism and insulin insensitivity in male offspring. These results show that paternal sustained multi-generational HFD could induce intergenerational progressive accumulation of obesity and metabolic disorder through DNA methylation regulation.

This study provides evidence that paternal multi-generational HFD could cause intergenerational progressive accumulation of obesity and glycolipid metabolic disorders in offspring, which is mediated by heritable altered DNA methylation information and gene expression profiles (Figure 9).

Previous study supports that parental dietary factors induce disease phenotypes inheritance through non-genetic epigenetic mechanisms (26, 27). Our research specifically explores paternal multigenerational HFD and its progressive intergenerational genetic effect on obesity and metabolic disorders. Disruptions in epigenetic regulators, including DNA methylation, histone modifications, and non-coding RNA, constitute potential pathways for obesity pathogenesis (28). Moreover, ancestral environmental insults could confer obesity susceptibility to offspring through epigenetic mechanisms (29). Maternal obesity is positively correlated with offspring birth weight, obesity, and metabolic complications. For example, offspring of mice fed a high-fat diet during late pregnancy increased body weight, food intake, insulin resistance, and lipid levels (30). Maternal high-fat diet also exacerbated obesity in female offspring, accompanied by increased serum leptin levels, decreased insulin sensitivity (18), destruction of adipose tissue structure (31), and metabolic and reproductive hormone disorders (32). However, most studies examining HFD-induced metabolic dysregulation focus on maternal diet during the pregestational or perinatal period (17, 18, 31–34). Therefore, the conclusions of this type of study are essentially confounded with direct exposure in utero to maternal adipose tissue, which makes it difficult to determine whether parental diet causes changes in the offspring’s epigenetic patterns and phenotypes through epigenetic modifications in the gametes, or whether the adverse phenotype is a direct effect of diet on the fetus or the offspring itself. The latter does not constitute inheritance through epigenetic mechanisms (35). Additionally, cyclic endocrine variables of female animals introduce further experimental noise. Therefore, the paternal line HFD and male animal provide an optimal model system that eliminates maternal-generation confounders. Furthermore, some studies report paternal diet (19) or prediabetes (20) disrupted offspring glucose homeostasis without significant descendant obesity, and others document inconsistent metabolic phenotypes (36, 37). In this research, a mouse model of paternal sustained multi-generational feeding with HFD was performed to simulate western-diet-preferring obese families, amplification and accumulation of obesity epidemic inheritance in HFD line progeny, and to explore the epigenetic mechanism driving this progression. Consistent with previous studies, no difference in body weight gain was detected between male mice of the F1 or F2 generation on the HFD, in contrast the increase in offspring body weight resulting from maternal obesity (30) or high-calorie diet (38), suggesting that the direct effect of the maternal diet on theus appears to contribute more to the observed maternal intergenerational effects than epigenetic mechanisms-mediated pathways. The OGTT test is designed to assess the ability of beta cells to secrete insulin, which involves the physiological response of “blood glucose elevation→pancreatic beta cells sense the glucose concentration→release insulin.” It can determine whether the beta cells can secrete insulin quickly and in sufficient quantities to cope with elevated blood. The ITT test, by injecting exogenous insulin intravenously, artificially creates a high insulin state, and observes the rate of decline in blood glucose, which involves the response of “exogenous insulin→activates glucose receptors in tissues such as muscle and fat→promotes glucose into cells→blood glucose decreases.” The ITT test can determine insulin, such as the function of insulin receptors on the cell surface, the efficiency of signal transduction, and the ability of glucose transport. The two tests complement each other and can reveal a complete picture of the entire glucose metabolism chain. In this study, we found that paternally multi-generational HFD caused mild insulin insensitivity and glucose intolerance. This suggests that a few generations of HFD can disrupt glucose-insulin homeostasis but is not sufficient to induce offspring obesity. Excitingly, our research finally observed a progressive weight gain, culminating in significantly elevated body mass in F8 and F9 HFD-line descendants. This robust evidence demonstrates that paternal HFD damage undergoes cumulative, ultimately manifesting as intergenerational obesity. Our results extend previous findings and are an important foundation for solving the most critical technical problem of the subsequent study.

Obesity promotes hyperlipidemia and excessive lipid accumulation, elevating cardiovascular and metabolic syndrome risk, including insulin resistance (39). Releasing lipids could perturb the intracellular concentration of intermediates (ceramide and other lipids), causing impairment of cellular insulin responsiveness by inhibiting the insulin signaling cascade downstream components (insulin receptor, insulin receptor substrate (IRS), or AKT) (40). The liver is central to glucose homeostasis and triacylglycerol metabolism, developing steatosis in obesity, correlating with systemic insulin resistance in hepatic and muscle tissues (41, 42), so it is therefore pivotal for elucidating metabolic dysregulation mechanisms. Our integrated analysis of serum, hepatic, and visceral adipose tissues via biochemical assays and histopathology revealed lipid accumulation patterns paralleling obesity progression. These results demonstrate that paternal HFD produces intergenerational dyslipidemia and tissue steatosis in offspring, and provide mechanistic corroboration for the observed increase in progeny body mass.

Organisms could generate phenotypic diversity through epigenetic modifications without altering primary DNA sequences. Epigenetic markers constitute regulatory codes that govern genomic activity. During development, epigenetic reprogramming occurs at gametogenesis and fertilization, with erasure of these marks enabling cellular totipotency (43). Notably, accumulating evidence demonstrates incomplete epigenetic erasure. Environmental factors alter germ cell epigenomes, and marks escaping reprogramming or not being re-established may persist as permanent modifications. These heritable epigenetic changes can be transmitted to offspring, establishing novel transcriptional programs that confer acquired phenotypic traits. This mechanism provides a molecular basis for Lamarckian-like generational epigenetic inheritance (44). Epigenetics involving histone modification (45) and non-coding RNAs (37, 46) has been indicated in intergenerational inheritance induced by HFD. A maternal high-fat diet can induce histone modification. In the livers of the offspring fed a high-fat diet during pregnancy and lactation, the levels of Wnt1 and beta-Catenin, the acetylation of H4 and H3 were reduced, and the methylation of H3K9 in the encoding Wnt1 was increased (47). The female mice fed with HFD showed reduced adiponectin expression and H3K9 acetylation in their adipose tissue, along with increased H4K20 methylation in the promoter region of leptin and increased H3K9 methylation in the promoter region of adiponectin (48). HFD exposure also alters DNA methylation patterns in genes predisposing to obesity and metabolic disorders (49), with partial retention of these disrupted methylation/expression profiles in subsequent generations’ germ cells, especially for the paternal HFD intervention (16). It is generally assumed that fathers contribute to the next generation in a relatively simple manner, limited to half of the genome. However, this seems to be an underestimation. Epigenetic marks carried by sperm, including DNA methylation (50), chromatin modifications (51), RNA molecules (52), and accumulated in the father, may be transmitted to the offspring, influencing their phenotype (53). In the present study, we observed obesity in offspring of paternal HFD lineages. We propose that this phenotype originates from sperm-borne DNA methylation marks, as MeDIP-chip analysis revealed distinct sperm methylomes in obese descendants: promoter hypermethylation predominated among DMGs. These DMGs regulate critical pathways, including insulin secretion, T2DM, circadian rhythm, and cholesterol metabolism pathways. Associated with obesity and metabolic disturbance. This systematic promoter methylation dysregulation in sperm of HFD-lineage offspring demonstrates the comprehensive epigenetic reprogramming underlying the accumulation of intergenerational metabolic disorders.

DNA methylation exerts biological functions by regulating gene expression. The 5 mC in CpG islands blocks the transcription factor complexes from binding to promoters. It also recruits methyl-CpG binding proteins, forming transcriptionally repressive “closed” chromatin conformations. Thereby, promoter hypermethylation typically silences genes, while hypomethylation potentiates expression (54). The combination of HFD and exercise may promote genomic-scale reprogramming of hepatic DNA methylation and gene expression profiles, in particular affecting those involved in carbohydrate/lipid metabolism and muscle developmental processes (55). The study investigated hepatic gene expression profile variation in F9 offspring, which were the progeny of F8 fathers analyzed by MeDIP. HFD-line F9 mice exhibited distinct mRNA expression profiles, with microarray data revealing differential expression in obesity-metabolism pathways, including the insulin signaling pathway, T2DM, glutathione metabolism, circadian rhythm, cholesterol metabolism, glycolysis/gluconeogenesis, insulin secretion, and AMPK signaling pathway. More importantly, to establish a causal link between gene promoter DNA methylation and gene expression, we identified genes exhibiting concordant DNA methylation and mRNA expression changes. Most target genes were hypermethylated with concomitant expression suppression. Specifically, mirroring methylation states in paternal F8 sperm, higher promoter methylation in F9 HFD-line livers was observed for lipid/glucose metabolism genes facilitating lipid transport, lipid metabolism, and glucose glycolysis, such as *Spns2* (56, 57), *Lonp1* (58), and *Hk1* (59, 60), with corresponding mRNA and protein expression downregulation. Significantly, this means that paternal HFD causes gene hypermethylation that exhibits a partial resistance to global demethylation postfertilization and potentially inherits methylation from pregeneration sperm to next generation somatic tissues, which may confer a predisposition to obesity and metabolic resistance. The findings indicate a mechanism for intergenerational transmission of CpG methylation, which subsequently modulates gene expression and mediates the progressive accumulation of obesity and metabolic dysfunction across generations. Collectively, these studies provide evidence that paternal HFD induces intergenerational transmission of DNA methylation-driven phenotypes and identify the target genes related to the intergenerational reprogramming.

## 5 Conclusion

In summary, using a unique paternal sustained multi-generational HFD model, this research demonstrates a significant and persistent increase in intergenerational progressive accumulation in offspring body weight and metabolic dysregulation. The observed alterations in promoter DNA methylation and gene expression, particularly in genes implicated in glycolipid metabolism, provide a potential mechanistic explanation. Our findings elucidate how HFD-induced CpG methylation patterns intergenerational transmission modifies gene expression to drive the cumulative obesity and metabolic disruption across generations. Our study expands current understanding of dietary intergenerational inheritance of increased disease risk, and underscores the importance of paternal dietary interventions to mitigate metabolic risks in offspring.

## Data Availability

The original contributions presented in the study are included in the article/Supplementary material; further inquiries can be directed to the corresponding author.

## Footnotes

^1^ www.urogene.org/methprimer/index1.html

## Glossary

AUC: area under the curve
BP: biological process
CC: cellular component
CD: control diet
CVD: cardiovascular disease
DMGs: differentially methylated genes
ds-cDNA: double-strand cDNA
DEG: differentially expressed gene
gDNA: genomic DNA
GO: Gene Ontology
GSP: glycosylated serum protein
HCP: high CpG-density promoter
HDL-C: high-density lipoprotein cholesterol
H&E: hematoxylin and eosin
HFD: high fat diet
ICP: intermediate CpG-density promoter
IRS: insulin receptor substrate
KEGG: Kyoto Encyclopedia of Genes and Genomes
LCP: low CpG-density promoter
LDL-C: low-density lipoprotein cholesterol
MeDIP: methylated DNA immunoprecipitaton
MF: molecular function
O/E: observed/expected
OGTT: oral glucose tolerance test
ITT: insulin tolerance test
RMA: robust multichip average
PPI: protein–protein interactions
RT-qPCR: real-time quantitative polymerase chain reaction
T2DM: type 2 diabetes mellitus
TC: total cholesterol
TG: triglyceride
T-DEG: target DEG
TSS: transcription initiation site
