# Supplementary material for: Paternally multi-generational high-fat diet causes obesity and metabolic disorder through intergenerational DNA methylation
Source: Front Nutr. 2025 Oct 27;12:1680793. doi: 10.3389/fnut.2025.1680793 (PMC12597747; doi:10.3389/fnut.2025.1680793)
Supplement: Supplementary file 1 [file Table_1.DOCX]

Supplementary Material

# Supplementary Figures and Tables

## Supplementary Figures


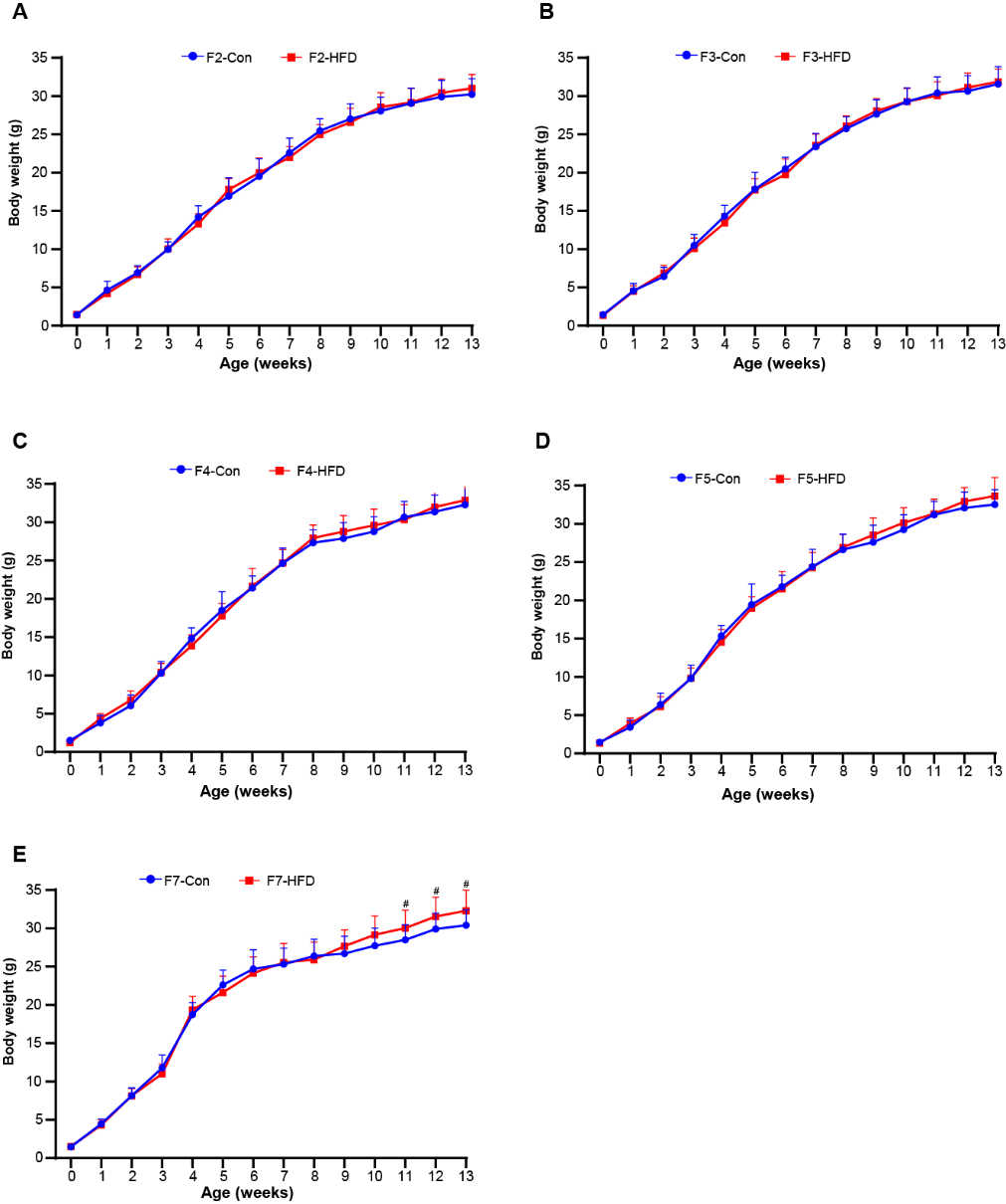


**Supplementary Figure 1.** Paternal sustained multi-generational HFD induces accumulation of incremental weight tendency in subsequent generation HFD offspring. A-E: Body weight trajectories of F2, F3, F4, F5 and F7 male mice (Con: n = 17, 16, 18, 19 and 17, respectively; HFD: n = 18, 17, 19, 19 and 19, respectively). The data were presented as mean ± SD, # P < 0.05, ## P < 0.01 compared with the Con group.


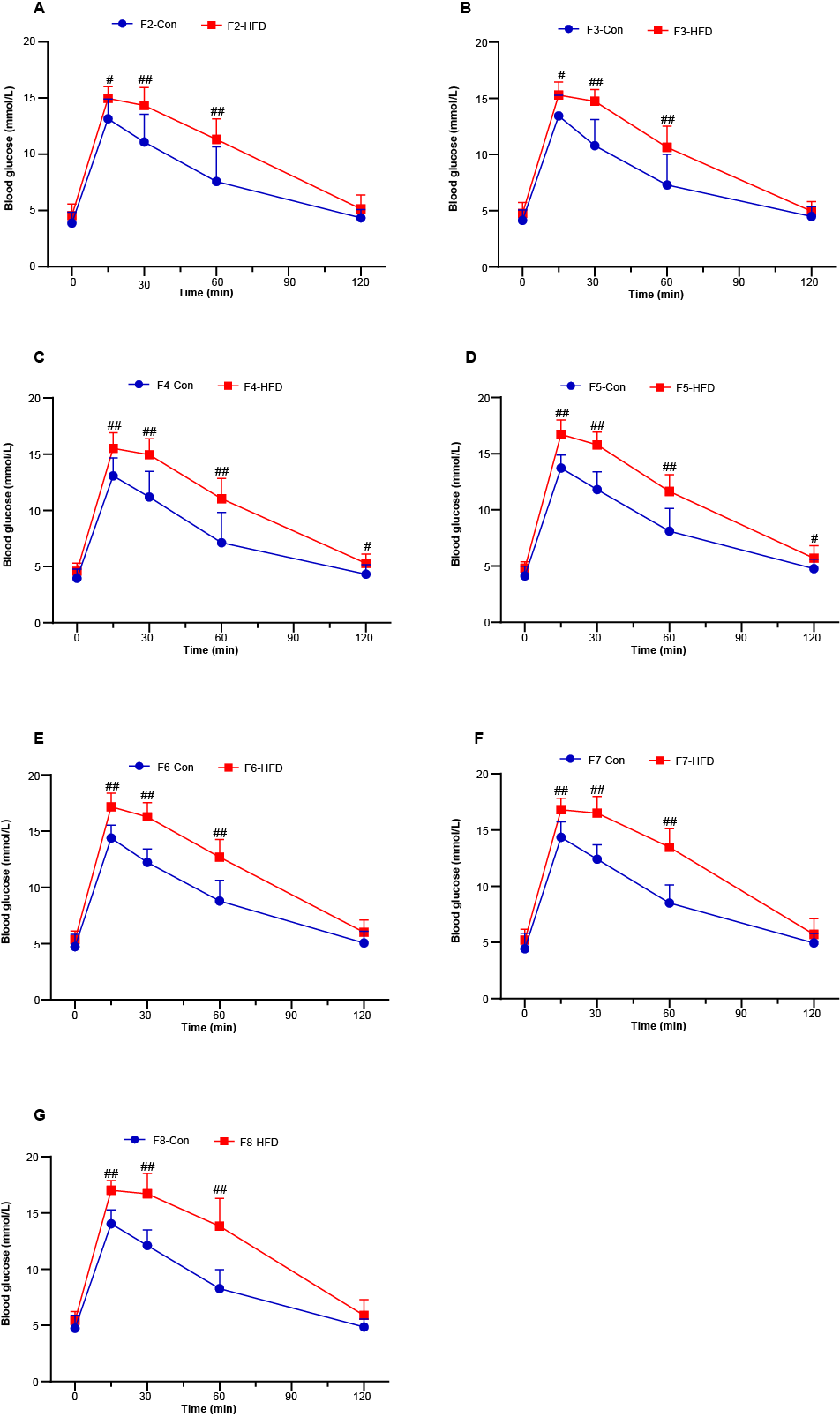


**Supplementary Figure 2.** Paternal sustained multi-generational HFD disrupts glucose tolerance in subsequent generation offspring. The OGTT were performed to evaluate the mice glucose homeostasis. A-G: Blood glucose content during OGTT in F2, F3, F4, F5, F6, F7 and F8 male mice (n = 10). The data were presented as mean ± SD, # P < 0.05, ## P < 0.01 compared with the Con group. OGTT: Oral glucose tolerance test.


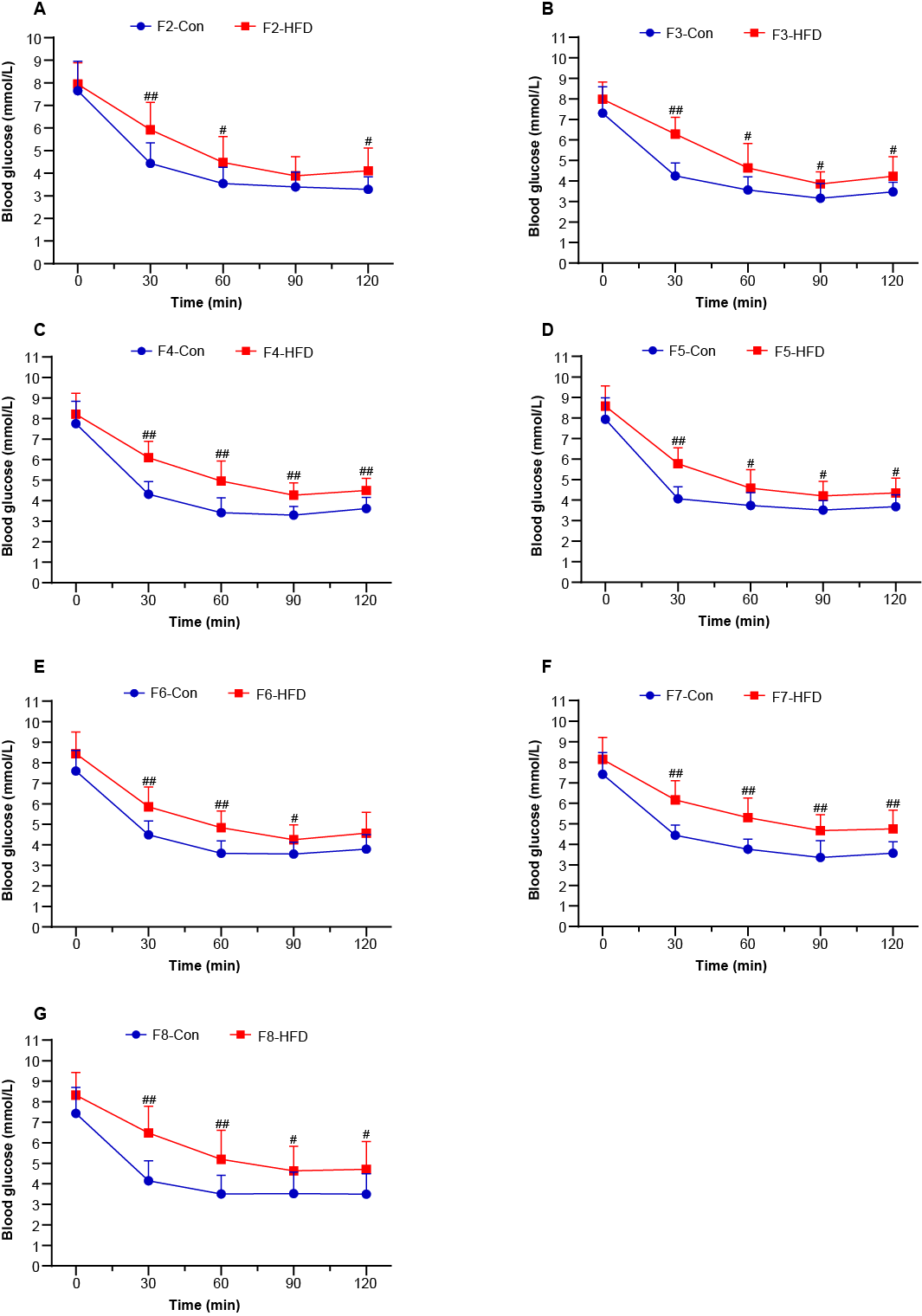


**Supplementary Figure 3.** Paternal sustained multi-generational HFD disrupts insulin sensitivity in subsequent generation offspring. The OITT were performed to evaluate the mice glucose homeostasis. A-G: Blood glucose content during OITT in F2, F3, F4, F5, F5, F6, F7 and F8 male mice (n = 10). The data were presented as mean ± SD, # P < 0.05, ## P < 0.01 compared with the Con group. OITT: Oral insulin tolerance test.


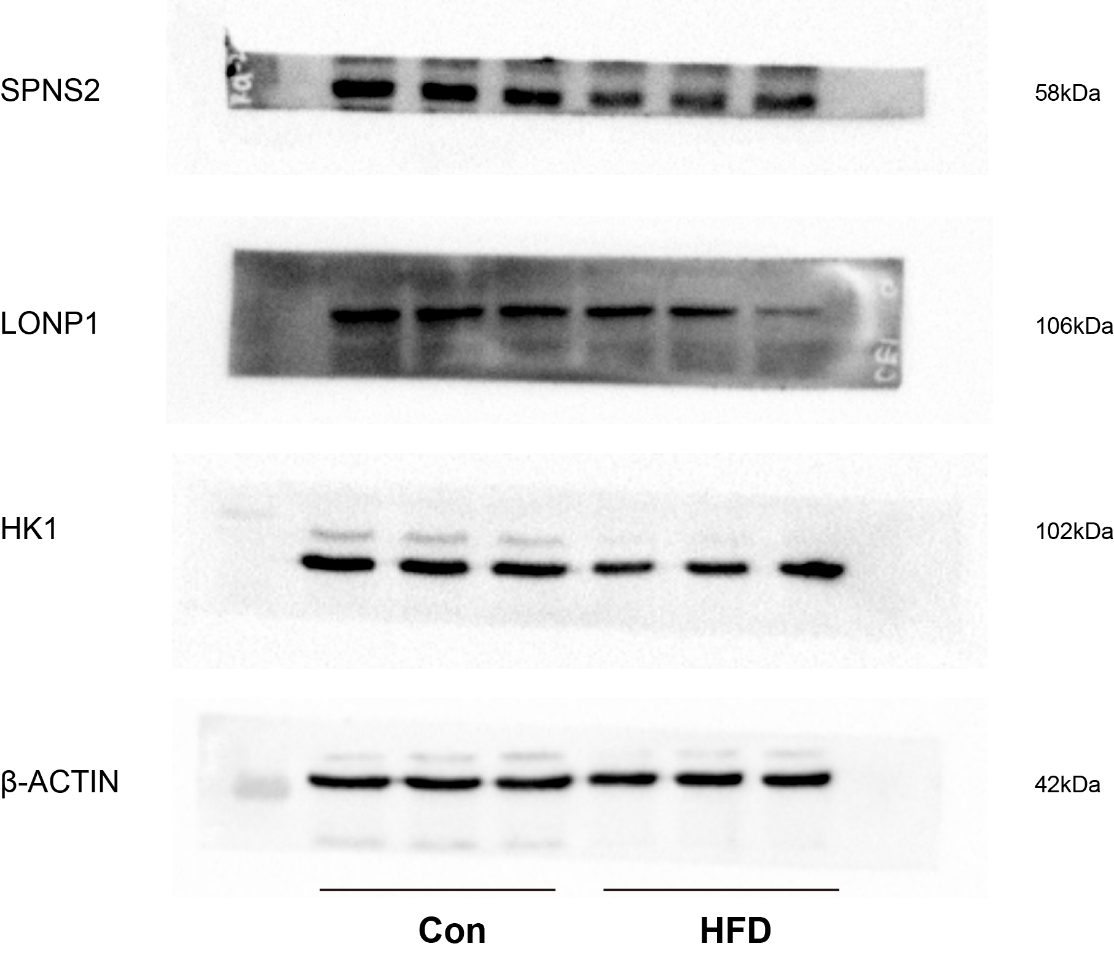


**Supplementary Figure 4.** The original raw image of electrophoretic gels and blots related to the Figure 8.

## Supplementary Tables

Table S1-1 The genes down-regulated expression by HFD

| **Gene**  **Name** | **Fold change (HFD/Con)** | ***p***  **Value** | **Gene**  **Name** | **Fold change (HFD/Con)** | ***p***  **Value** | **Gene**  **Name** | **Fold change (HFD/Con)** | ***p***  **Value** |
| --- | --- | --- | --- | --- | --- | --- | --- | --- |
| *Gm9893* | 0.261 | 0.0471 | *Unc13a* | 0.407 | 0.0003 | *Gabra5* | 0.434 | 0.0310 |
| *Grm2* | 0.283 | 0.0093 | *Lrrc4* | 0.408 | 0.0222 | *Dhx58* | 0.436 | 0.0153 |
| *Sh3bp1* | 0.288 | 0.0002 | *AA415398* | 0.409 | 0.0121 | *Ggt6* | 0.436 | 0.0494 |
| *Ggt5* | 0.299 | 0.0061 | *Sept11* | 0.409 | 0.0260 | *Cox6a2* | 0.436 | 0.0023 |
| *Gm4186* | 0.322 | 0.0486 | *Gm4081* | 0.409 | 0.0283 | *Gm6164* | 0.438 | 0.0060 |
| *Gm12863* | 0.326 | 0.0149 | *6820431F20Rik* | 0.410 | 0.0154 | *A430057M04Rik* | 0.438 | 0.0314 |
| *Mdfi* | 0.334 | 0.0481 | *2900062L11Rik* | 0.410 | 0.0107 | *Slc37a4* | 0.438 | 0.0266 |
| *Cabp2* | 0.337 | 0.0416 | *Flot1* | 0.412 | 0.0420 | *Plin2* | 0.439 | 0.0208 |
| *Cpped1* | 0.343 | 0.0086 | *Chn1* | 0.413 | 0.0298 | *Gm8509* | 0.441 | 0.0073 |
| *Chmp4b* | 0.350 | 0.0010 | *Gm4464* | 0.413 | 0.0264 | *Rpap1* | 0.442 | 0.0005 |
| *Prr3* | 0.362 | 0.0372 | *D730002M21Rik* | 0.415 | 0.0222 | *Pou6f1* | 0.443 | 0.0287 |
| *Magi1* | 0.365 | 0.0247 | *Fcer2a* | 0.415 | 0.0239 | *Rnf146* | 0.444 | 0.0331 |
| *Il31* | 0.367 | 0.0123 | *2510048L02Rik* | 0.416 | 0.0365 | *4930470P17Rik* | 0.444 | 0.0048 |
| *9130227C08Rik* | 0.369 | 0.0407 | *L3mbtl2* | 0.418 | 0.0279 | *Sct* | 0.447 | 0.0360 |
| *Raver1* | 0.372 | 0.0028 | *Ccr3* | 0.418 | 0.0208 | *Igh* | 0.448 | 0.0268 |
| *Dclre1b* | 0.379 | 0.0040 | *Abca7* | 0.420 | 0.0309 | *Lats2* | 0.449 | 0.0032 |
| *Gpr62* | 0.380 | 0.0168 | *Zfp318* | 0.420 | 0.0122 | *Clic4* | 0.449 | 0.0059 |
| *Als2cl* | 0.381 | 0.0387 | *Liph* | 0.421 | 0.0383 | *Ptprd* | 0.450 | 0.0041 |
| *Foxp2* | 0.382 | 0.0323 | *Fiz1* | 0.422 | 0.0142 | *Cobll1* | 0.450 | 0.0096 |
| *Zbtb46* | 0.383 | 0.0205 | *Gm7052* | 0.422 | 0.0189 | *Fam53b* | 0.451 | 0.0277 |
| *Plcxd1* | 0.384 | 0.0125 | *Cdk9* | 0.423 | 0.0039 | *Baz1b* | 0.451 | 0.0085 |
| *D630042F21Rik* | 0.384 | 0.0085 | *Chaf1a* | 0.424 | 0.0065 | *Cabp1* | 0.451 | 0.0155 |
| *Gm10662* | 0.385 | 0.0342 | *Foxo3* | 0.427 | 0.0137 | *Gm8615* | 0.452 | 0.0215 |
| *Tcea2* | 0.385 | 0.0220 | *Ddr1* | 0.427 | 0.0149 | *Senp5* | 0.452 | 0.0108 |
| *6720457D02Rik* | 0.388 | 0.0004 | *Smc1a* | 0.428 | 0.0220 | *Samd10* | 0.453 | 0.0062 |
| *Ntsr2* | 0.388 | 0.0003 | *Zdhhc22* | 0.428 | 0.0031 | *Gm652* | 0.453 | 0.0157 |
| *Vamp2* | 0.388 | 0.0005 | *Flg* | 0.428 | 0.0081 | *Rangrf* | 0.453 | 0.0044 |
| *Chd7* | 0.392 | 0.0193 | *Gnb4* | 0.429 | 0.0312 | *Hsd3b3* | 0.453 | 0.0084 |
| *Rasgrp2* | 0.394 | 0.0220 | *Gm5813* | 0.430 | 0.0210 | *Nuak2* | 0.453 | 0.0210 |
| *2410018L13Rik* | 0.397 | 0.0182 | *Grpel2* | 0.430 | 0.0209 | *Zmynd8* | 0.454 | 0.0150 |
| *Gm10590* | 0.403 | 0.0438 | *A430105I19Rik* | 0.431 | 0.0002 | *Gm2541* | 0.454 | 0.0488 |
| *4930590A17Rik* | 0.406 | 0.0245 | *664858* | 0.433 | 0.0056 | *Ccl27a* | 0.454 | 0.0328 |
| *Mxd1* | 0.406 | 0.0095 | *Sall3* | 0.433 | 0.0431 | *Cd300lg* | 0.455 | 0.0055 |

Table S1-1 (continued)

| **Gene**  **Name** | **Fold change (HFD/Con)** | ***p***  **Value** | **Gene**  **Name** | **Fold change (HFD/Con)** | ***p***  **Value** | **Gene**  **Name** | **Fold change (HFD/Con)** | ***p***  **Value** |
| --- | --- | --- | --- | --- | --- | --- | --- | --- |
| *Rasa3* | 0.455 | 0.0344 | *Gm4006* | 0.465 | 0.0057 | *Vax2os1* | 0.479 | 0.0459 |
| *Ambn* | 0.455 | 0.0388 | *Gm5895* | 0.465 | 0.0397 | *2610307P16Rik* | 0.479 | 0.0244 |
| *Inpp5j* | 0.456 | 0.0221 | *4930503B20Rik* | 0.465 | 0.0313 | *Gm3627* | 0.480 | 0.0377 |
| *Ctrl* | 0.456 | 0.0184 | *Col16a1* | 0.466 | 0.0074 | *Gm2981* | 0.480 | 0.0150 |
| *Gm8519* | 0.456 | 0.0317 | *Slc38a6* | 0.466 | 0.0001 | *Unc93b1* | 0.480 | 0.0459 |
| *Fam180a* | 0.457 | 0.0277 | *Gm12134* | 0.466 | 0.0163 | *S100a14* | 0.480 | 0.0116 |
| *Sat2* | 0.458 | 0.0067 | *Zfp454* | 0.466 | 0.0320 | *Vmn2r60* | 0.480 | 0.0006 |
| *2810047C21Rik1* | 0.458 | 0.0351 | *Irf3* | 0.467 | 0.0222 | *Ccdc71* | 0.481 | 0.0493 |
| *Rhox12* | 0.458 | 0.0383 | *Adi1* | 0.467 | 0.0236 | *Car14* | 0.481 | 0.0407 |
| *Vmn2r-ps26* | 0.458 | 0.0299 | *Csprs* | 0.467 | 0.0294 | *EG627782* | 0.482 | 0.0441 |
| *Pilra* | 0.459 | 0.0233 | *Stip1* | 0.467 | 0.0143 | *Olfr509* | 0.482 | 0.0232 |
| *Gm13546* | 0.459 | 0.0107 | *Phkb* | 0.467 | 0.0049 | *Neto1* | 0.482 | 0.0439 |
| *Veph1* | 0.459 | 0.0153 | *Haghl* | 0.467 | 0.0037 | *Flnb* | 0.482 | 0.0026 |
| *Olfr948* | 0.459 | 0.0142 | *1700109F18Rik* | 0.468 | 0.0009 | *Gm2661* | 0.482 | 0.0454 |
| *Khdrbs1* | 0.460 | 0.0059 | *Eif4g1* | 0.468 | 0.0003 | *Gm8149* | 0.483 | 0.0094 |
| *Pdx1* | 0.460 | 0.0053 | *Chst7* | 0.469 | 0.0119 | *Cyp3a16* | 0.483 | 0.0131 |
| *Gm3693* | 0.460 | 0.0255 | *Rab11fip3* | 0.469 | 0.0335 | *Psma3* | 0.483 | 0.0193 |
| *Gm15417* | 0.461 | 0.0146 | *Pisd-ps3* | 0.469 | 0.0105 | *Galntl1* | 0.483 | 0.0439 |
| *Atp1a2* | 0.461 | 0.0287 | *Pld5* | 0.470 | 0.0454 | *Gm14085* | 0.483 | 0.0380 |
| *1700034H15Rik* | 0.461 | 0.0218 | *Gm2259* | 0.470 | 0.0037 | *Shprh* | 0.484 | 0.0158 |
| *Srrm4* | 0.461 | 0.0131 | *Rgs16* | 0.470 | 0.0385 | *Odc1* | 0.485 | 0.0275 |
| *Fam78a* | 0.461 | 0.0393 | *Ctso* | 0.471 | 0.0011 | *Itga7* | 0.486 | 0.0177 |
| *Trim67* | 0.461 | 0.0031 | *Gm6096* | 0.471 | 0.0456 | *Tcf7l2* | 0.486 | 0.0045 |
| *Sncb* | 0.462 | 0.0389 | *Abr* | 0.472 | 0.0248 | *Gm3117* | 0.486 | 0.0121 |
| *Pla2g7* | 0.462 | 0.0174 | *Gm9607* | 0.472 | 0.0382 | *BC054059* | 0.486 | 0.0328 |
| *Apol8* | 0.462 | 0.0045 | *Rhox3h* | 0.472 | 0.0046 | *Cby3* | 0.487 | 0.0122 |
| *4930442L01Rik* | 0.463 | 0.0200 | *Pdgfra* | 0.474 | 0.0335 | *Olfr347* | 0.487 | 0.0200 |
| *Hcst* | 0.463 | 0.0151 | *Cyb561d2* | 0.474 | 0.0365 | *Parp14* | 0.488 | 0.0290 |
| *Elf2* | 0.464 | 0.0435 | *Trit1* | 0.475 | 0.0194 | *BC058420* | 0.488 | 0.0036 |
| *Nfkbid* | 0.464 | 0.0133 | *Tnnt2* | 0.475 | 0.0024 | *Triap1* | 0.488 | 0.0364 |
| *Traf1* | 0.464 | 0.0177 | *Tgm5* | 0.475 | 0.0044 | *6530402F18Rik* | 0.488 | 0.0186 |
| *Mrpl38* | 0.464 | 0.0373 | *Gm5932* | 0.476 | 0.0460 | *Eif4a1* | 0.489 | 0.0310 |
| *Add1* | 0.464 | 0.0220 | *1700065J11Rik* | 0.476 | 0.0334 | *Ccs* | 0.489 | 0.0208 |
| *Vmn2r16* | 0.464 | 0.0157 | *Pms2* | 0.478 | 0.0297 | *Krt34* | 0.489 | 0.0268 |
| *Rad23a* | 0.464 | 0.0104 | *Adamtsl3* | 0.478 | 0.0011 | *Atp6v0a2* | 0.490 | 0.0050 |
| *Sympk* | 0.464 | 0.0111 | *Gm5943* | 0.478 | 0.0144 | *Smug1* | 0.490 | 0.0261 |
| *Phyhd1* | 0.465 | 0.0089 | *Ube2f* | 0.478 | 0.0263 | *Snx1* | 0.490 | 0.0383 |

Table S1-1 (continued)

| **Gene**  **Name** | **Fold change (HFD/Con)** | ***p***  **Value** | **Gene**  **Name** | **Fold change (HFD/Con)** | ***p***  **Value** | **Gene**  **Name** | **Fold change (HFD/Con)** | ***p***  **Value** |
| --- | --- | --- | --- | --- | --- | --- | --- | --- |
| *Kctd1* | 0.491 | 0.0092 | *Lrg1* | 0.503 | 0.0300 | *Ptchd2* | 0.512 | 0.0032 |
| *Gm10113* | 0.491 | 0.0343 | *Slco4c1* | 0.503 | 0.0066 | *Pou3f3* | 0.512 | 0.0217 |
| *Tcfap2b* | 0.491 | 0.0048 | *Zan* | 0.503 | 0.0068 | *Serpinb6a* | 0.512 | 0.0278 |
| *Tgfb1i1* | 0.491 | 0.0271 | *Gm4242* | 0.504 | 0.0188 | *Gm2425* | 0.512 | 0.0252 |
| *Brms1* | 0.493 | 0.0137 | *Rufy1* | 0.504 | 0.0161 | *Rxrg* | 0.513 | 0.0497 |
| *Sycn* | 0.493 | 0.0182 | *Hmgcs2* | 0.504 | 0.0112 | *L3mbtl* | 0.513 | 0.0271 |
| *Gm2282* | 0.493 | 0.0046 | *Cars* | 0.504 | 0.0487 | *Fetub* | 0.514 | 0.0390 |
| *Fmnl3* | 0.493 | 0.0339 | *Oog2* | 0.504 | 0.0384 | *Coro1c* | 0.514 | 0.0138 |
| *Nsmaf* | 0.494 | 0.0219 | *Prss34* | 0.504 | 0.0405 | *Ube2e2* | 0.514 | 0.0106 |
| *AW146154* | 0.494 | 0.0106 | *Zswim7* | 0.504 | 0.0412 | *3110009E18Rik* | 0.515 | 0.0108 |
| *Gm8221* | 0.494 | 0.0089 | *Ablim2* | 0.504 | 0.0330 | *Gm8620* | 0.515 | 0.0293 |
| *AA881470* | 0.495 | 0.0435 | *Ttc16* | 0.505 | 0.0444 | *Btf3l4* | 0.516 | 0.0356 |
| *Pla2g2d* | 0.495 | 0.0036 | *Il22ra2* | 0.505 | 0.0447 | *Gm8764* | 0.516 | 0.0342 |
| *Kpna7* | 0.495 | 0.0346 | *Neo1* | 0.505 | 0.0080 | *Ly6g6c* | 0.516 | 0.0114 |
| *Dbil5* | 0.496 | 0.0149 | *Gm13276* | 0.505 | 0.0007 | *1700011A15Rik* | 0.516 | 0.0003 |
| *Golga1* | 0.496 | 0.0402 | *Shkbp1* | 0.505 | 0.0144 | *Tacc1* | 0.517 | 0.0089 |
| *Prrt4* | 0.497 | 0.0180 | *Uspl1* | 0.506 | 0.0405 | *Atxn10* | 0.517 | 0.0033 |
| *Lhfpl4* | 0.497 | 0.0012 | *Arhgap11a* | 0.506 | 0.0498 | *Sez6l2* | 0.517 | 0.0424 |
| *Plcd3* | 0.497 | 0.0123 | *AI848100* | 0.506 | 0.0130 | *Pcp4l1* | 0.517 | 0.0139 |
| *Unc13d* | 0.497 | 0.0171 | *Il17re* | 0.507 | 0.0159 | *Cbx6-Nptxr* | 0.517 | 0.0479 |
| *1700017B05Rik* | 0.498 | 0.0067 | *Sema5a* | 0.507 | 0.0139 | *Tas2r134* | 0.518 | 0.0354 |
| *Nfatc2* | 0.498 | 0.0045 | *Gm5513* | 0.507 | 0.0000 | *Gm8362* | 0.518 | 0.0200 |
| *Mastl* | 0.498 | 0.0349 | *Oxct2b* | 0.508 | 0.0169 | *Gm3832* | 0.518 | 0.0073 |
| *Fbxo9* | 0.498 | 0.0164 | *Fbxw8* | 0.508 | 0.0313 | *B230344G16Rik* | 0.518 | 0.0373 |
| *E230029C05Rik* | 0.499 | 0.0208 | *Gm2001* | 0.508 | 0.0284 | *Vmn2r25* | 0.518 | 0.0309 |
| *Zfp661* | 0.499 | 0.0383 | *Kcnk2* | 0.509 | 0.0123 | *Ifitm1* | 0.518 | 0.0129 |
| *Fbxo38* | 0.500 | 0.0311 | *Gm6370* | 0.509 | 0.0197 | *Ddx39* | 0.519 | 0.0196 |
| *4933423P22Rik* | 0.500 | 0.0161 | *Gucy2g* | 0.509 | 0.0007 | *Tm7sf2* | 0.520 | 0.0082 |
| *Ppp2r1b* | 0.501 | 0.0027 | *Sytl3* | 0.509 | 0.0303 | *Vsig10l* | 0.520 | 0.0141 |
| *Snx18* | 0.501 | 0.0038 | *Col13a1* | 0.509 | 0.0272 | *Crtc2* | 0.520 | 0.0485 |
| *St8sia1* | 0.502 | 0.0042 | *1700123M08Rik* | 0.510 | 0.0111 | *Nol7* | 0.520 | 0.0363 |
| *Klhl29* | 0.502 | 0.0157 | *Rassf3* | 0.510 | 0.0002 | *Prl2c2* | 0.521 | 0.0230 |
| *D10Bwg1070e* | 0.502 | 0.0069 | *Atp5l* | 0.510 | 0.0325 | *RP23-269N23.3* | 0.521 | 0.0146 |
| *Gp5* | 0.502 | 0.0127 | *Saa2* | 0.510 | 0.0395 | *Gm4053* | 0.522 | 0.0154 |
| *Fndc7* | 0.502 | 0.0023 | *Ubl3* | 0.511 | 0.0422 | *Eya2* | 0.522 | 0.0218 |
| *Agap3* | 0.502 | 0.0116 | *Nr4a3* | 0.511 | 0.0172 | *Tulp2* | 0.523 | 0.0354 |
| *Aoc3* | 0.503 | 0.0022 | *Defb23* | 0.511 | 0.0159 | *Gm3789* | 0.523 | 0.0419 |

Table S1-1 (continued)

| **Gene**  **Name** | **Fold change (HFD/Con)** | ***p***  **Value** | **Gene**  **Name** | **Fold change (HFD/Con)** | ***p***  **Value** | **Gene**  **Name** | **Fold change (HFD/Con)** | ***p***  **Value** |
| --- | --- | --- | --- | --- | --- | --- | --- | --- |
| *Gm2744* | 0.523 | 0.0212 | *Dcpp1* | 0.531 | 0.0130 | *Vmn2r10* | 0.539 | 0.0123 |
| *Nsg1* | 0.523 | 0.0003 | *Trmt2a* | 0.531 | 0.0235 | *4930570N19Rik* | 0.539 | 0.0316 |
| *Ccbp2* | 0.523 | 0.0183 | *Zfp13* | 0.531 | 0.0234 | *Lrrc8d* | 0.539 | 0.0409 |
| *Fbn2* | 0.523 | 0.0222 | *Tcra-V8* | 0.532 | 0.0346 | *5530401A14Rik* | 0.539 | 0.0111 |
| *Usp49* | 0.523 | 0.0188 | *Tmem116* | 0.532 | 0.0152 | *Impdh2* | 0.539 | 0.0066 |
| *Foxp3* | 0.524 | 0.0154 | *Slc26a5* | 0.532 | 0.0007 | *Slc2a9* | 0.539 | 0.0476 |
| *Sart3* | 0.524 | 0.0208 | *Ing4* | 0.532 | 0.0431 | *Ccr6* | 0.539 | 0.0176 |
| *Gm2783* | 0.525 | 0.0234 | *2210411K11Rik* | 0.533 | 0.0204 | *Gm7932* | 0.540 | 0.0007 |
| *Ppid* | 0.526 | 0.0172 | *Art3* | 0.533 | 0.0126 | *Slco1c1* | 0.540 | 0.0086 |
| *Ang* | 0.526 | 0.0260 | *Nup160* | 0.533 | 0.0105 | *Atp13a4* | 0.540 | 0.0064 |
| *Gm13793* | 0.527 | 0.0174 | *Gm9426* | 0.533 | 0.0291 | *Arf1* | 0.540 | 0.0193 |
| *Irx6* | 0.527 | 0.0102 | *Sass6* | 0.533 | 0.0294 | *Mgll* | 0.540 | 0.0394 |
| *9630058J23Rik* | 0.527 | 0.0053 | *Phf23* | 0.533 | 0.0017 | *Hmgb1l* | 0.540 | 0.0007 |
| *Gas2l1* | 0.527 | 0.0000 | *Dnm2* | 0.534 | 0.0124 | *Ccnc* | 0.541 | 0.0440 |
| *Serpinc1* | 0.527 | 0.0461 | *AI197445* | 0.534 | 0.0231 | *Zfp54* | 0.541 | 0.0174 |
| *Mterfd2* | 0.528 | 0.0163 | *Gjc1* | 0.534 | 0.0069 | *AI506816* | 0.541 | 0.0307 |
| *Gpha2* | 0.528 | 0.0124 | *Stk11* | 0.534 | 0.0233 | *Ddx52* | 0.541 | 0.0450 |
| *Crot* | 0.528 | 0.0400 | *Plekha4* | 0.534 | 0.0287 | *Ryr1* | 0.541 | 0.0261 |
| *Vps39* | 0.528 | 0.0087 | *Fbln2* | 0.534 | 0.0036 | *Dda1* | 0.541 | 0.0233 |
| *Ugt3a2* | 0.529 | 0.0391 | *Olr1* | 0.535 | 0.0391 | *1700016J18Rik* | 0.541 | 0.0226 |
| *Gm4516* | 0.529 | 0.0036 | *Ica1* | 0.535 | 0.0468 | *7030402D04Rik* | 0.542 | 0.0062 |
| *Omp* | 0.529 | 0.0217 | *Tpsg1* | 0.535 | 0.0409 | *Actl7b* | 0.542 | 0.0065 |
| *1700012D14Rik* | 0.529 | 0.0484 | *5830417I10Rik* | 0.535 | 0.0169 | *Exoc7* | 0.542 | 0.0389 |
| *B3gnt1* | 0.529 | 0.0298 | *D19Ertd386e* | 0.535 | 0.0473 | *P2rx6* | 0.543 | 0.0305 |
| *Col8a2* | 0.529 | 0.0371 | *Phldb3* | 0.535 | 0.0449 | *4930474N09Rik* | 0.543 | 0.0335 |
| *Gm5580* | 0.529 | 0.0108 | *Pigs* | 0.536 | 0.0321 | *Bsg* | 0.543 | 0.0498 |
| *B020031M17Rik* | 0.529 | 0.0034 | *Spag11a* | 0.537 | 0.0338 | *Gm15340* | 0.543 | 0.0328 |
| *Gm3930* | 0.530 | 0.0310 | *Ppm1k* | 0.537 | 0.0161 | *Mta3* | 0.544 | 0.0292 |
| *Rhod* | 0.530 | 0.0257 | *Lgtn* | 0.537 | 0.0330 | *Tmc7* | 0.544 | 0.0139 |
| *Agbl2* | 0.530 | 0.0063 | *Sfrs8* | 0.537 | 0.0038 | *Alkbh8* | 0.545 | 0.0237 |
| *Gm2814* | 0.530 | 0.0140 | *Amn* | 0.537 | 0.0282 | *Relb* | 0.545 | 0.0012 |
| *Ppp2r5c* | 0.530 | 0.0310 | *Phka2* | 0.537 | 0.0271 | *Icam4* | 0.545 | 0.0430 |
| *Sdk2* | 0.530 | 0.0015 | *Ldb1* | 0.537 | 0.0056 | *Oprm1* | 0.545 | 0.0338 |
| *Sufu* | 0.530 | 0.0042 | *Slc37a1* | 0.537 | 0.0237 | *Ptprcap* | 0.546 | 0.0009 |
| *Jmjd8* | 0.531 | 0.0284 | *2610029G23Rik* | 0.538 | 0.0401 | *Apoe* | 0.546 | 0.0452 |
| *Ran* | 0.531 | 0.0015 | *Gm1008* | 0.538 | 0.0389 | *Gm14475* | 0.546 | 0.0260 |
| Sorbs3 | 0.531 | 0.0048 | *2310046A06Rik* | 0.538 | 0.0108 | *Zbtb7c* | 0.546 | 0.0267 |

Table S1-1 (continued)

| **Gene**  **Name** | **Fold change (HFD/Con)** | ***p***  **Value** | **Gene**  **Name** | **Fold change (HFD/Con)** | ***p***  **Value** | **Gene**  **Name** | **Fold change (HFD/Con)** | ***p***  **Value** |
| --- | --- | --- | --- | --- | --- | --- | --- | --- |
| *Arhgdib* | 0.546 | 0.0417 | *Rhbdf1* | 0.552 | 0.0156 | *Gm2194* | 0.560 | 0.0392 |
| *Stil* | 0.546 | 0.0151 | *Mcm6* | 0.552 | 0.0458 | *Csmd3* | 0.560 | 0.0183 |
| *Gm1564* | 0.546 | 0.0006 | *Smad9* | 0.553 | 0.0199 | *R3hdml* | 0.560 | 0.0177 |
| *Gm3656* | 0.547 | 0.0437 | *Igh-VJ558* | 0.553 | 0.0258 | *Gm3735* | 0.560 | 0.0190 |
| *Skint7* | 0.547 | 0.0282 | *Adamts2* | 0.553 | 0.0384 | *Ccl25* | 0.560 | 0.0457 |
| *Trim25* | 0.547 | 0.0410 | *Galt* | 0.553 | 0.0244 | *Gm14700* | 0.561 | 0.0045 |
| *Caly* | 0.547 | 0.0308 | *Nat6* | 0.553 | 0.0214 | *Ptprm* | 0.561 | 0.0112 |
| *C030046E11Rik* | 0.547 | 0.0453 | *Carkd* | 0.553 | 0.0007 | *Gm4354* | 0.561 | 0.0106 |
| *Rpia* | 0.547 | 0.0177 | *P4htm* | 0.554 | 0.0003 | *Psen2* | 0.561 | 0.0115 |
| *Uap1* | 0.547 | 0.0437 | *Arl5a* | 0.554 | 0.0304 | *Abca8b* | 0.562 | 0.0101 |
| *Gpr65* | 0.548 | 0.0338 | *Kctd19* | 0.554 | 0.0149 | *Rab27a* | 0.562 | 0.0168 |
| *Cmtm2b* | 0.548 | 0.0005 | *Mctp1* | 0.554 | 0.0071 | *Timeless* | 0.562 | 0.0288 |
| *V1rc8* | 0.548 | 0.0294 | *Slc25a41* | 0.554 | 0.0076 | *Dcaf10* | 0.562 | 0.0416 |
| *Sp110* | 0.548 | 0.0346 | *Cnga4* | 0.554 | 0.0091 | *Klk1b11* | 0.562 | 0.0408 |
| *Upf1* | 0.548 | 0.0079 | *Catsper1* | 0.555 | 0.0052 | *Gm9047* | 0.562 | 0.0188 |
| *Gm3079* | 0.548 | 0.0005 | *Guca2b* | 0.555 | 0.0045 | *Aplf* | 0.563 | 0.0450 |
| *Sf1* | 0.549 | 0.0011 | *Rgnef* | 0.555 | 0.0097 | *Stard10* | 0.563 | 0.0111 |
| *Prrg2* | 0.549 | 0.0318 | *Flt3l* | 0.555 | 0.0424 | *Rbm39* | 0.563 | 0.0087 |
| *Zdhhc4* | 0.549 | 0.0460 | *Pex11b* | 0.555 | 0.0033 | *Faah* | 0.563 | 0.0122 |
| *Dsg2* | 0.549 | 0.0022 | *Gm4031* | 0.556 | 0.0085 | *Gm11974* | 0.563 | 0.0119 |
| *Tnip1* | 0.549 | 0.0330 | *Rnf220* | 0.556 | 0.0177 | *Htr7* | 0.563 | 0.0217 |
| *Trak2* | 0.550 | 0.0158 | *Hace1* | 0.556 | 0.0077 | *Fanci* | 0.564 | 0.0330 |
| *Zfp703* | 0.550 | 0.0026 | *Rad51l1* | 0.556 | 0.0397 | *Gm4494* | 0.564 | 0.0002 |
| *Gm3813* | 0.550 | 0.0062 | *Engase* | 0.557 | 0.0406 | *Baz2b* | 0.564 | 0.0166 |
| *Cdkn2d* | 0.550 | 0.0215 | *Olfr389* | 0.557 | 0.0475 | *Kdm5d* | 0.564 | 0.0372 |
| *Dmrtb1* | 0.550 | 0.0439 | *Polr3h* | 0.557 | 0.0422 | *Gm7300* | 0.564 | 0.0450 |
| *Gm5151* | 0.551 | 0.0027 | *Trav2* | 0.558 | 0.0211 | *Gm11556* | 0.564 | 0.0105 |
| *Gm1082* | 0.551 | 0.0462 | *Gm4766* | 0.558 | 0.0120 | *Gsto2* | 0.564 | 0.0099 |
| *Dynlt1* | 0.551 | 0.0127 | *Gsg1l* | 0.558 | 0.0149 | *Cdc42bpg* | 0.564 | 0.0029 |
| *Gm4534* | 0.551 | 0.0150 | *Gm2605* | 0.559 | 0.0358 | *Spred1* | 0.565 | 0.0366 |
| *4930433N12Rik* | 0.551 | 0.0159 | *Ap1b1* | 0.559 | 0.0209 | *Prkcd* | 0.565 | 0.0171 |
| *Rhox3g* | 0.551 | 0.0095 | *Gm6530* | 0.559 | 0.0163 | *Egln3* | 0.565 | 0.0279 |
| *Ubn1* | 0.551 | 0.0441 | *Xlr3a* | 0.560 | 0.0302 | *Ankrd24* | 0.565 | 0.0235 |
| *Tmem26* | 0.551 | 0.0327 | *Gm2451* | 0.560 | 0.0184 | *Kif19a* | 0.565 | 0.0063 |
| *Sync* | 0.552 | 0.0012 | *D630048o14rik* | 0.560 | 0.0250 | *B230120H23Rik* | 0.565 | 0.0203 |
| *9530028C05* | 0.552 | 0.0080 | *Gm3265* | 0.560 | 0.0362 | *Iltifb* | 0.565 | 0.0284 |
| *Kalrn* | 0.552 | 0.0297 | *Krt73* | 0.560 | 0.0419 | *Nhej1* | 0.565 | 0.0447 |

Table S1-1 (continued)

| **Gene**  **Name** | **Fold change (HFD/Con)** | ***p***  **Value** | **Gene**  **Name** | **Fold change (HFD/Con)** | ***p***  **Value** | **Gene**  **Name** | **Fold change (HFD/Con)** | ***p***  **Value** |
| --- | --- | --- | --- | --- | --- | --- | --- | --- |
| *Gm3980* | 0.565 | 0.0286 | *Siglec5* | 0.573 | 0.0105 | *Ppat* | 0.578 | 0.0278 |
| *Txlna* | 0.566 | 0.0066 | *Tceb2* | 0.573 | 0.0045 | *Ube2v1* | 0.578 | 0.0124 |
| *Tifab* | 0.566 | 0.0116 | *Acsm5* | 0.573 | 0.0167 | *Olfr91* | 0.578 | 0.0186 |
| *Mrps31* | 0.566 | 0.0015 | *Dock1* | 0.573 | 0.0289 | *C330023M02Rik* | 0.579 | 0.0455 |
| *Gm2486* | 0.566 | 0.0130 | *Parp1* | 0.573 | 0.0074 | *Bub1* | 0.579 | 0.0245 |
| *Mybbp1a* | 0.566 | 0.0304 | *Dnajb5* | 0.573 | 0.0179 | *Vmn2r-ps130* | 0.579 | 0.0349 |
| *Spats1* | 0.567 | 0.0112 | *Cck* | 0.573 | 0.0096 | *Gm10436* | 0.579 | 0.0341 |
| *Sema6c* | 0.567 | 0.0037 | *Odz3* | 0.573 | 0.0002 | *Obscn* | 0.579 | 0.0103 |
| *Zfp324* | 0.567 | 0.0402 | *Aldob* | 0.573 | 0.0312 | *Pax3* | 0.580 | 0.0256 |
| *Myh6* | 0.567 | 0.0234 | *Foxb1* | 0.573 | 0.0482 | *Tspyl3* | 0.580 | 0.0481 |
| *Zeb2* | 0.567 | 0.0216 | *Wnt2b* | 0.573 | 0.0211 | *9430023L20Rik* | 0.580 | 0.0095 |
| *Tnfrsf14* | 0.567 | 0.0311 | *2600006L11Rik* | 0.573 | 0.0009 | *Ppfibp2* | 0.580 | 0.0043 |
| *Gm13180* | 0.567 | 0.0483 | *1810063B07Rik* | 0.574 | 0.0235 | *Scn2b* | 0.580 | 0.0017 |
| *Tjap1* | 0.568 | 0.0373 | *9430016H08Rik* | 0.574 | 0.0002 | *Gm2240* | 0.581 | 0.0008 |
| *Dlx6os1* | 0.568 | 0.0002 | *Rapgef1* | 0.574 | 0.0185 | *Ctla2a* | 0.581 | 0.0295 |
| *Hmbox1* | 0.568 | 0.0077 | *Gm6033* | 0.574 | 0.0220 | *Socs3* | 0.581 | 0.0489 |
| *3425401B19Rik* | 0.568 | 0.0036 | *C130026I21Rik* | 0.575 | 0.0402 | *1700010H22Rik* | 0.581 | 0.0112 |
| *Tbc1d20* | 0.568 | 0.0424 | *Gm3491* | 0.575 | 0.0438 | *Mvk* | 0.581 | 0.0477 |
| *Olfr691* | 0.568 | 0.0334 | *Ttn* | 0.575 | 0.0238 | *Gfi1* | 0.581 | 0.0192 |
| *Zfp281* | 0.568 | 0.0447 | *Lcat* | 0.575 | 0.0286 | *Slc1a2* | 0.582 | 0.0466 |
| *Tcof1* | 0.569 | 0.0340 | *Anapc5* | 0.575 | 0.0327 | *Plp1* | 0.582 | 0.0087 |
| *Polr3d* | 0.569 | 0.0303 | *Plekhg1* | 0.576 | 0.0271 | *Mef2d* | 0.582 | 0.0186 |
| *Kif2a* | 0.570 | 0.0044 | *Cdc42bpa* | 0.576 | 0.0227 | *2410001C21Rik* | 0.582 | 0.0310 |
| *Gm2258* | 0.570 | 0.0239 | *E430029J22Rik* | 0.576 | 0.0048 | *Gm7339* | 0.582 | 0.0327 |
| *BC031781* | 0.570 | 0.0204 | *Gm4135* | 0.576 | 0.0204 | *Eef1d* | 0.582 | 0.0383 |
| *Ear2* | 0.570 | 0.0017 | *Rcor2* | 0.577 | 0.0240 | *Ubl7* | 0.582 | 0.0334 |
| *Tubg1* | 0.570 | 0.0312 | *Rabl2a* | 0.577 | 0.0290 | *Dnajc12* | 0.582 | 0.0067 |
| *Slc18a3* | 0.570 | 0.0350 | *Zim3* | 0.577 | 0.0229 | *Elmo2* | 0.583 | 0.0254 |
| *Ilkap* | 0.570 | 0.0032 | *BC048609* | 0.577 | 0.0093 | *Pdlim7* | 0.583 | 0.0027 |
| *Ogfod2* | 0.571 | 0.0340 | *Cdh23* | 0.577 | 0.0024 | *Gm8752* | 0.583 | 0.0355 |
| *Gm2184* | 0.571 | 0.0269 | *Pank4* | 0.577 | 0.0237 | *1700001L19Rik* | 0.583 | 0.0084 |
| *1110018G07Rik* | 0.571 | 0.0100 | *Mcoln3* | 0.577 | 0.0005 | *B230110G15Rik* | 0.583 | 0.0069 |
| *Gm9233* | 0.571 | 0.0141 | *Ppp1r7* | 0.577 | 0.0041 | *Vmn2r75* | 0.583 | 0.0049 |
| *Trip11* | 0.572 | 0.0230 | *Cpt1a* | 0.577 | 0.0263 | *Ube2i* | 0.583 | 0.0298 |
| *Csnk1e* | 0.572 | 0.0351 | *Srr* | 0.577 | 0.0098 | *Hba-x* | 0.584 | 0.0250 |
| *Gm13288* | 0.572 | 0.0460 | *4930443B20Rik* | 0.578 | 0.0487 | *Derl2* | 0.584 | 0.0258 |
| *Apoa4* | 0.572 | 0.0153 | *Gm5589* | 0.578 | 0.0217 | *Srbd1* | 0.584 | 0.0463 |

Table S1-1 (continued)

| **Gene**  **Name** | **Fold change (HFD/Con)** | ***p***  **Value** | **Gene**  **Name** | **Fold change (HFD/Con)** | ***p***  **Value** | **Gene**  **Name** | **Fold change (HFD/Con)** | ***p***  **Value** |
| --- | --- | --- | --- | --- | --- | --- | --- | --- |
| *6430571L13Rik* | 0.584 | 0.0485 | *Secisbp2* | 0.588 | 0.0070 | *Vps53* | 0.594 | 0.0067 |
| *Gm3835* | 0.584 | 0.0027 | *Hck* | 0.588 | 0.0023 | *Slc16a14* | 0.594 | 0.0426 |
| *Peg13* | 0.584 | 0.0087 | *Cenpk* | 0.588 | 0.0199 | *Omt2b* | 0.595 | 0.0022 |
| *Il16* | 0.585 | 0.0020 | *Mllt4* | 0.589 | 0.0024 | *5930430L01Rik* | 0.595 | 0.0044 |
| *Vps52* | 0.585 | 0.0436 | *Crkrs* | 0.589 | 0.0229 | *Gm4684* | 0.595 | 0.0001 |
| *Vgf* | 0.585 | 0.0189 | *Oprl1* | 0.589 | 0.0197 | *Itch* | 0.595 | 0.0452 |
| *Amz2* | 0.585 | 0.0125 | *Gm2113* | 0.589 | 0.0019 | *Acin1* | 0.595 | 0.0021 |
| *1810022K09Rik* | 0.585 | 0.0309 | *Brwd3* | 0.589 | 0.0155 | *Tacc2* | 0.595 | 0.0023 |
| *Prok1* | 0.585 | 0.0395 | *4930428E07Rik* | 0.589 | 0.0079 | *Vmn2r-ps122* | 0.595 | 0.0009 |
| *Camkv* | 0.585 | 0.0166 | *Gm4133* | 0.590 | 0.0200 | *Taf15* | 0.595 | 0.0325 |
| *Gm5426* | 0.585 | 0.0042 | *Golgb1* | 0.590 | 0.0231 | *Gm44* | 0.596 | 0.0434 |
| *Gm2470* | 0.585 | 0.0427 | *4930543D07Rik* | 0.590 | 0.0340 | *Lonp1* | 0.596 | 0.0077 |
| *Mybpc3* | 0.586 | 0.0347 | *Btrc* | 0.590 | 0.0232 | *Dgkh* | 0.596 | 0.0281 |
| *2310004N24Rik* | 0.586 | 0.0130 | *Cd200r3* | 0.590 | 0.0174 | *Angel1* | 0.596 | 0.0259 |
| *Brd3* | 0.586 | 0.0366 | *Rpusd3* | 0.590 | 0.0353 | *Myo1e* | 0.596 | 0.0125 |
| *Cd109* | 0.586 | 0.0475 | *Zdhhc19* | 0.590 | 0.0462 | *0610033M10Rik* | 0.596 | 0.0326 |
| *Pitpnm2* | 0.586 | 0.0161 | *Gm1153* | 0.591 | 0.0147 | *Mbd5* | 0.596 | 0.0037 |
| *Gm2154* | 0.586 | 0.0001 | *Tnk1* | 0.591 | 0.0058 | *Gm9636* | 0.597 | 0.0291 |
| *Uck2* | 0.587 | 0.0123 | *Sox10* | 0.591 | 0.0382 | *Thsd1* | 0.597 | 0.0283 |
| *1700092M07Rik* | 0.587 | 0.0002 | *Ptprt* | 0.591 | 0.0054 | *Kri1* | 0.597 | 0.0490 |
| *Il21* | 0.587 | 0.0057 | *Zc3h12a* | 0.591 | 0.0080 | *Sept14* | 0.597 | 0.0043 |
| *Slc4a7* | 0.587 | 0.0211 | *Gm6829* | 0.591 | 0.0082 | *Sema3a* | 0.597 | 0.0228 |
| *Rab11b* | 0.587 | 0.0439 | *1700029P11Rik* | 0.592 | 0.0046 | *Ankle2* | 0.597 | 0.0124 |
| *Rbm42* | 0.587 | 0.0244 | *Sdccag3* | 0.592 | 0.0035 | *6720456H20Rik* | 0.597 | 0.0420 |
| *4930564K09Rik* | 0.587 | 0.0299 | *Nob1* | 0.592 | 0.0390 | *Trak1* | 0.597 | 0.0393 |
| *Prr5* | 0.587 | 0.0364 | *Jup* | 0.592 | 0.0209 | *Gm10052* | 0.597 | 0.0349 |
| *Dbx2* | 0.587 | 0.0006 | *Fam20a* | 0.592 | 0.0112 | *2310037I24Rik* | 0.597 | 0.0068 |
| *Pvalb* | 0.588 | 0.0354 | *Poll* | 0.592 | 0.0330 | *Camsap1* | 0.598 | 0.0138 |
| *Gm3860* | 0.588 | 0.0082 | *Erc1* | 0.593 | 0.0130 | *Tmem107* | 0.598 | 0.0280 |
| *4931419H13Rik* | 0.588 | 0.0493 | *Tgm7* | 0.593 | 0.0436 | *Igh* | 0.598 | 0.0170 |
| *Tmprss3* | 0.588 | 0.0482 | *1110008J03Rik* | 0.593 | 0.0393 | *2900092E17Rik* | 0.598 | 0.0080 |
| *Glra4* | 0.588 | 0.0233 | *Hmbs* | 0.593 | 0.0192 | *Gm10345* | 0.598 | 0.0005 |
| *Tcfeb* | 0.588 | 0.0003 | *4933404I11Rik* | 0.593 | 0.0218 | *Rwdd2a* | 0.598 | 0.0152 |
| *Gm13292* | 0.588 | 0.0118 | *Lpcat3* | 0.593 | 0.0372 | *Src* | 0.598 | 0.0243 |
| *2410024N18Rik* | 0.588 | 0.0280 | *Gm2827* | 0.594 | 0.0421 | *Bcl2l1* | 0.598 | 0.0366 |
| *Bglap-rs1* | 0.588 | 0.0054 | *Bai2* | 0.594 | 0.0026 | *Arl11* | 0.598 | 0.0212 |
| *Olfr1338* | 0.588 | 0.0352 | *Hif1an* | 0.594 | 0.0110 | *Nupl1* | 0.598 | 0.0147 |

Table S1-1 (continued)

| **Gene**  **Name** | **Fold change (HFD/Con)** | ***p***  **Value** | **Gene**  **Name** | **Fold change (HFD/Con)** | ***p***  **Value** | **Gene**  **Name** | **Fold change (HFD/Con)** | ***p***  **Value** |
| --- | --- | --- | --- | --- | --- | --- | --- | --- |
| *Zfp108* | 0.599 | 0.0164 | *Atxn2* | 0.604 | 0.0448 | *Myl3* | 0.607 | 0.0460 |
| *Gm5858* | 0.599 | 0.0047 | *Mbd6* | 0.604 | 0.0435 | *Ppp2r3a* | 0.607 | 0.0349 |
| *Fanca* | 0.599 | 0.0037 | *Olfr1195* | 0.604 | 0.0384 | *4930539E08Rik* | 0.607 | 0.0195 |
| *Gm3952* | 0.599 | 0.0203 | *Bcl2a1b* | 0.604 | 0.0320 | *Pygl* | 0.607 | 0.0292 |
| *Prkag3* | 0.599 | 0.0339 | *Gm2890* | 0.604 | 0.0470 | *Olfr692* | 0.608 | 0.0451 |
| *H2-Q7* | 0.599 | 0.0345 | *Gm2389* | 0.604 | 0.0116 | *1700121N20Rik* | 0.608 | 0.0132 |
| *St6galnac5* | 0.599 | 0.0199 | *Pde4a* | 0.604 | 0.0049 | *Plekhf1* | 0.608 | 0.0455 |
| *Tcte2* | 0.599 | 0.0406 | *2400001E08Rik* | 0.604 | 0.0021 | *5830403F22Rik* | 0.608 | 0.0296 |
| *Ccdc158* | 0.599 | 0.0473 | *Ak2* | 0.604 | 0.0228 | *Itfg2* | 0.608 | 0.0162 |
| *Krtap5-5* | 0.600 | 0.0033 | *Sbf1* | 0.604 | 0.0097 | *0610037M15Rik* | 0.608 | 0.0292 |
| *A930007I19Rik* | 0.600 | 0.0257 | *Gm5590* | 0.604 | 0.0074 | *Slc25a21* | 0.608 | 0.0423 |
| *Gm3532* | 0.600 | 0.0068 | *Slc16a6* | 0.604 | 0.0472 | *LOC100041034* | 0.608 | 0.0014 |
| *Camk1d* | 0.600 | 0.0271 | *Kank3* | 0.604 | 0.0051 | *Zfp493* | 0.608 | 0.0076 |
| *Pramel4* | 0.600 | 0.0136 | *Heg1* | 0.605 | 0.0373 | *Aurkb* | 0.609 | 0.0021 |
| *Ncaph2* | 0.600 | 0.0262 | *Gm9890* | 0.605 | 0.0263 | *Slc25a45* | 0.609 | 0.0024 |
| *4931408A02Rik* | 0.600 | 0.0053 | *Tbx4* | 0.605 | 0.0405 | *Gm5725* | 0.609 | 0.0265 |
| *Gm4626* | 0.600 | 0.0113 | *Gm8442* | 0.605 | 0.0136 | *Gm3682* | 0.609 | 0.0297 |
| *Smarcc1* | 0.600 | 0.0051 | *4921521F21Rik* | 0.605 | 0.0200 | *Il17ra* | 0.609 | 0.0497 |
| *2310007L24Rik* | 0.600 | 0.0187 | *Foxn4* | 0.605 | 0.0130 | *Gm8020* | 0.610 | 0.0017 |
| *Nkd2* | 0.600 | 0.0009 | *Akap8l* | 0.606 | 0.0434 | *Prb1* | 0.610 | 0.0284 |
| *Gm5551* | 0.600 | 0.0314 | *Gm3834* | 0.606 | 0.0177 | *Vmn2r123* | 0.610 | 0.0348 |
| *Ccdc58* | 0.601 | 0.0477 | *Etv1* | 0.606 | 0.0426 | *Gm9500* | 0.610 | 0.0014 |
| *Med9* | 0.601 | 0.0049 | *Ankra2* | 0.606 | 0.0483 | *Lingo1* | 0.610 | 0.0126 |
| *Gpx2* | 0.601 | 0.0039 | *Gm2180* | 0.606 | 0.0195 | *4930402D18Rik* | 0.610 | 0.0380 |
| *Atp9b* | 0.601 | 0.0439 | *Nkx2-2* | 0.606 | 0.0040 | *A030009H04Rik* | 0.610 | 0.0085 |
| *9330210C06* | 0.601 | 0.0423 | *Zcchc14* | 0.606 | 0.0135 | *Atxn2l* | 0.611 | 0.0103 |
| *Amdhd2* | 0.602 | 0.0216 | *Olfr1447* | 0.606 | 0.0295 | *Tmem19* | 0.611 | 0.0038 |
| *LOC100038908* | 0.602 | 0.0024 | *Pitpnm1* | 0.606 | 0.0152 | *Gm13178* | 0.611 | 0.0267 |
| *Psd3* | 0.602 | 0.0247 | *Ssxb1* | 0.606 | 0.0487 | *Rrp7a* | 0.611 | 0.0097 |
| *Ifna2* | 0.602 | 0.0398 | *Fubp1* | 0.606 | 0.0424 | *Gm9647* | 0.611 | 0.0051 |
| *Vmn2r89* | 0.602 | 0.0411 | *Slc44a1* | 0.607 | 0.0329 | *LOC100039801* | 0.611 | 0.0301 |
| *Traf6* | 0.602 | 0.0408 | *Arhgef11* | 0.607 | 0.0319 | *Impdh1* | 0.612 | 0.0413 |
| *Rexo1* | 0.603 | 0.0217 | *Gm15478* | 0.607 | 0.0224 | *Asnsd1* | 0.612 | 0.0388 |
| *Hmgn2l6* | 0.603 | 0.0416 | *Gm8674* | 0.607 | 0.0027 | *Dctd* | 0.612 | 0.0108 |
| *5730457N03Rik* | 0.603 | 0.0294 | *V1rd10* | 0.607 | 0.0145 | *Als2* | 0.612 | 0.0343 |
| *Alpk2* | 0.603 | 0.0295 | *Prkrip1* | 0.607 | 0.0400 | *Vrk1* | 0.612 | 0.0178 |
| *Atp6v1g3* | 0.604 | 0.0265 | *Rnf219* | 0.607 | 0.0271 | *1700030H01Rik* | 0.612 | 0.0353 |

Table S1-1 (continued)

| **Gene**  **Name** | **Fold change (HFD/Con)** | ***p***  **Value** | **Gene**  **Name** | **Fold change (HFD/Con)** | ***p***  **Value** | **Gene**  **Name** | **Fold change (HFD/Con)** | ***p***  **Value** |
| --- | --- | --- | --- | --- | --- | --- | --- | --- |
| *Ip6k1* | 0.612 | 0.0287 | *1700025F22Rik* | 0.617 | 0.0039 | *Sfrs4* | 0.620 | 0.0362 |
| *Slc36a2* | 0.612 | 0.0202 | *Mapre2* | 0.617 | 0.0217 | *Gm10787* | 0.620 | 0.0098 |
| *Krt2* | 0.612 | 0.0115 | *Lingo4* | 0.617 | 0.0114 | *Gm4703* | 0.621 | 0.0057 |
| *Dcaf17* | 0.612 | 0.0250 | *Kcnj12* | 0.617 | 0.0094 | *Psmb7* | 0.621 | 0.0266 |
| *Sprr2a3* | 0.613 | 0.0237 | *Bbs1* | 0.617 | 0.0299 | *Nphp3* | 0.621 | 0.0077 |
| *Grina* | 0.613 | 0.0139 | *0610040F04Rik* | 0.617 | 0.0256 | *Ccdc76* | 0.621 | 0.0065 |
| *Rcor3* | 0.613 | 0.0186 | *Pla2g3* | 0.618 | 0.0365 | *Gm2478* | 0.621 | 0.0387 |
| *Fhl4* | 0.613 | 0.0174 | *Ankrd39* | 0.618 | 0.0264 | *Hnrnpk* | 0.621 | 0.0308 |
| *C130050O18Rik* | 0.613 | 0.0009 | *Olfr473* | 0.618 | 0.0005 | *Fam154b* | 0.621 | 0.0438 |
| *Nat2* | 0.613 | 0.0089 | *Tnrc6a* | 0.618 | 0.0420 | *Msmp* | 0.621 | 0.0112 |
| *Hnrnph1* | 0.613 | 0.0283 | *Cast* | 0.618 | 0.0294 | *H1foo* | 0.621 | 0.0173 |
| *A130082M07Rik* | 0.613 | 0.0400 | *1700011J10Rik* | 0.618 | 0.0383 | *Chd5* | 0.621 | 0.0016 |
| *EG665203* | 0.613 | 0.0119 | *Cbr2* | 0.619 | 0.0032 | *Phf15* | 0.621 | 0.0186 |
| *Sfi1* | 0.614 | 0.0302 | *Tyms* | 0.619 | 0.0149 | *Gm3228* | 0.621 | 0.0332 |
| *2510009E07Rik* | 0.614 | 0.0092 | *Snx15* | 0.619 | 0.0060 | *Ly6h* | 0.622 | 0.0001 |
| *Olfr919* | 0.614 | 0.0352 | *Krtap4-16* | 0.619 | 0.0353 | *Narg2* | 0.622 | 0.0407 |
| *Eif4ebp2* | 0.614 | 0.0008 | *Phf2* | 0.619 | 0.0482 | *Gm9903* | 0.622 | 0.0153 |
| *Gm4814* | 0.614 | 0.0468 | *Rcl1* | 0.619 | 0.0410 | *4933407C03Rik* | 0.622 | 0.0353 |
| *Gab2* | 0.614 | 0.0257 | *Gm9052* | 0.619 | 0.0328 | *Sh3rf3* | 0.622 | 0.0118 |
| *Zscan4d* | 0.614 | 0.0363 | *1700021K19Rik* | 0.619 | 0.0030 | *Adarb1* | 0.622 | 0.0249 |
| *1700018A14Rik* | 0.615 | 0.0349 | *Echdc1* | 0.619 | 0.0488 | *Sh3glb2* | 0.622 | 0.0386 |
| *Usp48* | 0.615 | 0.0479 | *Utp23* | 0.619 | 0.0378 | *Gja5* | 0.622 | 0.0018 |
| *Dydc1* | 0.615 | 0.0029 | *Cap1* | 0.619 | 0.0281 | *Erc2* | 0.622 | 0.0053 |
| *Pparg* | 0.615 | 0.0411 | *Klra7* | 0.619 | 0.0122 | *Atp9a* | 0.623 | 0.0066 |
| *Piwil4* | 0.615 | 0.0030 | *Wdr55* | 0.619 | 0.0490 | *Gm7149* | 0.623 | 0.0110 |
| *Acot8* | 0.615 | 0.0205 | *1700012P22Rik* | 0.619 | 0.0004 | *Jhdm1d* | 0.623 | 0.0309 |
| *Slc44a2* | 0.616 | 0.0433 | *Tmem206* | 0.619 | 0.0161 | *Olfml2b* | 0.623 | 0.0109 |
| *Vmn2r38* | 0.616 | 0.0374 | *Sdhc* | 0.620 | 0.0317 | *Pkd1l1* | 0.623 | 0.0129 |
| *Pklr* | 0.616 | 0.0014 | *Nupl2* | 0.620 | 0.0212 | *Gm3839* | 0.624 | 0.0031 |
| *Eif2a* | 0.616 | 0.0328 | *Otud5* | 0.620 | 0.0238 | *Vbp1* | 0.624 | 0.0016 |
| *Dsc3* | 0.616 | 0.0258 | *Rnf34* | 0.620 | 0.0125 | *1200014M14Rik* | 0.624 | 0.0082 |
| *Gm3129* | 0.616 | 0.0324 | *Clcn6* | 0.620 | 0.0068 | *4930502E18Rik* | 0.624 | 0.0205 |
| *Gm3049* | 0.617 | 0.0355 | *Srrt* | 0.620 | 0.0173 | *Exoc3l* | 0.624 | 0.0145 |
| *Kcnb1* | 0.617 | 0.0076 | *Insig2* | 0.620 | 0.0105 | *Afmid* | 0.624 | 0.0241 |
| *Zfp710* | 0.617 | 0.0101 | *Lrrtm4* | 0.620 | 0.0058 | *Tmem120b* | 0.624 | 0.0157 |
| *Skiv2l2* | 0.617 | 0.0396 | *Rap1gds1* | 0.620 | 0.0222 | *Sez6* | 0.625 | 0.0019 |
| *Cnn3* | 0.617 | 0.0038 | *Cacnb3* | 0.620 | 0.0210 | *Kcnip1* | 0.625 | 0.0180 |

Table S1-1 (continued)

| **Gene**  **Name** | **Fold change (HFD/Con)** | ***p***  **Value** | **Gene**  **Name** | **Fold change (HFD/Con)** | ***p***  **Value** | **Gene**  **Name** | **Fold change (HFD/Con)** | ***p***  **Value** |
| --- | --- | --- | --- | --- | --- | --- | --- | --- |
| *Lmcd1* | 0.625 | 0.0312 | *Pigw* | 0.629 | 0.0078 | *Gapdh* | 0.633 | 0.0126 |
| *Gm2848* | 0.625 | 0.0174 | *4933400C05Rik* | 0.629 | 0.0345 | *Mc3r* | 0.633 | 0.0003 |
| *Cpxm1* | 0.625 | 0.0208 | *2810006K23Rik* | 0.629 | 0.0127 | *Ikbkb* | 0.633 | 0.0493 |
| *Ubl4b* | 0.625 | 0.0127 | *1700024P12Rik* | 0.629 | 0.0343 | *Hmgn2* | 0.633 | 0.0038 |
| *Nppa* | 0.625 | 0.0240 | *Lrpprc* | 0.629 | 0.0321 | *Trp63* | 0.633 | 0.0048 |
| *Dhrs4* | 0.625 | 0.0394 | *Angptl3* | 0.629 | 0.0150 | *Tcap* | 0.633 | 0.0136 |
| *Schip1* | 0.625 | 0.0440 | *Kcnma1* | 0.629 | 0.0037 | *Cblb* | 0.633 | 0.0414 |
| *Gm4535* | 0.625 | 0.0156 | *Bmp7* | 0.629 | 0.0139 | *Stard9* | 0.633 | 0.0002 |
| *Hk1* | 0.625 | 0.0448 | *Olfr1324* | 0.629 | 0.0235 | *Slc2a4* | 0.633 | 0.0321 |
| *Scarf2* | 0.626 | 0.0268 | *Ube1l* | 0.629 | 0.0160 | *4930581F22Rik* | 0.633 | 0.0045 |
| *4930432K21Rik* | 0.626 | 0.0305 | *Slc25a22* | 0.629 | 0.0147 | *Slc26a6* | 0.633 | 0.0466 |
| *Usp39* | 0.626 | 0.0355 | *2310067B10Rik* | 0.629 | 0.0318 | *Rhoq* | 0.634 | 0.0117 |
| *Tmem129* | 0.626 | 0.0287 | *Zfp827* | 0.629 | 0.0457 | *Crat* | 0.634 | 0.0323 |
| *Gm10168* | 0.626 | 0.0015 | *Kbtbd3* | 0.629 | 0.0329 | *Mrps35* | 0.634 | 0.0126 |
| *Nrg4* | 0.626 | 0.0358 | *Pak4* | 0.630 | 0.0022 | *Atp8a1* | 0.634 | 0.0194 |
| *Igh* | 0.626 | 0.0266 | *Mgam* | 0.630 | 0.0314 | *BC033916* | 0.634 | 0.0359 |
| *Spon1* | 0.626 | 0.0109 | *Itga2b* | 0.630 | 0.0367 | *Gm4144* | 0.634 | 0.0008 |
| *Slc29a2* | 0.626 | 0.0451 | *Setd6* | 0.630 | 0.0477 | *Olfr71* | 0.634 | 0.0210 |
| *Tal1* | 0.626 | 0.0496 | *Gm3721* | 0.630 | 0.0114 | *Cc2d2a* | 0.634 | 0.0201 |
| *Supv3l1* | 0.626 | 0.0304 | *Tatdn3* | 0.630 | 0.0036 | *Gm7904* | 0.634 | 0.0167 |
| *Slc2a3* | 0.627 | 0.0494 | *A530046M15* | 0.631 | 0.0397 | *Atl3* | 0.634 | 0.0350 |
| *Trim55* | 0.627 | 0.0096 | *Sdr39u1* | 0.631 | 0.0087 | *Mtcp1* | 0.634 | 0.0430 |
| *1700040F15Rik* | 0.627 | 0.0409 | *Gm1043* | 0.631 | 0.0280 | *9630028I04Rik* | 0.635 | 0.0153 |
| *Stxbp5l* | 0.627 | 0.0073 | *Magea8* | 0.631 | 0.0429 | *Cyp4f17* | 0.635 | 0.0350 |
| *Orly* | 0.627 | 0.0354 | *Eps8l2* | 0.631 | 0.0229 | *BC024139* | 0.635 | 0.0108 |
| *Cacna2d2* | 0.627 | 0.0064 | *1700024G13Rik* | 0.631 | 0.0419 | *5330416C01Rik* | 0.635 | 0.0232 |
| *Kdelc2* | 0.627 | 0.0472 | *Bmper* | 0.631 | 0.0198 | *Fcf1* | 0.635 | 0.0356 |
| *Zfp605* | 0.627 | 0.0075 | *Neb* | 0.631 | 0.0435 | *Irs3* | 0.635 | 0.0474 |
| *Pcdh10* | 0.627 | 0.0222 | *1190007F08Rik* | 0.631 | 0.0015 | *Ear12* | 0.636 | 0.0004 |
| *Ccnf* | 0.628 | 0.0268 | *A230103J11Rik* | 0.631 | 0.0166 | *Ankrd13b* | 0.636 | 0.0325 |
| *Gm8544* | 0.628 | 0.0035 | *Per3* | 0.631 | 0.0101 | *Kctd3* | 0.636 | 0.0241 |
| *Lingo2* | 0.628 | 0.0088 | *Ypel4* | 0.632 | 0.0466 | *A930013B10Rik* | 0.636 | 0.0186 |
| *Gm2085* | 0.628 | 0.0042 | *Nudt18* | 0.632 | 0.0257 | *Zfp3* | 0.636 | 0.0203 |
| *Dnahc7a* | 0.628 | 0.0348 | *Gm3924* | 0.632 | 0.0117 | *Gm3346* | 0.637 | 0.0181 |
| *Ccdc33* | 0.628 | 0.0143 | *Ppfia3* | 0.632 | 0.0461 | *Arpp19* | 0.637 | 0.0008 |
| *Wbscr28* | 0.628 | 0.0022 | *Gm12302* | 0.632 | 0.0033 | *Olfr464* | 0.637 | 0.0441 |
| *3110062M04Rik* | 0.628 | 0.0071 | *Hdgfrp3* | 0.632 | 0.0321 | *Ncrna00085* | 0.637 | 0.0132 |

Table S1-1 (continued)

| **Gene**  **Name** | **Fold change (HFD/Con)** | ***p***  **Value** | **Gene**  **Name** | **Fold change (HFD/Con)** | ***p***  **Value** | **Gene**  **Name** | **Fold change (HFD/Con)** | ***p***  **Value** |
| --- | --- | --- | --- | --- | --- | --- | --- | --- |
| *Olfr386* | 0.637 | 0.0039 | *Gm2011* | 0.641 | 0.0453 | *Hif3a* | 0.644 | 0.0016 |
| *Hmx2* | 0.637 | 0.0131 | *Gm10888* | 0.641 | 0.0180 | *Fam12* | 0.644 | 0.0053 |
| *Kif2c* | 0.637 | 0.0296 | *Spg11* | 0.641 | 0.0170 | *Cdh11* | 0.644 | 0.0020 |
| *Ereg* | 0.637 | 0.0438 | *Cacna1s* | 0.641 | 0.0245 | *Fam59a* | 0.644 | 0.0383 |
| *D10Ertd610e* | 0.637 | 0.0401 | *Pyroxd2* | 0.641 | 0.0195 | *Pcdh11x* | 0.644 | 0.0029 |
| *Tatdn2* | 0.637 | 0.0450 | *Plekhn1* | 0.641 | 0.0195 | *Ly6e* | 0.644 | 0.0395 |
| *Cyp4f14* | 0.637 | 0.0432 | *2210406O10Rik* | 0.641 | 0.0449 | *Gm1865* | 0.644 | 0.0325 |
| *Brd9* | 0.637 | 0.0255 | *Rax* | 0.641 | 0.0497 | *C1rb* | 0.644 | 0.0270 |
| *Stap1* | 0.638 | 0.0277 | *Sf3b3* | 0.641 | 0.0206 | *Mepce* | 0.644 | 0.0447 |
| *Vill* | 0.638 | 0.0317 | *Tubd1* | 0.641 | 0.0175 | *Gm15411* | 0.644 | 0.0360 |
| *Actr5* | 0.638 | 0.0212 | *Ush2a* | 0.641 | 0.0218 | *Dcaf8* | 0.644 | 0.0231 |
| *Clk3* | 0.638 | 0.0135 | *D730045B01Rik* | 0.641 | 0.0393 | *Plin1* | 0.644 | 0.0367 |
| *Gm13496* | 0.638 | 0.0235 | *4930511I11Rik* | 0.642 | 0.0367 | *Gdf7* | 0.644 | 0.0209 |
| *Pabpc4* | 0.638 | 0.0326 | *Has1* | 0.642 | 0.0116 | *Rsad2* | 0.645 | 0.0023 |
| *P2ry2* | 0.638 | 0.0206 | *Gm4638* | 0.642 | 0.0224 | *Rfx5* | 0.645 | 0.0499 |
| *Gm2397* | 0.638 | 0.0295 | *Kcne1* | 0.642 | 0.0081 | *Dock10* | 0.645 | 0.0371 |
| *Lass3* | 0.638 | 0.0454 | *Lrrc46* | 0.642 | 0.0140 | *Klf13* | 0.645 | 0.0450 |
| *Vmn2r63* | 0.639 | 0.0208 | *Fbxw2* | 0.642 | 0.0250 | *Nhlh1* | 0.645 | 0.0388 |
| *Csrnp3* | 0.639 | 0.0473 | *Retnlg* | 0.642 | 0.0429 | *Dip2b* | 0.645 | 0.0456 |
| *V1rb7* | 0.639 | 0.0244 | *Cul7* | 0.642 | 0.0402 | *Gm8951* | 0.645 | 0.0354 |
| *Nmi* | 0.639 | 0.0110 | *Nlgn2* | 0.642 | 0.0122 | *Gm3241* | 0.645 | 0.0073 |
| *Nol9* | 0.639 | 0.0309 | *Banp* | 0.642 | 0.0218 | *1700030N03Rik* | 0.646 | 0.0224 |
| *Dpf2* | 0.639 | 0.0263 | *Muc19* | 0.642 | 0.0080 | *Gba* | 0.646 | 0.0037 |
| *Ldlr* | 0.639 | 0.0453 | *Prima1* | 0.642 | 0.0222 | *Ltbp4* | 0.646 | 0.0367 |
| *1110037F02Rik* | 0.639 | 0.0089 | *Gm5593* | 0.643 | 0.0229 | *Gm4671* | 0.646 | 0.0499 |
| *4930405J17Rik* | 0.639 | 0.0172 | *Amy2b* | 0.643 | 0.0195 | *Ecd* | 0.646 | 0.0170 |
| *Lmtk3* | 0.640 | 0.0271 | *Gm3546* | 0.643 | 0.0355 | *Ankrd52* | 0.646 | 0.0412 |
| *Sobp* | 0.640 | 0.0219 | *Blm* | 0.643 | 0.0368 | *Ppap2c* | 0.646 | 0.0276 |
| *Mirg* | 0.640 | 0.0012 | *Upf3a* | 0.643 | 0.0032 | *Icosl* | 0.646 | 0.0216 |
| *2810002N01Rik* | 0.640 | 0.0066 | *Olah* | 0.643 | 0.0029 | *Cic* | 0.646 | 0.0130 |
| *4930404I05Rik* | 0.640 | 0.0367 | *Gbf1* | 0.643 | 0.0335 | *Nf2* | 0.647 | 0.0009 |
| *Cnrip1* | 0.640 | 0.0334 | *Wfdc13* | 0.643 | 0.0463 | *Gm6117* | 0.647 | 0.0477 |
| *Pbp2* | 0.640 | 0.0487 | *Gm6237* | 0.643 | 0.0106 | *Btbd16* | 0.647 | 0.0404 |
| *Rwdd3* | 0.640 | 0.0172 | *Nolc1* | 0.643 | 0.0230 | *Epha1* | 0.647 | 0.0035 |
| *Tmem150c* | 0.641 | 0.0076 | *Pnliprp2* | 0.643 | 0.0219 | *Plp2* | 0.648 | 0.0297 |
| *Rhbdd2* | 0.641 | 0.0005 | *Cln3* | 0.644 | 0.0171 | *Eaf1* | 0.648 | 0.0329 |
| *Bcs1l* | 0.641 | 0.0442 | *Tnrc18* | 0.644 | 0.0205 | *2410012M07Rik* | 0.648 | 0.0046 |

Table S1-1 (continued)

| **Gene**  **Name** | **Fold change (HFD/Con)** | ***p***  **Value** | **Gene**  **Name** | **Fold change (HFD/Con)** | ***p***  **Value** | **Gene**  **Name** | **Fold change (HFD/Con)** | ***p***  **Value** |
| --- | --- | --- | --- | --- | --- | --- | --- | --- |
| *Fam54b* | 0.648 | 0.0145 | *Gm2173* | 0.652 | 0.0254 | *Lphn3* | 0.655 | 0.0493 |
| *Ccpg1* | 0.648 | 0.0188 | *Mios* | 0.652 | 0.0160 | *Mphosph9* | 0.655 | 0.0198 |
| *Emilin2* | 0.648 | 0.0147 | *Slc29a1* | 0.652 | 0.0263 | *Egfbp2* | 0.656 | 0.0306 |
| *Gng3* | 0.648 | 0.0141 | *Gm8571* | 0.652 | 0.0059 | *Prr18* | 0.656 | 0.0212 |
| *Psmd8* | 0.648 | 0.0312 | *Rab3il1* | 0.652 | 0.0399 | *Oas1d* | 0.656 | 0.0254 |
| *Cmtm7* | 0.648 | 0.0066 | *Hrh3* | 0.652 | 0.0318 | *Prei4* | 0.656 | 0.0252 |
| *Pigb* | 0.649 | 0.0383 | *Sarm1* | 0.652 | 0.0059 | *Gm1998* | 0.656 | 0.0311 |
| *Gm3514* | 0.649 | 0.0237 | *Gimap4* | 0.652 | 0.0268 | *Pak6* | 0.656 | 0.0424 |
| *Krtap10-4* | 0.649 | 0.0299 | *Hip1r* | 0.652 | 0.0019 | *Sprr2e* | 0.656 | 0.0416 |
| *Onecut1* | 0.649 | 0.0239 | *Mtmr4* | 0.652 | 0.0081 | *D730005E14Rik* | 0.656 | 0.0200 |
| *Sec23a* | 0.649 | 0.0174 | *Rhox9* | 0.653 | 0.0174 | *Zfp191* | 0.656 | 0.0324 |
| *Ednrb* | 0.649 | 0.0437 | *Mare* | 0.653 | 0.0398 | *Gm5103* | 0.657 | 0.0466 |
| *Tshz2* | 0.649 | 0.0021 | *Dennd2c* | 0.653 | 0.0480 | *Gp49a* | 0.657 | 0.0240 |
| *Eif1b* | 0.649 | 0.0288 | *Ubxn11* | 0.653 | 0.0406 | *Smyd1* | 0.657 | 0.0092 |
| *Tex16* | 0.649 | 0.0122 | *Taf1* | 0.653 | 0.0200 | *Xrcc6bp1* | 0.657 | 0.0195 |
| *Rnf130* | 0.649 | 0.0061 | *Ear5* | 0.653 | 0.0282 | *C85492* | 0.657 | 0.0131 |
| *Zfp438* | 0.649 | 0.0480 | *Gm8647* | 0.653 | 0.0003 | *Ppp4r1l* | 0.657 | 0.0500 |
| *Itih4* | 0.649 | 0.0098 | *Slc33a1* | 0.653 | 0.0305 | *Gmppa* | 0.657 | 0.0045 |
| *Ncam1* | 0.649 | 0.0239 | *Vrk3* | 0.653 | 0.0392 | *Vpreb1* | 0.657 | 0.0432 |
| *Pkmyt1* | 0.649 | 0.0124 | *Gm2084* | 0.653 | 0.0368 | *Gm5934* | 0.657 | 0.0337 |
| *Gm7383* | 0.650 | 0.0019 | *4930473A02Rik* | 0.653 | 0.0291 | *Duox1* | 0.657 | 0.0497 |
| *Rbm11* | 0.650 | 0.0190 | *Ywhae* | 0.653 | 0.0364 | *Vars* | 0.657 | 0.0306 |
| *D830044I16Rik* | 0.650 | 0.0497 | *Lrrn4cl* | 0.653 | 0.0008 | *P2rx2* | 0.657 | 0.0374 |
| *Gm4449* | 0.650 | 0.0448 | *Aldoa* | 0.654 | 0.0305 | *Mon1a* | 0.658 | 0.0343 |
| *Mutyh* | 0.650 | 0.0383 | *Sirt6* | 0.654 | 0.0164 | *Drp2* | 0.658 | 0.0035 |
| *Gm13429* | 0.650 | 0.0012 | *Gm2538* | 0.654 | 0.0129 | *Gm4408* | 0.658 | 0.0140 |
| *Mms19* | 0.650 | 0.0201 | *Dact1* | 0.654 | 0.0361 | *Gm8300* | 0.658 | 0.0465 |
| *Aurka* | 0.650 | 0.0409 | *Adam3* | 0.654 | 0.0241 | *Gm16444* | 0.658 | 0.0410 |
| *Col5a3* | 0.651 | 0.0146 | *Clu* | 0.654 | 0.0388 | *2010012O05Rik* | 0.658 | 0.0415 |
| *Palmd* | 0.651 | 0.0380 | *Lcn6* | 0.654 | 0.0492 | *1700091E21Rik* | 0.658 | 0.0442 |
| *Crybb1* | 0.651 | 0.0287 | *Fcrl1* | 0.654 | 0.0209 | *Pde8a* | 0.658 | 0.0017 |
| *Serpina3a* | 0.651 | 0.0165 | *Hbs1l* | 0.654 | 0.0290 | *9030624G23Rik* | 0.658 | 0.0044 |
| *Olfr414* | 0.651 | 0.0064 | *Uty* | 0.655 | 0.0080 | *Rgl3* | 0.658 | 0.0122 |
| *BC089491* | 0.652 | 0.0480 | *Ints6* | 0.655 | 0.0217 | *Iqcd* | 0.658 | 0.0101 |
| *Prdx1* | 0.652 | 0.0256 | *Tbx20* | 0.655 | 0.0440 | *Pld6* | 0.659 | 0.0154 |
| *Gm3459* | 0.652 | 0.0114 | *Mphosph8* | 0.655 | 0.0228 | *Ank2* | 0.659 | 0.0029 |
| *Wfdc15b* | 0.652 | 0.0379 | *Ucma* | 0.655 | 0.0472 | *1110038D17Rik* | 0.659 | 0.0078 |

Table S1-1 (continued)

| **Gene**  **Name** | **Fold change (HFD/Con)** | ***p***  **Value** | **Gene**  **Name** | **Fold change (HFD/Con)** | ***p***  **Value** |
| --- | --- | --- | --- | --- | --- |
| *Gm3727* | 0.659 | 0.0225 | *Bcl2l12* | 0.663 | 0.0012 |
| *4930570E03Rik* | 0.659 | 0.0378 | *Wscd1* | 0.663 | 0.0083 |
| *4930542H20Rik* | 0.659 | 0.0241 | *LOC381390* | 0.663 | 0.0414 |
| *1810009A15Rik* | 0.659 | 0.0410 | *Spns2* | 0.664 | 0.0269 |
| *Tmem212* | 0.659 | 0.0145 | *Tom1* | 0.664 | 0.0029 |
| *Fcer1g* | 0.659 | 0.0159 | *Slc43a2* | 0.664 | 0.0291 |
| *Nhedc2* | 0.660 | 0.0235 | *Mtr* | 0.664 | 0.0035 |
| *Tomm40* | 0.660 | 0.0234 | *Dll1* | 0.664 | 0.0123 |
| *Lrrc51* | 0.660 | 0.0294 | *Klk14* | 0.664 | 0.0003 |
| *Epb4.1* | 0.660 | 0.0005 | *Tha1* | 0.664 | 0.0078 |
| *1810046K07Rik* | 0.660 | 0.0398 | *Phkg2* | 0.664 | 0.0070 |
| *Rangap1* | 0.660 | 0.0304 | *Crocc* | 0.664 | 0.0383 |
| *Ercc1* | 0.660 | 0.0429 | *Sec14l1* | 0.664 | 0.0003 |
| *Gm2438* | 0.660 | 0.0105 | *Eif6* | 0.665 | 0.0092 |
| *Herc1* | 0.661 | 0.0087 | *Gm4699* | 0.665 | 0.0295 |
| *Ufsp1* | 0.661 | 0.0095 | *Sidt1* | 0.665 | 0.0011 |
| *Lass1* | 0.661 | 0.0051 | *Gm8332* | 0.665 | 0.0379 |
| *Atg4d* | 0.661 | 0.0300 | *Col9a3* | 0.665 | 0.0146 |
| *Mett10d* | 0.661 | 0.0306 | *Gm2460* | 0.665 | 0.0396 |
| *Slc7a8* | 0.661 | 0.0288 | *Zfp446* | 0.665 | 0.0281 |
| *Npm3* | 0.661 | 0.0204 | *Hrg* | 0.665 | 0.0236 |
| *Olfr1311* | 0.661 | 0.0019 | *Dleu2* | 0.665 | 0.0206 |
| *1700110K17Rik* | 0.662 | 0.0040 | *Nxnl1* | 0.665 | 0.0472 |
| *Tube1* | 0.662 | 0.0414 | *Dusp27* | 0.665 | 0.0283 |
| *Olfr1462* | 0.662 | 0.0417 | *Esrrb* | 0.665 | 0.0352 |
| *Tsfm* | 0.662 | 0.0430 | *Esyt3* | 0.665 | 0.0369 |
| *6030468B19Rik* | 0.662 | 0.0141 | *Pdia2* | 0.665 | 0.0372 |
| *Olfr512* | 0.662 | 0.0202 | *Gm10030* | 0.666 | 0.0095 |
| *2700038G22Rik* | 0.662 | 0.0045 | *Sept4* | 0.666 | 0.0224 |
| *Clec11a* | 0.662 | 0.0142 | *Slc25a2* | 0.666 | 0.0336 |
| *Oxnad1* | 0.662 | 0.0129 | *Trpv5* | 0.666 | 0.0282 |
| *Trappc2* | 0.663 | 0.0348 | *Gm4679* | 0.666 | 0.0239 |
| *Lmo7* | 0.663 | 0.0315 | *2810408I11Rik* | 0.667 | 0.0232 |
| *LOC100043309* | 0.663 | 0.0161 |  |  |  |
| *Frmd5* | 0.663 | 0.0285 |  |  |  |
| *Pyroxd1* | 0.663 | 0.0015 |  |  |  |
| *Stmn3* | 0.663 | 0.0457 |  |  |  |

Table S1-2 The genes up-regulated expression by HFD

| **Gene**  **Name** | **Fold change (HFD/Con)** | ***p***  **Value** | **Gene**  **Name** | **Fold change (HFD/Con)** | ***p***  **Value** | **Gene**  **Name** | **Fold change (HFD/Con)** | ***p***  **Value** |
| --- | --- | --- | --- | --- | --- | --- | --- | --- |
| *Col6a3* | 2.198 | 0.0002 | *Ccdc97* | 2.775 | 0.0011 | *Sfpq* | 2.394 | 0.0019 |
| *Gabarap* | 2.113 | 0.0002 | *Ptgr1* | 1.654 | 0.0011 | *Helt* | 1.610 | 0.0020 |
| *AU022870* | 2.136 | 0.0003 | *Rnaset2a* | 4.034 | 0.0012 | *Arhgap27* | 1.718 | 0.0020 |
| *Irak1* | 2.227 | 0.0003 | *Safb2* | 1.812 | 0.0012 | *Mapk8ip3* | 1.542 | 0.0020 |
| *Ddx3x* | 2.835 | 0.0003 | *Igl-V1* | 1.743 | 0.0012 | *Gm4419* | 2.196 | 0.0020 |
| *Lyar* | 2.483 | 0.0004 | *Gbx1* | 1.715 | 0.0012 | *Psmb4* | 2.438 | 0.0020 |
| *Bpgm* | 2.863 | 0.0004 | *Acadsb* | 2.009 | 0.0012 | *1700010L13Rik* | 2.044 | 0.0020 |
| *Gyk* | 2.454 | 0.0004 | *Pip5k1c* | 1.978 | 0.0013 | *Psmd14* | 1.577 | 0.0021 |
| *Phf8* | 1.955 | 0.0004 | *Htr4* | 2.947 | 0.0014 | *Cd3eap* | 1.717 | 0.0021 |
| *1700106N22Rik* | 1.990 | 0.0004 | *Sri* | 1.721 | 0.0014 | *D930015E06Rik* | 1.968 | 0.0021 |
| *Syn2* | 1.925 | 0.0004 | *Gsta4* | 1.906 | 0.0015 | *4930556N09Rik* | 1.627 | 0.0022 |
| *Mast1* | 1.995 | 0.0005 | *Pdlim2* | 1.769 | 0.0015 | *Igk* | 1.533 | 0.0022 |
| *Dsn1* | 1.938 | 0.0005 | *Gm10658* | 1.555 | 0.0015 | *4921528I01Rik* | 1.737 | 0.0022 |
| *Rtdr1* | 1.762 | 0.0005 | *Glb1l* | 1.724 | 0.0015 | *Npvf* | 1.500 | 0.0022 |
| *Opn5* | 1.970 | 0.0006 | *Bcat2* | 1.719 | 0.0015 | *Npc2* | 2.092 | 0.0022 |
| *3110083C13Rik* | 1.680 | 0.0006 | *Aatf* | 1.680 | 0.0015 | *C630013B14Rik* | 2.059 | 0.0022 |
| *Zc3h12c* | 1.719 | 0.0006 | *Gm6790* | 2.007 | 0.0016 | *Phactr2* | 1.709 | 0.0022 |
| *Dok6* | 1.669 | 0.0007 | *Zfp395* | 2.426 | 0.0016 | *Calm2* | 2.047 | 0.0023 |
| *Hmgb4* | 2.285 | 0.0007 | *Ifi44* | 2.270 | 0.0016 | *Vmn2r78* | 1.566 | 0.0023 |
| *Srp9* | 2.462 | 0.0007 | *Kcne3* | 1.726 | 0.0016 | *Gm2306* | 1.850 | 0.0024 |
| *Dad1* | 2.432 | 0.0007 | *Gm3516* | 1.817 | 0.0016 | *F730016J06Rik* | 1.882 | 0.0024 |
| *Vmn2r61* | 1.761 | 0.0007 | *F13a1* | 1.546 | 0.0017 | *Gm8129* | 1.698 | 0.0024 |
| *Cps1* | 1.839 | 0.0007 | *Rps25* | 2.016 | 0.0017 | *Mettl7b* | 1.705 | 0.0025 |
| *4933402J07Rik* | 1.973 | 0.0009 | *Degs1* | 1.662 | 0.0017 | *Mtmr7* | 2.118 | 0.0025 |
| *Srp54c* | 1.905 | 0.0009 | *Csde1* | 2.048 | 0.0017 | *Gm12231* | 1.871 | 0.0025 |
| *Rpl12* | 1.868 | 0.0009 | *Vit* | 1.630 | 0.0017 | *Tdrd3* | 2.096 | 0.0025 |
| *Itm2b* | 2.037 | 0.0009 | *Pogz* | 1.871 | 0.0018 | *Tmem39b* | 1.545 | 0.0025 |
| *Gm7027* | 1.544 | 0.0009 | *Dnajb14* | 2.304 | 0.0018 | *6030422M02Rik* | 1.874 | 0.0025 |
| *Asah2* | 1.752 | 0.0010 | *Tas2r114* | 1.758 | 0.0018 | *4930556M19Rik* | 1.755 | 0.0026 |
| *Arpc2* | 1.817 | 0.0010 | *Gm11808* | 1.514 | 0.0018 | *Ephx4* | 1.780 | 0.0026 |
| *Cct6a* | 2.030 | 0.0010 | *Hnrnpd* | 2.062 | 0.0019 | *Itgb6* | 1.927 | 0.0026 |
| *Trim24* | 1.512 | 0.0010 | *Cachd1* | 2.035 | 0.0019 | *Tra2b* | 1.685 | 0.0026 |
| *Ipcef1* | 2.047 | 0.0010 | *Uqcrh* | 2.171 | 0.0019 | *Map4k4* | 1.826 | 0.0026 |
| *Gm16475* | 1.530 | 0.0010 | *Zfp622* | 1.887 | 0.0019 | *Adamts19* | 2.585 | 0.0026 |
| *Gm1974* | 2.663 | 0.0011 | *Tusc5* | 1.885 | 0.0019 | *Pink1* | 1.620 | 0.0026 |
| *Ccr5* | 1.603 | 0.0011 | *Dync1i2* | 1.703 | 0.0019 | *1700022H16Rik* | 1.633 | 0.0027 |

Table S1-2 (continued)

| **Gene**  **Name** | **Fold change (HFD/Con)** | ***p***  **Value** | **Gene**  **Name** | **Fold change (HFD/Con)** | ***p***  **Value** | **Gene**  **Name** | **Fold change (HFD/Con)** | ***p***  **Value** |
| --- | --- | --- | --- | --- | --- | --- | --- | --- |
| *Cdc2l5* | 1.563 | 0.0027 | *Cdc37l1* | 1.580 | 0.0041 | *Cetn1* | 2.256 | 0.0053 |
| *Grap2* | 1.580 | 0.0027 | *Gm6163* | 1.557 | 0.0041 | *Slc38a1* | 2.129 | 0.0053 |
| *Gm412* | 2.111 | 0.0028 | *Gm8074* | 2.013 | 0.0041 | *LOC625853* | 1.557 | 0.0053 |
| *Inppl1* | 1.563 | 0.0029 | *Gm3658* | 1.734 | 0.0041 | *D930016D06Rik* | 1.545 | 0.0053 |
| *Wdr16* | 1.947 | 0.0029 | *Stra6* | 1.590 | 0.0041 | *Gm10241* | 1.520 | 0.0054 |
| *Csn3* | 1.717 | 0.0030 | *Snx13* | 1.747 | 0.0042 | *M6pr* | 1.779 | 0.0054 |
| *Jtb* | 2.021 | 0.0030 | *Ankrd50* | 1.547 | 0.0042 | *Ptpra* | 2.368 | 0.0054 |
| *Zc3h18* | 1.537 | 0.0031 | *Dock6* | 1.698 | 0.0042 | *Vkorc1* | 1.673 | 0.0054 |
| *Gsto1* | 2.184 | 0.0032 | *1810020D17Rik* | 2.385 | 0.0043 | *Rnd3* | 1.676 | 0.0055 |
| *R3hcc1* | 2.212 | 0.0032 | *Mus81* | 1.501 | 0.0043 | *Slc1a7* | 1.573 | 0.0055 |
| *Dock8* | 1.577 | 0.0032 | *Mrpl13* | 1.666 | 0.0043 | *Rpusd4* | 1.881 | 0.0055 |
| *Vps37c* | 1.928 | 0.0032 | *A330074K22Rik* | 1.582 | 0.0043 | *Ncor1* | 2.608 | 0.0056 |
| *Ube2q2* | 1.536 | 0.0032 | *H2-Ab1* | 1.787 | 0.0043 | *Ivns1abp* | 2.096 | 0.0056 |
| *Sva* | 1.719 | 0.0033 | *Rpl4* | 2.200 | 0.0044 | *Asb2* | 1.858 | 0.0056 |
| *Fuca1* | 1.598 | 0.0033 | *Nr2c1* | 1.849 | 0.0044 | *Cartpt* | 2.183 | 0.0057 |
| *Tnc* | 1.506 | 0.0034 | *Zkscan2* | 1.721 | 0.0044 | *Rab11a* | 1.959 | 0.0057 |
| *Gm11353* | 1.618 | 0.0034 | *Il17rd* | 1.835 | 0.0044 | *Zdhhc17* | 2.147 | 0.0058 |
| *Bin2* | 1.619 | 0.0035 | *Mapk12* | 1.996 | 0.0044 | *Sec11c* | 1.690 | 0.0058 |
| *4930519F16Rik* | 1.900 | 0.0035 | *9530002K18Rik* | 1.929 | 0.0045 | *Eef1a1* | 1.591 | 0.0058 |
| *Gsdmc* | 1.627 | 0.0035 | *2410089E03Rik* | 1.612 | 0.0045 | *B2m* | 2.787 | 0.0058 |
| *Gas5* | 2.093 | 0.0036 | *Pde7b* | 1.506 | 0.0046 | *A630001G21Rik* | 2.387 | 0.0058 |
| *Arrdc4* | 1.601 | 0.0036 | *Gpr64* | 1.711 | 0.0046 | *Art2a* | 1.990 | 0.0058 |
| *Hfe* | 1.518 | 0.0036 | *Uqcrq* | 2.099 | 0.0046 | *Utrn* | 1.563 | 0.0059 |
| *Agphd1* | 1.610 | 0.0036 | *Ribc2* | 1.664 | 0.0047 | *Csda* | 1.521 | 0.0059 |
| *Fbxo5* | 1.530 | 0.0036 | *Usp38* | 1.579 | 0.0047 | *Gm15501* | 1.808 | 0.0059 |
| *Tpt1* | 3.065 | 0.0037 | *Olfr26* | 1.600 | 0.0047 | *Eif3c* | 1.605 | 0.0060 |
| *Ccna1* | 1.618 | 0.0037 | *Fam118a* | 1.776 | 0.0047 | *Slc14a2* | 1.914 | 0.0060 |
| *BC055004* | 1.694 | 0.0038 | *Actr6* | 1.849 | 0.0048 | *Srp54a* | 2.116 | 0.0060 |
| *1110020A21Rik* | 1.513 | 0.0038 | *Pcmtd1* | 1.537 | 0.0048 | *Iqgap1* | 1.757 | 0.0061 |
| *Nox4* | 1.717 | 0.0038 | *Dnajb8* | 1.532 | 0.0048 | *BC085271* | 1.863 | 0.0061 |
| *Bnc2* | 1.528 | 0.0038 | *Sp6* | 1.810 | 0.0048 | *Slc25a29* | 1.712 | 0.0061 |
| *Rhox10* | 1.671 | 0.0039 | *Zyg11b* | 1.845 | 0.0049 | *Gm4086* | 1.760 | 0.0061 |
| *Gm5621* | 1.629 | 0.0039 | *Dnm3os* | 2.018 | 0.0049 | *Hsd17b13* | 2.217 | 0.0061 |
| *Gramd1b* | 1.994 | 0.0039 | *Carhsp1* | 1.916 | 0.0050 | *Gm5946* | 1.606 | 0.0062 |
| *Pcdh12* | 2.020 | 0.0040 | *Txn1* | 1.806 | 0.0050 | *Pon2* | 1.732 | 0.0062 |
| *Semg1* | 1.789 | 0.0040 | *Fgd1* | 1.738 | 0.0051 | *Zfp781* | 1.567 | 0.0062 |
| *Ankrd54* | 2.506 | 0.0040 | *Tspan8* | 2.974 | 0.0052 | *Thoc7* | 1.597 | 0.0062 |

Table S1-2 (continued)

| **Gene**  **Name** | **Fold change (HFD/Con)** | ***p***  **Value** | **Gene**  **Name** | **Fold change (HFD/Con)** | ***p***  **Value** | **Gene**  **Name** | **Fold change (HFD/Con)** | ***p***  **Value** |
| --- | --- | --- | --- | --- | --- | --- | --- | --- |
| *Gm6834* | 2.053 | 0.0063 | *S100g* | 1.718 | 0.0077 | *Timd2* | 1.564 | 0.0091 |
| *Ggps1* | 1.976 | 0.0063 | *Gm381* | 1.872 | 0.0077 | *Rabac1* | 2.188 | 0.0091 |
| *1110032A04Rik* | 2.194 | 0.0064 | *Pram1* | 1.949 | 0.0078 | *Spic* | 1.584 | 0.0092 |
| *Dgat1* | 1.610 | 0.0064 | *Vav1* | 1.608 | 0.0079 | *Rce1* | 1.797 | 0.0092 |
| *Gm3842* | 1.966 | 0.0064 | *Gm9934* | 1.616 | 0.0079 | *Suds3* | 1.507 | 0.0092 |
| *Tect1* | 2.111 | 0.0064 | *Tmem38b* | 1.617 | 0.0079 | *Vmn2r79* | 2.538 | 0.0093 |
| *Rps4x* | 1.823 | 0.0064 | *4933403O08Rik* | 1.609 | 0.0079 | *Sh3pxd2b* | 1.861 | 0.0093 |
| *Prkacb* | 1.738 | 0.0065 | *Dlat* | 1.798 | 0.0080 | *Morc1* | 1.866 | 0.0093 |
| *Tmem55a* | 1.594 | 0.0065 | *A130010J15Rik* | 1.839 | 0.0081 | *Zcchc12* | 1.526 | 0.0094 |
| *Aip* | 2.073 | 0.0065 | *Oasl2* | 1.958 | 0.0082 | *Saps2* | 1.666 | 0.0094 |
| *Dcn* | 2.364 | 0.0067 | *Cox4i1* | 1.989 | 0.0082 | *4933437N03Rik* | 1.603 | 0.0094 |
| *Sult1c1* | 2.296 | 0.0068 | *Dffb* | 1.649 | 0.0082 | *Ppil4* | 2.038 | 0.0095 |
| *Ccno* | 1.944 | 0.0068 | *Eomes* | 1.584 | 0.0083 | *4631405K08Rik* | 1.731 | 0.0096 |
| *Kcna6* | 1.821 | 0.0068 | *Ubash3a* | 1.506 | 0.0083 | *Gm2372* | 1.557 | 0.0097 |
| *Cdc7* | 2.220 | 0.0068 | *Zfp651* | 1.526 | 0.0083 | *Gm9093* | 1.622 | 0.0097 |
| *Slc25a42* | 1.544 | 0.0069 | *Lhx4* | 1.699 | 0.0084 | *Map6d1* | 1.776 | 0.0097 |
| *Prss32* | 2.276 | 0.0069 | *Rbm16* | 1.726 | 0.0084 | *I1C0022H11Rik* | 1.699 | 0.0097 |
| *Gm6109* | 1.685 | 0.0069 | *Slc18a1* | 1.758 | 0.0084 | *Arvcf* | 1.500 | 0.0098 |
| *Gm2853* | 1.683 | 0.0069 | *Sh3bgrl3* | 1.902 | 0.0084 | *Gm340* | 1.687 | 0.0098 |
| *Acoxl* | 1.592 | 0.0069 | *Pate4* | 1.798 | 0.0085 | *Gm2593* | 2.100 | 0.0098 |
| *B930086A06Rik* | 1.927 | 0.0070 | *Rras2* | 1.705 | 0.0085 | *Gm8730* | 2.000 | 0.0099 |
| *Cbr4* | 2.276 | 0.0071 | *Mpdu1* | 1.620 | 0.0085 | *1010001N08Rik* | 1.833 | 0.0099 |
| *9530003J23Rik* | 3.010 | 0.0071 | *Fam120c* | 1.660 | 0.0085 | *Gm10316* | 1.595 | 0.0099 |
| *Mpp1* | 2.084 | 0.0072 | *Inpp4b* | 2.366 | 0.0085 | *Rpl7* | 1.561 | 0.0099 |
| *Dopey1* | 1.536 | 0.0073 | *Il7r* | 2.148 | 0.0086 | *Uba52* | 1.680 | 0.0100 |
| *Siglech* | 1.787 | 0.0073 | *1700016G22Rik* | 1.828 | 0.0086 | *Txnl1* | 1.595 | 0.0100 |
| *Hras1* | 2.036 | 0.0073 | *Klhl12* | 1.609 | 0.0086 | *Olfr623* | 2.203 | 0.0102 |
| *Tbp* | 1.943 | 0.0073 | *Arfgef2* | 1.642 | 0.0087 | *Pabpc1* | 2.103 | 0.0103 |
| *Cox11* | 1.909 | 0.0074 | *4930534B04Rik* | 1.686 | 0.0088 | *Uqcrb* | 2.104 | 0.0104 |
| *8430410A17Rik* | 1.650 | 0.0074 | *Prlr* | 1.882 | 0.0088 | *Prdx4* | 1.582 | 0.0104 |
| *Idi1* | 1.571 | 0.0074 | *Nudt16* | 1.859 | 0.0089 | *Rpain* | 1.681 | 0.0104 |
| *Wdr35* | 1.589 | 0.0074 | *Smcr7l* | 1.561 | 0.0089 | *Rrp1b* | 1.781 | 0.0105 |
| *Hcrtr1* | 1.618 | 0.0075 | *Ripk3* | 2.118 | 0.0089 | *BC046331* | 1.755 | 0.0105 |
| *Efhc2* | 1.839 | 0.0075 | *Sh3yl1* | 1.574 | 0.0090 | *Dhx8* | 1.540 | 0.0105 |
| *Ccdc126* | 1.530 | 0.0076 | *Bag1* | 1.614 | 0.0090 | *Acot1* | 1.582 | 0.0106 |
| *Gdf1* | 1.738 | 0.0076 | *Fam162a* | 1.982 | 0.0090 | *Omt2a* | 1.570 | 0.0107 |
| *1700011M02Rik* | 1.531 | 0.0076 | *Gm7044* | 1.813 | 0.0091 | *Il17c* | 2.054 | 0.0107 |

Table S1-2 (continued)

| **Gene**  **Name** | **Fold change (HFD/Con)** | ***p***  **Value** | **Gene**  **Name** | **Fold change (HFD/Con)** | ***p***  **Value** | **Gene**  **Name** | **Fold change (HFD/Con)** | ***p***  **Value** |
| --- | --- | --- | --- | --- | --- | --- | --- | --- |
| *Bmpr1b* | 1.595 | 0.0107 | *Strada* | 1.603 | 0.0123 | *Dmbx1* | 1.727 | 0.0143 |
| *Plscr3* | 1.768 | 0.0107 | *Exosc7* | 1.607 | 0.0125 | *Ankrd1* | 1.638 | 0.0144 |
| *Rps9* | 1.639 | 0.0107 | *Zfp27* | 1.965 | 0.0125 | *Crlf3* | 1.542 | 0.0144 |
| *Gm8040* | 1.533 | 0.0107 | *Arfgap3* | 2.003 | 0.0126 | *D6Mm5e* | 1.514 | 0.0145 |
| *Fam149b* | 1.649 | 0.0108 | *Fgf15* | 1.869 | 0.0127 | *Uba3* | 1.566 | 0.0145 |
| *9230113P08Rik* | 1.624 | 0.0109 | *Gpam* | 1.611 | 0.0128 | *Gm3404* | 1.513 | 0.0145 |
| *Mylk2* | 1.677 | 0.0109 | *Hsdl1* | 1.687 | 0.0129 | *Sod1* | 1.823 | 0.0146 |
| *Phtf2* | 1.609 | 0.0109 | *Sorbs2* | 1.618 | 0.0129 | *Cul1* | 2.697 | 0.0147 |
| *Rps3a* | 1.715 | 0.0110 | *1700095J07Rik* | 1.689 | 0.0129 | *A630095E13Rik* | 3.076 | 0.0147 |
| *Aste1* | 1.853 | 0.0111 | *Ffar1* | 1.677 | 0.0130 | *Tex9* | 2.073 | 0.0147 |
| *D030056L22Rik* | 1.562 | 0.0112 | *Ier5* | 1.511 | 0.0130 | *Myl9* | 1.536 | 0.0148 |
| *Taz* | 1.976 | 0.0113 | *D8Ertd82e* | 1.810 | 0.0131 | *Fam126b* | 2.042 | 0.0148 |
| *Rpl18a* | 1.817 | 0.0113 | *Athl1* | 1.670 | 0.0132 | *Sirt5* | 1.580 | 0.0150 |
| *Npw* | 2.178 | 0.0114 | *BC023744* | 2.023 | 0.0132 | *Dpf3* | 1.589 | 0.0151 |
| *2900057B20Rik* | 1.547 | 0.0115 | *Grm8* | 1.870 | 0.0132 | *Prr22* | 1.780 | 0.0151 |
| *Gm10136* | 1.507 | 0.0115 | *P2rx3* | 2.632 | 0.0133 | *Rplp0* | 1.868 | 0.0152 |
| *Oip5* | 1.641 | 0.0115 | *Gm2648* | 2.064 | 0.0134 | *1190005F20Rik* | 1.730 | 0.0152 |
| *Hdac5* | 1.574 | 0.0116 | *V1rf4* | 1.641 | 0.0134 | *V1rd5* | 1.560 | 0.0152 |
| *Cog6* | 1.517 | 0.0116 | *Hnrnpa2b1* | 1.517 | 0.0135 | *Ccbe1* | 1.819 | 0.0152 |
| *Dag1* | 1.718 | 0.0116 | *Fmo4* | 1.637 | 0.0135 | *Gm7589* | 1.528 | 0.0153 |
| *LOC100270747* | 1.526 | 0.0116 | *Rnls* | 1.504 | 0.0137 | *Gm16500* | 1.975 | 0.0153 |
| *Srpx* | 1.574 | 0.0117 | *Gm5239* | 1.804 | 0.0137 | *Smg7* | 1.889 | 0.0153 |
| *Gm9304* | 1.584 | 0.0117 | *Gm3064* | 1.601 | 0.0137 | *Nlrx1* | 1.761 | 0.0153 |
| *Zfp74* | 1.575 | 0.0118 | *4933431E20Rik* | 2.518 | 0.0138 | *Dnm1* | 1.522 | 0.0154 |
| *Gm4578* | 1.811 | 0.0118 | *BC107364* | 1.710 | 0.0138 | *Gabrb1* | 1.528 | 0.0155 |
| *Pitpna* | 1.511 | 0.0119 | *Nme2* | 1.650 | 0.0139 | *Rnf141* | 1.551 | 0.0155 |
| *Olfr905* | 1.558 | 0.0119 | *D730048I06Rik* | 2.653 | 0.0139 | *Acss2* | 1.678 | 0.0156 |
| *Six2* | 1.652 | 0.0120 | *Tor1aip2* | 1.826 | 0.0139 | *Rps13* | 1.531 | 0.0156 |
| *2010001M06Rik* | 1.560 | 0.0120 | *Fry* | 1.522 | 0.0140 | *Olfr1340* | 1.695 | 0.0156 |
| *Plekhj1* | 2.067 | 0.0120 | *Klra8* | 1.584 | 0.0140 | *Ikzf1* | 1.829 | 0.0157 |
| *Ppp1r11* | 2.097 | 0.0120 | *Rrm2b* | 1.640 | 0.0141 | *Psma7* | 1.503 | 0.0157 |
| *E430018J23Rik* | 1.656 | 0.0120 | *Rasgrp1* | 1.888 | 0.0141 | *Rps17* | 1.560 | 0.0157 |
| *Tbca* | 2.185 | 0.0121 | *Gpnmb* | 1.633 | 0.0141 | *E2f4* | 1.526 | 0.0158 |
| *Gm13242* | 1.531 | 0.0122 | *Ifih1* | 2.092 | 0.0141 | *Cdkl2* | 1.648 | 0.0159 |
| *Slfn14* | 1.755 | 0.0122 | *Pafah1b3* | 1.692 | 0.0142 | *Gm7380* | 1.528 | 0.0159 |
| *Gm8251* | 1.630 | 0.0122 | *Defa21* | 1.621 | 0.0142 | *Cdkl3* | 2.876 | 0.0160 |
| *Slc38a7* | 1.603 | 0.0123 | *Cwc15* | 1.567 | 0.0142 | *Pola2* | 1.815 | 0.0160 |

Table S1-2 (continued)

| **Gene**  **Name** | **Fold change (HFD/Con)** | ***p***  **Value** | **Gene**  **Name** | **Fold change (HFD/Con)** | ***p***  **Value** | **Gene**  **Name** | **Fold change (HFD/Con)** | ***p***  **Value** |
| --- | --- | --- | --- | --- | --- | --- | --- | --- |
| *D19Ertd652e* | 1.691 | 0.0161 | *Prhoxnb* | 1.593 | 0.0181 | *Cnksr3* | 2.174 | 0.0203 |
| *Fate1* | 1.533 | 0.0161 | *Ptprs* | 1.525 | 0.0182 | *Bcor* | 1.658 | 0.0205 |
| *Klhl24* | 1.798 | 0.0162 | *4930483C13Rik* | 1.953 | 0.0183 | *Gm5931* | 1.687 | 0.0205 |
| *Stag1* | 1.532 | 0.0163 | *4930474N05Rik* | 2.013 | 0.0184 | *Sertad1* | 2.137 | 0.0206 |
| *Zbtb4* | 1.660 | 0.0163 | *Zkscan3* | 1.576 | 0.0184 | *Nlrp2* | 1.654 | 0.0206 |
| *Madd* | 1.582 | 0.0165 | *Ctss* | 2.039 | 0.0184 | *Gpatch4* | 1.642 | 0.0207 |
| *Gm5583* | 1.764 | 0.0165 | *Olfr1009* | 2.056 | 0.0184 | *Ankrd16* | 2.350 | 0.0207 |
| *Gltpd1* | 1.780 | 0.0165 | *4930505H01Rik* | 1.931 | 0.0185 | *Stk11ip* | 1.744 | 0.0207 |
| *Mbd3l2* | 1.502 | 0.0166 | *Mgmt* | 1.708 | 0.0185 | *Rbms2* | 1.688 | 0.0208 |
| *Urm1* | 1.582 | 0.0167 | *Nsmce2* | 1.565 | 0.0185 | *Tbc1d9b* | 1.775 | 0.0208 |
| *Asl* | 1.610 | 0.0168 | *Id1* | 1.566 | 0.0186 | *Kifc3* | 1.675 | 0.0208 |
| *Lmo3* | 1.818 | 0.0168 | *Dcaf4* | 1.955 | 0.0186 | *Zfp422-rs1* | 1.517 | 0.0209 |
| *Gm2810* | 1.591 | 0.0169 | *Gm6440* | 1.604 | 0.0187 | *1700064M15Rik* | 1.534 | 0.0209 |
| *Atg2b* | 1.523 | 0.0169 | *Jmjd1c* | 1.612 | 0.0187 | *Abca6* | 1.526 | 0.0211 |
| *Bak1* | 1.519 | 0.0170 | *4930554I06Rik* | 1.688 | 0.0187 | *Rnf125* | 1.663 | 0.0211 |
| *Gm5526* | 1.522 | 0.0170 | *Lrrc63* | 1.635 | 0.0187 | *Vmn2r83* | 2.151 | 0.0212 |
| *Isg15* | 1.868 | 0.0170 | *N4bp1* | 2.048 | 0.0188 | *Tekt5* | 1.576 | 0.0214 |
| *Dcx* | 1.617 | 0.0170 | *Pdzd4* | 1.663 | 0.0188 | *1700016M24Rik* | 1.566 | 0.0214 |
| *Dym* | 1.567 | 0.0172 | *Phc3* | 1.726 | 0.0189 | *Fbxw11* | 1.672 | 0.0215 |
| *Lrp1b* | 1.555 | 0.0173 | *Zfp512* | 1.760 | 0.0190 | *Gm5529* | 1.578 | 0.0215 |
| *Ptk7* | 2.213 | 0.0173 | *Wdr51b* | 1.540 | 0.0191 | *Rnu11* | 1.795 | 0.0217 |
| *BC052040* | 1.791 | 0.0174 | *Qtrtd1* | 1.505 | 0.0191 | *Smek2* | 1.696 | 0.0217 |
| *Rps14* | 1.979 | 0.0174 | *Gtf3a* | 2.035 | 0.0193 | *Trpm7* | 1.583 | 0.0217 |
| *Slit2* | 1.513 | 0.0175 | *Akr1c6* | 1.720 | 0.0193 | *Ftl1* | 1.535 | 0.0217 |
| *Vps28* | 1.766 | 0.0175 | *Kank4* | 1.683 | 0.0193 | *Htt* | 1.808 | 0.0217 |
| *Olfr1280* | 1.524 | 0.0176 | *Gm2073* | 1.679 | 0.0194 | *Gm4910* | 1.677 | 0.0218 |
| *Cdsn* | 1.883 | 0.0177 | *Lsm6* | 1.503 | 0.0194 | *Fbxo30* | 1.677 | 0.0218 |
| *E030019B06Rik* | 1.515 | 0.0177 | *Eif4e* | 1.659 | 0.0195 | *Gm9392* | 2.846 | 0.0219 |
| *1700001K23Rik* | 1.679 | 0.0178 | *Ndufs5* | 1.541 | 0.0196 | *Larp6* | 2.297 | 0.0221 |
| *Atp2c1* | 1.650 | 0.0178 | *Rps3* | 1.546 | 0.0197 | *Tmbim4* | 1.942 | 0.0222 |
| *Svs3a* | 1.748 | 0.0178 | *Gm4941* | 1.640 | 0.0198 | *Maged2* | 1.512 | 0.0223 |
| *Top1* | 2.127 | 0.0179 | *Gm3269* | 1.637 | 0.0199 | *Acsm2* | 1.608 | 0.0224 |
| *Olfr52* | 1.858 | 0.0179 | *Atp6v1b1* | 1.736 | 0.0200 | *Hydin* | 1.521 | 0.0225 |
| *Cpz* | 1.635 | 0.0180 | *Olfr1507* | 1.506 | 0.0200 | *4930573O16Rik* | 1.888 | 0.0225 |
| *Yipf1* | 1.522 | 0.0180 | *9430038I01Rik* | 1.705 | 0.0200 | *Psg23* | 1.740 | 0.0225 |
| *Nbas* | 1.957 | 0.0180 | *Iscu* | 1.664 | 0.0200 | *Mrps18a* | 1.810 | 0.0226 |
| *Aldh2* | 1.816 | 0.0181 | *Gigyf2* | 1.614 | 0.0202 | *Lipg* | 1.587 | 0.0227 |

Table S1-2 (continued)

| **Gene**  **Name** | **Fold change (HFD/Con)** | ***p***  **Value** | **Gene**  **Name** | **Fold change (HFD/Con)** | ***p***  **Value** | **Gene**  **Name** | **Fold change (HFD/Con)** | ***p***  **Value** |
| --- | --- | --- | --- | --- | --- | --- | --- | --- |
| *Gm5296* | 1.549 | 0.0227 | *C230055K05Rik* | 1.623 | 0.0181 | *Olfr1080* | 1.574 | 0.0279 |
| *Npr1* | 1.587 | 0.0227 | *Gm13241* | 1.971 | 0.0182 | *Gm9846* | 1.510 | 0.0281 |
| *Cyp2e1* | 1.533 | 0.0228 | *Pld1* | 1.928 | 0.0183 | *Gjb2* | 2.178 | 0.0282 |
| *Cnpy4* | 1.707 | 0.0229 | *Gm2330* | 2.110 | 0.0184 | *Oprk1* | 2.024 | 0.0282 |
| *Gm6807* | 1.527 | 0.0229 | *AY026312* | 1.993 | 0.0184 | *H2-T22* | 2.296 | 0.0284 |
| *Prkch* | 1.599 | 0.0229 | *Ptp4a2* | 1.714 | 0.0184 | *AI314180* | 2.065 | 0.0285 |
| *H2-Aa* | 1.736 | 0.0229 | *A230083G16Rik* | 1.620 | 0.0184 | *Kcnn2* | 1.501 | 0.0285 |
| *A230065H16Rik* | 1.907 | 0.0230 | *Sdha* | 1.562 | 0.0185 | *Ezh1* | 1.629 | 0.0286 |
| *Ssr3* | 1.552 | 0.0230 | *Aldh3a2* | 2.223 | 0.0185 | *Slc39a13* | 1.763 | 0.0288 |
| *Med10* | 1.533 | 0.0231 | *Mpp6* | 1.890 | 0.0185 | *Ebf1* | 1.792 | 0.0289 |
| *Prosc* | 1.698 | 0.0231 | *Wac* | 1.646 | 0.0186 | *Luzp2* | 1.940 | 0.0289 |
| *Psma1* | 1.636 | 0.0232 | *Gm7429* | 1.547 | 0.0186 | *Atp1b3* | 1.791 | 0.0290 |
| *Ceacam10* | 1.784 | 0.0234 | *Dgcr8* | 1.577 | 0.0187 | *Gm3654* | 1.829 | 0.0290 |
| *Asf1a* | 2.353 | 0.0234 | *Tmem67* | 1.810 | 0.0187 | *Trim35* | 1.781 | 0.0291 |
| *Sectm1a* | 2.102 | 0.0235 | *BC037703* | 1.579 | 0.0187 | *BC048943* | 1.947 | 0.0292 |
| *Eif2b3* | 1.651 | 0.0235 | *Lin28* | 1.700 | 0.0187 | *Rpl10* | 1.644 | 0.0292 |
| *D5Ertd579e* | 1.639 | 0.0237 | *Rapgef4* | 1.768 | 0.0188 | *Hps3* | 1.954 | 0.0293 |
| *Igdcc4* | 1.555 | 0.0237 | *Arid5a* | 1.558 | 0.0188 | *Rps15a* | 1.539 | 0.0293 |
| *Lmod1* | 1.701 | 0.0238 | *Gm4859* | 2.110 | 0.0189 | *Gm11942* | 1.622 | 0.0293 |
| *Slc39a14* | 1.678 | 0.0238 | *Map3k12* | 1.637 | 0.0190 | *Lpp* | 1.549 | 0.0294 |
| *Gm14088* | 1.505 | 0.0239 | *Ttc7* | 1.618 | 0.0191 | *Ntng1* | 2.887 | 0.0298 |
| *Vmn2r81* | 1.683 | 0.0239 | *Syngr4* | 1.708 | 0.0191 | *Mfsd7b* | 3.652 | 0.0299 |
| *Chst8* | 1.951 | 0.0240 | *Lactb2* | 1.894 | 0.0193 | *Gm15072* | 1.629 | 0.0299 |
| *Psmb1* | 1.748 | 0.0240 | *Ttc36* | 1.565 | 0.0193 | *4930435C17Rik* | 2.055 | 0.0299 |
| *Cd200* | 1.672 | 0.0240 | *Lrp1* | 1.750 | 0.0193 | *Havcr1* | 2.544 | 0.0300 |
| *Telo2* | 1.690 | 0.0241 | *Heatr3* | 1.746 | 0.0194 | *Ak3* | 1.893 | 0.0300 |
| *Gm14072* | 1.641 | 0.0241 | *Spag6* | 1.513 | 0.0194 | *Nebl* | 1.524 | 0.0301 |
| *Myt1* | 1.990 | 0.0242 | *Slpi* | 1.533 | 0.0195 | *Rpn2* | 1.547 | 0.0301 |
| *Gm2571* | 1.607 | 0.0243 | *Osbpl10* | 1.613 | 0.0196 | *Orm2* | 1.824 | 0.0305 |
| *D4Wsu53e* | 1.716 | 0.0244 | *A330050B17Rik* | 2.092 | 0.0197 | *A630010A05Rik* | 1.610 | 0.0306 |
| *Bcar1* | 1.988 | 0.0245 | *Usp37* | 1.560 | 0.0198 | *Hrh1* | 1.502 | 0.0307 |
| *Sfrp1* | 1.598 | 0.0245 | *Ephb3* | 2.250 | 0.0199 | *Brunol4* | 1.588 | 0.0308 |
| *Rps18* | 1.626 | 0.0246 | *B830012L14Rik* | 1.551 | 0.0200 | *Gm9000* | 1.925 | 0.0309 |
| *Unc5b* | 1.864 | 0.0247 | *Cypt9* | 1.526 | 0.0200 | *AI854517* | 1.763 | 0.0309 |
| *Hipk1* | 1.600 | 0.0250 | *9030404E10Rik* | 1.763 | 0.0200 | *Fam83d* | 2.159 | 0.0309 |
| *Lcmt1* | 1.586 | 0.0250 | *Olfr112* | 1.677 | 0.0200 | *Zfp239* | 1.517 | 0.0311 |
| *Cgn* | 2.080 | 0.0250 | *Anapc4* | 1.942 | 0.0202 | *Gm12270* | 1.834 | 0.0312 |

Table S1-2 (continued)

| **Gene**  **Name** | **Fold change (HFD/Con)** | ***p***  **Value** | **Gene**  **Name** | **Fold change (HFD/Con)** | ***p***  **Value** | **Gene**  **Name** | **Fold change (HFD/Con)** | ***p***  **Value** |
| --- | --- | --- | --- | --- | --- | --- | --- | --- |
| *Bche* | 1.622 | 0.0312 | *9330162B11Rik* | 1.844 | 0.0347 | *9430024F10Rik* | 1.545 | 0.0383 |
| *Ccr2* | 1.625 | 0.0314 | *Fgf22* | 1.591 | 0.0348 | *Gm10196* | 1.873 | 0.0383 |
| *Cdnf* | 1.507 | 0.0314 | *Pdzk1ip1* | 1.667 | 0.0348 | *Gm5215* | 1.682 | 0.0383 |
| *Zfp263* | 2.229 | 0.0316 | *Cabp4* | 1.560 | 0.0349 | *Pigy* | 1.934 | 0.0384 |
| *Slc39a5* | 1.612 | 0.0316 | *Rpl11* | 1.618 | 0.0349 | *Gm3492* | 1.507 | 0.0385 |
| *Stag2* | 1.602 | 0.0316 | *Hs3st1* | 1.701 | 0.0349 | *Sf3a2* | 1.554 | 0.0387 |
| *Fgr* | 2.217 | 0.0317 | *Atp6v1a* | 1.660 | 0.0351 | *Abcb4* | 1.533 | 0.0387 |
| *Fkbp11* | 1.920 | 0.0320 | *Rad21* | 1.613 | 0.0351 | *Mettl14* | 1.636 | 0.0387 |
| *Gm2447* | 1.624 | 0.0320 | *Myl1* | 1.612 | 0.0353 | *Tes* | 1.762 | 0.0388 |
| *Exoc6* | 1.571 | 0.0320 | *Gm5330* | 1.501 | 0.0354 | *Osbp* | 1.503 | 0.0390 |
| *BC057079* | 1.629 | 0.0321 | *Rbm15* | 1.871 | 0.0354 | *Cnpy2* | 1.614 | 0.0391 |
| *Gm6792* | 1.547 | 0.0321 | *Nudt13* | 1.651 | 0.0354 | *Nsun2* | 1.785 | 0.0393 |
| *Il10ra* | 2.340 | 0.0321 | *2700062C07Rik* | 1.502 | 0.0355 | *Rnf2* | 1.631 | 0.0394 |
| *Zfp53* | 1.553 | 0.0322 | *Gstz1* | 2.435 | 0.0356 | *Cdc14b* | 1.504 | 0.0394 |
| *Mcf2l* | 1.857 | 0.0325 | *Rab3ip* | 1.500 | 0.0357 | *Ttl* | 1.822 | 0.0395 |
| *Hlx* | 1.640 | 0.0326 | *Gm13194* | 1.552 | 0.0358 | *Pfn2* | 2.425 | 0.0395 |
| *Rod1* | 1.685 | 0.0326 | *Setx* | 1.567 | 0.0360 | *Dtna* | 1.505 | 0.0396 |
| *Iffo2* | 1.514 | 0.0327 | *Olfr287* | 1.509 | 0.0361 | *Gm8740* | 1.645 | 0.0397 |
| *Fgf14* | 1.518 | 0.0328 | *Rabggtb* | 1.519 | 0.0362 | *Smchd1* | 1.516 | 0.0397 |
| *A930011G23Rik* | 1.582 | 0.0328 | *Nedd8* | 1.577 | 0.0363 | *Prrc1* | 1.544 | 0.0398 |
| *Fau* | 1.602 | 0.0328 | *BC051142* | 1.555 | 0.0364 | *Csnk1g2* | 1.729 | 0.0398 |
| *Gm5941* | 1.526 | 0.0329 | *Wdr17* | 1.553 | 0.0364 | *Brd2* | 1.641 | 0.0399 |
| *Ly6c2* | 1.705 | 0.0331 | *Elmod1* | 1.641 | 0.0366 | *Gm2798* | 1.506 | 0.0399 |
| *Anp32a* | 1.561 | 0.0332 | *Agpat9* | 2.099 | 0.0366 | *Lyl1* | 1.648 | 0.0400 |
| *2510039O18Rik* | 1.576 | 0.0333 | *P4hb* | 1.646 | 0.0366 | *Pccb* | 1.628 | 0.0400 |
| *Gm7817* | 1.948 | 0.0333 | *Rxrb* | 1.523 | 0.0369 | *Mark1* | 1.886 | 0.0402 |
| *Pdik1l* | 2.494 | 0.0334 | *Crem* | 1.529 | 0.0370 | *Tctn2* | 1.606 | 0.0405 |
| *Uhrf1bp1l* | 1.804 | 0.0335 | *Serbp1* | 1.849 | 0.0370 | *Tnfrsf19* | 1.870 | 0.0405 |
| *Atp5o* | 1.636 | 0.0337 | *2810055G20Rik* | 1.504 | 0.0373 | *Ociad2* | 1.506 | 0.0405 |
| *Crxos1* | 1.579 | 0.0337 | *Smtn* | 2.089 | 0.0373 | *Olfr628* | 1.604 | 0.0406 |
| *Gm10073* | 1.752 | 0.0339 | *Ctbp1* | 1.563 | 0.0376 | *Cux1* | 1.759 | 0.0406 |
| *Pcp4* | 1.651 | 0.0341 | *Fbll1* | 2.001 | 0.0377 | *Fgg* | 1.758 | 0.0406 |
| *Caskin2* | 1.709 | 0.0342 | *Gpx1* | 1.605 | 0.0377 | *Rpgr* | 1.601 | 0.0406 |
| *Gm13083* | 1.522 | 0.0343 | *Gm4190* | 1.594 | 0.0378 | *Mtmr3* | 1.542 | 0.0407 |
| *Olfr187* | 1.630 | 0.0343 | *4921511C20Rik* | 1.579 | 0.0380 | *Gng2* | 1.692 | 0.0409 |
| *Trim17* | 1.654 | 0.0345 | *Ssna1* | 1.660 | 0.0381 | *Gm12680* | 1.874 | 0.0410 |
| *C77080* | 1.506 | 0.0346 | *LOC665306* | 1.604 | 0.0381 | *Rpl6* | 1.769 | 0.0410 |

Table S1-2 (continued)

| **Gene**  **Name** | **Fold change (HFD/Con)** | ***p***  **Value** | **Gene**  **Name** | **Fold change (HFD/Con)** | ***p***  **Value** | **Gene**  **Name** | **Fold change (HFD/Con)** | ***p***  **Value** |
| --- | --- | --- | --- | --- | --- | --- | --- | --- |
| *Trrap* | 1.703 | 0.0411 | *Tle2* | 1.666 | 0.0442 | *Sbno2* | 2.022 | 0.0469 |
| *Msl1* | 2.659 | 0.0412 | *Haao* | 1.533 | 0.0443 | *D230025D16Rik* | 1.534 | 0.0469 |
| *1110007A13Rik* | 1.558 | 0.0412 | *Csnk2a2* | 1.674 | 0.0443 | *Lrch1* | 1.515 | 0.0469 |
| *Nucb2* | 2.511 | 0.0412 | *Col9a1* | 1.893 | 0.0443 | *Gm12296* | 1.735 | 0.0470 |
| *Gm5566* | 1.537 | 0.0413 | *Olfr108* | 1.985 | 0.0444 | *Cep110* | 2.271 | 0.0471 |
| *Pafah1b1* | 1.665 | 0.0413 | *Pdcd6* | 1.628 | 0.0445 | *A830083F22* | 1.658 | 0.0472 |
| *Gm2753* | 2.112 | 0.0414 | *S100a11* | 1.668 | 0.0445 | *Gm15148* | 1.528 | 0.0473 |
| *Zhx2* | 1.616 | 0.0415 | *Yjefn3* | 1.652 | 0.0448 | *Rpl37* | 1.918 | 0.0473 |
| *Gm2224* | 1.520 | 0.0416 | *Stim2* | 1.770 | 0.0450 | *Gpr177* | 1.603 | 0.0475 |
| *Cnot2* | 1.794 | 0.0416 | *Gm12471* | 1.917 | 0.0450 | *Shf* | 1.654 | 0.0475 |
| *Rpl22l1* | 1.732 | 0.0418 | *Defb15* | 1.583 | 0.0450 | *Slc9a8* | 1.679 | 0.0476 |
| *Akr1b10* | 1.864 | 0.0418 | *Atp5g3* | 1.833 | 0.0450 | *Zfp142* | 1.813 | 0.0478 |
| *D5Ertd577e* | 1.640 | 0.0419 | *C330018D20Rik* | 1.582 | 0.0451 | *Lrp4* | 1.733 | 0.0481 |
| *1110054M08Rik* | 1.556 | 0.0420 | *Lefty2* | 1.559 | 0.0452 | *Rbm25* | 1.851 | 0.0482 |
| *Cdc34* | 1.570 | 0.0420 | *Usp7* | 1.675 | 0.0452 | *Rprd1b* | 2.242 | 0.0483 |
| *Tubgcp6* | 1.565 | 0.0421 | *Cdt1* | 1.502 | 0.0453 | *S100a10* | 1.556 | 0.0484 |
| *Klhdc2* | 1.547 | 0.0421 | *Pla2r1* | 1.551 | 0.0455 | *Zbtb10* | 1.591 | 0.0484 |
| *1700007P06Rik* | 1.612 | 0.0423 | *Sntg1* | 1.669 | 0.0457 | *B130055M24Rik* | 1.699 | 0.0484 |
| *Nt5c* | 1.532 | 0.0423 | *Rnf139* | 1.543 | 0.0457 | *Klrb1a* | 1.541 | 0.0487 |
| *Ralgapa2* | 1.788 | 0.0426 | *Gen1* | 2.121 | 0.0459 | *Rg9mtd3* | 1.603 | 0.0488 |
| *Gstm1* | 1.713 | 0.0426 | *Itgb1* | 1.637 | 0.0459 | *Metrn* | 1.828 | 0.0490 |
| *1700044K03Rik* | 1.729 | 0.0427 | *Klhl20* | 1.672 | 0.0460 | *Gm5785* | 1.538 | 0.0490 |
| *Naip6* | 1.549 | 0.0427 | *Cytsa* | 1.655 | 0.0460 | *Pon1* | 1.738 | 0.0491 |
| *3110021N24Rik* | 1.864 | 0.0429 | *Gabrg2* | 2.042 | 0.0461 | *Them5* | 1.612 | 0.0497 |
| *Gm2955* | 1.878 | 0.0430 | *Kcnv1* | 2.330 | 0.0462 | *Gm7079* | 1.579 | 0.0497 |
| *Ypel1* | 1.712 | 0.0432 | *6430598A04Rik* | 1.921 | 0.0463 | *Rnf181* | 1.819 | 0.0498 |
| *Gpr15* | 1.656 | 0.0432 | *Ssx2ip* | 1.531 | 0.0463 | *Sgce* | 1.598 | 0.0499 |
| *Olfr117* | 1.799 | 0.0434 | *Nop10* | 1.857 | 0.0463 | *Ptpru* | 1.742 | 0.0500 |
| *Numbl* | 2.026 | 0.0436 | *Tmem9b* | 1.518 | 0.0463 |  |  |  |
| *Sergef* | 1.910 | 0.0437 | *Tm4sf1* | 1.984 | 0.0465 |  |  |  |
| *Ighmbp2* | 1.779 | 0.0437 | *Nucb1* | 2.238 | 0.0465 |  |  |  |
| *A330035P11Rik* | 2.425 | 0.0438 | *Spred3* | 1.568 | 0.0465 |  |  |  |
| *Elavl2* | 1.505 | 0.0439 | *AY512931* | 1.637 | 0.0466 |  |  |  |
| *Gm3150* | 1.622 | 0.0440 | *Pin1* | 1.575 | 0.0466 |  |  |  |
| *Il31ra* | 1.623 | 0.0440 | *Opa1* | 1.706 | 0.0467 |  |  |  |
| *Slc17a8* | 1.636 | 0.0441 | *Dio3* | 1.847 | 0.0468 |  |  |  |
| *Mtch2* | 1.574 | 0.0442 | Dvl2 | 1.538 | 0.0468 |  |  |  |

Table S2-1 The genes hypermethylated by HFD

| **Gene**  **Name** | **Peak**  **Start** | **Peak End** | **Peak**  **Length** | **Peak**  **Score** | **PeakMValue** | **Promoter_Classification** | **Strand** | **Peak ToTSS** | **TSS** | **TTS** |
| --- | --- | --- | --- | --- | --- | --- | --- | --- | --- | --- |
| *Ncoa2* | 13363870 | 13364339 | 469 | 2.32 | 1.070 | HCP | - | 59 | 13364164 | 13129239 |
| *Cyp27a1* | 74760316 | 74760574 | 258 | 2.43 | 1.453 | ICP | + | 298 | 74760147 | 74784464 |
| *Tmem9* | 137914761 | 137915215 | 454 | 2.4 | 1.505 | ICP | + | 256 | 137914732 | 137931607 |
| *Nhlh1* | 173987241 | 173987487 | 246 | 2.3 | 1.674 | LCP | - | 363 | 173987727 | 173982422 |
| *Acbd3* | 182655904 | 182656152 | 248 | 2.32 | 0.862 | HCP | + | -145 | 182656173 | 182684335 |
| *Kcnk2* | 191167169 | 191167413 | 244 | 3.04 | 1.620 | HCP | - | 292 | 191167583 | 191031808 |
| *Batf3* | 192922303 | 192922542 | 239 | 2.05 | 1.462 | HCP | + | 130 | 192922292 | 192932822 |
| *Heca* | 17667785 | 17668028 | 243 | 2.51 | 2.714 | HCP | - | -33 | 17667873 | 17620271 |
| *Tbpl1* | 22450973 | 22451428 | 455 | 2.5 | 2.004 | HCP | - | 52 | 22451253 | 22423682 |
| *Cd164* | 41239039 | 41239701 | 662 | 2.35 | 1.808 | HCP | + | 65 | 41239305 | 41250848 |
| *Hk1* | 61802208 | 61802853 | 645 | 2.47 | 1.597 | ICP | - | 638 | 61803169 | 61731602 |
| *Hnrnph3* | 62486238 | 62486743 | 505 | 2.42 | 1.160 | HCP | - | 106 | 62486597 | 62477411 |
| *3110049J23Rik* | 62486238 | 62486743 | 505 | 2.42 | 1.160 | HCP | + | -768 | 62487259 | 62521560 |
| *Slc19a1* | 76495429 | 76496074 | 645 | 2.35 | 2.147 | HCP | + | -251 | 76496003 | 76513171 |
| *Gng7* | 80463820 | 80464109 | 289 | 2.18 | 1.158 | LCP | - | 145 | 80464110 | 80411368 |
| *Ckap4* | 83996367 | 83996723 | 356 | 2.37 | 2.421 | HCP | - | 88 | 83996633 | 83989049 |
| *Cry1* | 84647517 | 84647974 | 457 | 2.39 | 1.363 | HCP | - | 53 | 84647799 | 84594444 |
| *Socs2* | 94879537 | 94879796 | 259 | 3.36 | 1.639 | HCP | - | -175 | 94879491 | 94874123 |
| *Gns* | 120802363 | 120802618 | 255 | 2.1 | 1.468 | HCP | + | 345 | 120802145 | 120834301 |
| *Lif* | 4157542 | 4157782 | 240 | 2.29 | 1.022 | LCP | + | 92 | 4157570 | 4172517 |
| *Ikzf1* | 11585332 | 11585864 | 532 | 2.3 | 1.149 | HCP | + | -617 | 11586215 | 11672929 |
| *1110067D22Rik* | 20730626 | 20730872 | 246 | 2.4 | 2.074 | HCP | - | 362 | 20731111 | 20723357 |
| *Usp34* | 23206554 | 23207024 | 470 | 2.4 | 1.871 | HCP | + | -105 | 23206894 | 23390560 |
| *Kcnmb1* | 33862636 | 33862878 | 242 | 2.23 | 1.984 | LCP | + | -255 | 33863012 | 33873638 |
| *Tom1l2* | 60165801 | 60166044 | 243 | 2.35 | 1.098 | HCP | - | 484 | 60166407 | 60054071 |
| *Lrrc48* | 60165801 | 60166044 | 243 | 2.35 | 1.098 | HCP | + | -958 | 60166881 | 60207835 |
| *Ulk2* | 61668310 | 61668853 | 543 | 2.05 | 1.970 | HCP | - | 12 | 61668594 | 61589099 |
| *Per1* | 68911859 | 68912310 | 451 | 2.12 | 1.037 | ICP | + | -372 | 68912457 | 68923459 |
| *Alox12* | 70068551 | 70069003 | 452 | 2.14 | 1.647 | ICP | - | 66 | 70068843 | 70054956 |
| *Inca1* | 70514136 | 70514372 | 236 | 2.3 | 1.692 | HCP | - | -597 | 70513657 | 70501862 |
| *Kif1c* | 70514136 | 70514372 | 236 | 2.3 | 1.692 | HCP | + | 205 | 70514049 | 70545472 |
| *Spns2* | 72303357 | 72303796 | 439 | 2.3 | 1.004 | HCP | - | -170 | 72303406 | 72265139 |
| *Camkk1* | 72831429 | 72832367 | 938 | 2.47 | 1.115 | HCP | + | -611 | 72832509 | 72855567 |
| *Hic1* | 74982285 | 74982534 | 249 | 2.34 | 1.665 | ICP | - | -755 | 74981654 | 74978066 |
| *Cltc* | 86571151 | 86571399 | 248 | 2.15 | 1.307 | HCP | - | -281 | 86570994 | 86508154 |
| *Hoxb5* | 96163746 | 96163980 | 234 | 2.15 | 1.412 | ICP | + | -962 | 96164825 | 96167435 |
| *Tbkbp1* | 97010536 | 97011956 | 1420 | 2.47 | 1.461 | LCP | - | -220 | 97011026 | 96997484 |

Table S2-1 (continued)

| **Gene**  **Name** | **Peak**  **Start** | **Peak End** | **Peak**  **Length** | **Peak**  **Score** | **PeakMValue** | **Promoter_Classification** | **Strand** | **Peak ToTSS** | **TSS** | **TTS** |
| --- | --- | --- | --- | --- | --- | --- | --- | --- | --- | --- |
| *Msl1* | 98656436 | 98657192 | 756 | 2.22 | 1.485 | HCP | + | -268 | 98657082 | 98669173 |
| *Rara* | 98799194 | 98800108 | 914 | 2.38 | 2.051 | ICP | + | 642 | 98799009 | 98836256 |
| *Cygb* | 116515491 | 116516277 | 786 | 2.23 | 1.203 | ICP | - | -257 | 116515627 | 116506908 |
| *Dtnb* | 3572065 | 3572313 | 248 | 2.46 | 1.796 | HCP | + | -201 | 3572390 | 3781398 |
| *2810032G03Rik* | 5382002 | 5382413 | 411 | 2.55 | 2.530 | ICP | + | -1099 | 5383307 | 5422938 |
| *Klhl29* | 5382002 | 5382413 | 411 | 2.55 | 2.530 | HCP | - | 280 | 5382488 | 5084273 |
| *Tmem30b* | 74646888 | 74647142 | 254 | 2.77 | 2.940 | HCP | - | 367 | 74647382 | 74644100 |
| *Rgs6* | 83717831 | 83718078 | 247 | 2.1 | 2.742 | HCP | + | -69 | 83718024 | 84259799 |
| *2310044G17Rik* | 88288418 | 88288669 | 251 | 2.48 | 2.376 | HCP | + | 251 | 88288292 | 88306314 |
| *Rps6ka5* | 101962739 | 101963199 | 460 | 2.46 | 1.534 | HCP | - | 269 | 101963238 | 101787987 |
| *Cyp46a1* | 109571911 | 109572156 | 245 | 2.26 | 1.076 | HCP | + | -556 | 109572590 | 109600444 |
| *Dio3* | 111516853 | 111517933 | 1080 | 2.38 | 1.760 | HCP | + | -46 | 111517439 | 111519307 |
| *Dio3os* | 111516853 | 111517933 | 1080 | 2.38 | 1.760 | HCP | - | -1115 | 111516278 | 111513594 |
| *Mir1247* | 111516853 | 111517933 | 1080 | 2.38 | 1.760 | HCP | - | -1054 | 111516339 | 111516257 |
| *Klc1* | 112997143 | 112997395 | 252 | 2.5 | 2.014 | HCP | + | 210 | 112997059 | 113033110 |
| *Zbtb42* | 113917089 | 113917407 | 318 | 2.38 | 1.951 | HCP | + | 198 | 113917050 | 113920958 |
| *Vipr2* | 117315728 | 117316170 | 442 | 2.24 | 1.699 | ICP | + | -246 | 117316195 | 117384734 |
| *Esyt2* | 117519718 | 117519967 | 249 | 2.53 | 2.055 | HCP | + | 148 | 117519694 | 117611571 |
| *Kdm1b* | 47139199 | 47139863 | 664 | 2.36 | 1.518 | ICP | + | 624 | 47138907 | 47179982 |
| *4732471D19Rik* | 54605123 | 54605591 | 468 | 2.32 | 2.604 | HCP | + | 192 | 54605165 | 54652651 |
| *B230219D22Rik* | 55794620 | 55794857 | 237 | 2.37 | 1.689 | HCP | + | 254 | 55794484 | 55804861 |
| *Irx2* | 72766509 | 72767044 | 535 | 2.52 | 2.520 | HCP | + | 351 | 72766425 | 72771642 |
| *D430050G20* | 72766509 | 72767044 | 535 | 2.52 | 2.520 | HCP | - | -764 | 72766012 | 72760913 |
| *Mast4* | 104124092 | 104124349 | 257 | 2.44 | 2.253 | HCP | - | 351 | 104124572 | 103522568 |
| *Snx18* | 114408683 | 114408935 | 252 | 2.33 | 1.780 | HCP | - | -37 | 114408772 | 114382386 |
| *Plac9* | 26722323 | 26722560 | 237 | 2.27 | 1.574 | ICP | - | -62 | 26722379 | 26707888 |
| *Erc2* | 28435505 | 28435765 | 260 | 2.04 | 2.227 | HCP | + | 8 | 28435627 | 29291723 |
| *Bmpr1a* | 35315463 | 35316042 | 579 | 2.21 | 1.509 | HCP | - | -20 | 35315732 | 35224253 |
| *Dleu2* | 62301129 | 62301835 | 706 | 2.16 | 1.844 | HCP | - | -272 | 62301210 | 62221672 |
| *Pdlim2* | 70576790 | 70577038 | 248 | 2.09 | 1.751 | LCP | - | 565 | 70577479 | 70564024 |
| *Tmtc4* | 123382198 | 123383059 | 861 | 2.4 | 2.130 | HCP | - | -145 | 123382483 | 123318196 |
| *Fam83a* | 57816582 | 57817257 | 675 | 2.19 | 0.940 | LCP | + | -537 | 57817457 | 57842257 |
| *Hsf1* | 76307667 | 76307924 | 257 | 2.13 | 1.163 | HCP | + | -78 | 76307874 | 76331402 |
| *Bop1* | 76307667 | 76307924 | 257 | 2.13 | 1.163 | HCP | - | -96 | 76307699 | 76283425 |
| *Elfn2* | 78548500 | 78549064 | 564 | 2.22 | 1.924 | HCP | - | -239 | 78548543 | 78500436 |
| *Gcat* | 78861348 | 78861608 | 260 | 2.56 | 2.389 | HCP | + | 175 | 78861303 | 78868786 |
| *Micall1* | 78939491 | 78939751 | 260 | 2.61 | 1.847 | HCP | + | 209 | 78939412 | 78967331 |
| *Pdgfb* | 79845020 | 79845499 | 479 | 2.56 | 1.474 | ICP | - | -21 | 79845238 | 79826305 |

Table S2-1 (continued)

| **Gene**  **Name** | **Peak**  **Start** | **Peak End** | **Peak**  **Length** | **Peak**  **Score** | **PeakMValue** | **Promoter_Classification** | **Strand** | **Peak ToTSS** | **TSS** | **TTS** |
| --- | --- | --- | --- | --- | --- | --- | --- | --- | --- | --- |
| *Xrcc6* | 81846334 | 81846661 | 327 | 2.64 | 1.730 | HCP | + | -300 | 81846798 | 81870514 |
| *Pppde2* | 81846334 | 81846661 | 327 | 2.64 | 1.730 | HCP | - | 72 | 81846570 | 81822952 |
| *Prickle1* | 93425838 | 93426072 | 234 | 2.81 | 2.735 | HCP | - | 367 | 93426322 | 93329544 |
| *Irak4* | 94373900 | 94374155 | 255 | 2.19 | 1.847 | ICP | + | -62 | 94374090 | 94398747 |
| *Pus7l* | 94373900 | 94374155 | 255 | 2.19 | 1.847 | HCP | - | -89 | 94373938 | 94353070 |
| *Parn* | 13667778 | 13668233 | 455 | 2.6 | 1.545 | ICP | - | 257 | 13668263 | 13538056 |
| *Rtp2* | 23931479 | 23931760 | 281 | 2.29 | 1.247 | LCP | - | -739 | 23930880 | 23925633 |
| *Ripply3* | 94550229 | 94550470 | 241 | 2.23 | 1.359 | HCP | + | 321 | 94550028 | 94558542 |
| *Prr18* | 8533831 | 8534080 | 249 | 2.53 | 1.860 | ICP | + | 352 | 8533603 | 8536978 |
| *Dll1* | 15512748 | 15513000 | 252 | 2.49 | 1.093 | HCP | - | -87 | 15512787 | 15504317 |
| *Mmp25* | 23781752 | 23781999 | 247 | 2.09 | 1.451 | ICP | - | 360 | 23782236 | 23766424 |
| *Pgp* | 24606892 | 24607146 | 254 | 2.33 | 2.266 | HCP | + | -398 | 24607417 | 24608541 |
| *Ift140* | 25152713 | 25153240 | 527 | 2.55 | 1.654 | HCP | + | -53 | 25153030 | 25236442 |
| *Cramp1l* | 25152713 | 25153240 | 527 | 2.55 | 1.654 | HCP | - | -801 | 25152175 | 25098170 |
| *Sstr5* | 25634591 | 25635210 | 619 | 2.28 | 1.126 | ICP | - | -667 | 25634233 | 25626819 |
| *Pdia2* | 26336366 | 26336616 | 250 | 2.33 | 0.984 | LCP | - | -459 | 26336032 | 26332943 |
| *Mdga1* | 30024943 | 30025269 | 326 | 2.21 | 1.398 | HCP | - | -279 | 30024827 | 29964902 |
| *Sema6b* | 56279322 | 56280869 | 1547 | 2.96 | 2.101 | HCP | - | -329 | 56279766 | 56262507 |
| *Lonp1* | 56765827 | 56766086 | 259 | 2.53 | 2.394 | HCP | - | 369 | 56766326 | 56753721 |
| *Vapa* | 65962557 | 65963034 | 477 | 2.44 | 2.442 | HCP | - | 99 | 65962895 | 65929392 |
| *1110012J17Rik* | 66799052 | 66799513 | 461 | 2.56 | 1.633 | HCP | - | -192 | 66799090 | 66686321 |
| *Cyp1b1* | 80114497 | 80114952 | 455 | 2.17 | 0.878 | ICP | - | -343 | 80114381 | 80106292 |
| *Fbxo11* | 88464550 | 88464957 | 407 | 2.54 | 2.768 | HCP | - | -128 | 88464625 | 88390198 |
| *Zfp438* | 5333951 | 5334191 | 240 | 2.5 | 1.529 | HCP | - | 366 | 5334437 | 5210028 |
| *Ube2d2* | 35931279 | 35931515 | 236 | 2.29 | 1.509 | HCP | + | 185 | 35931212 | 35966826 |
| *Pggt1b* | 46440009 | 46440269 | 260 | 2.51 | 2.237 | HCP | - | 365 | 46440504 | 46399602 |
| *Synpo* | 60770013 | 60770953 | 940 | 2.46 | 1.419 | LCP | - | -725 | 60769758 | 60759859 |
| *D18Ertd653e* | 68092861 | 68093178 | 317 | 2.29 | 0.952 | HCP | + | 109 | 68092910 | 68415203 |
| *Cxxc1* | 74376006 | 74376260 | 254 | 2.45 | 2.272 | HCP | + | 268 | 74375865 | 74381145 |
| *Loxhd1* | 77519814 | 77520735 | 921 | 2.4 | 1.569 | ICP | + | -421 | 77520696 | 77680996 |
| *Nfatc1* | 80909390 | 80910382 | 992 | 2.75 | 1.959 | HCP | - | -76 | 80909810 | 80844173 |
| *Eif1ad* | 5366872 | 5367118 | 246 | 2.9 | 2.440 | HCP | + | 183 | 5366812 | 5371511 |
| *Banf1* | 5366872 | 5367118 | 246 | 2.9 | 2.440 | HCP | - | -350 | 5366645 | 5364639 |
| *Arl2* | 6141063 | 6141297 | 234 | 2.27 | 1.691 | HCP | - | -43 | 6141137 | 6134388 |
| *Fads3* | 10116101 | 10116336 | 235 | 2.16 | 1.702 | HCP | + | 181 | 10116037 | 10134161 |
| *Uhrf2* | 30105021 | 30105490 | 469 | 2.47 | 2.551 | HCP | + | 253 | 30105002 | 30168214 |
| *Prkg1* | 31738566 | 31738827 | 261 | 2.25 | 1.139 | ICP | - | 163 | 31738860 | 30643036 |
| *Fbxo18* | 11699421 | 11700300 | 879 | 3.39 | 1.911 | HCP | - | -706 | 11699154 | 11664199 |

Table S2-1 (continued)

| **Gene**  **Name** | **Peak**  **Start** | **Peak End** | **Peak**  **Length** | **Peak**  **Score** | **PeakMValue** | **Promoter_Classification** | **Strand** | **Peak ToTSS** | **TSS** | **TTS** |
| --- | --- | --- | --- | --- | --- | --- | --- | --- | --- | --- |
| *Ankrd16* | 11699421 | 11700300 | 879 | 3.39 | 1.911 | HCP | + | 481 | 11699379 | 11711950 |
| *Cacnb2* | 14526026 | 14526350 | 324 | 2.55 | 2.042 | HCP | + | 256 | 14525932 | 14908560 |
| *Cacna1b* | 24618186 | 24619059 | 873 | 2.24 | 1.574 | HCP | - | -55 | 24618567 | 24462089 |
| *Cobra1* | 25066513 | 25066970 | 457 | 2.09 | 1.983 | ICP | - | 267 | 25067009 | 25055231 |
| *Col5a1* | 27741510 | 27741861 | 351 | 2.2 | 1.614 | HCP | + | -258 | 27741944 | 27895030 |
| *Endog* | 30026843 | 30027093 | 250 | 2.3 | 1.011 | HCP | + | -75 | 30027043 | 30029589 |
| *Prdm12* | 31495758 | 31496008 | 250 | 2.33 | 1.529 | ICP | + | 327 | 31495556 | 31511315 |
| *Fibcd1* | 31702014 | 31702313 | 299 | 2.59 | 1.858 | HCP | - | -638 | 31701525 | 31668809 |
| *Lhx6* | 35959811 | 35960540 | 729 | 2.72 | 1.554 | ICP | - | -595 | 35959580 | 35937472 |
| *Lrp2* | 69424255 | 69424495 | 240 | 2.15 | 0.782 | HCP | - | -251 | 69424124 | 69262391 |
| *Dgkz* | 91803761 | 91804469 | 708 | 2.47 | 1.278 | HCP | - | -395 | 91803720 | 91772978 |
| *Pak6* | 118503002 | 118503237 | 235 | 2.09 | 2.033 | HCP | + | 138 | 118502981 | 118523756 |
| *Gm14207* | 119152054 | 119152573 | 519 | 2.37 | 1.725 | HCP | - | -380 | 119151933 | 119146934 |
| *Myef2* | 124948908 | 124949156 | 248 | 2.18 | 1.436 | HCP | - | 364 | 124949396 | 124913477 |
| *Kcnip3* | 127347512 | 127347763 | 251 | 2.31 | 1.488 | LCP | - | -531 | 127347106 | 127282233 |
| *Crls1* | 132672932 | 132673176 | 244 | 2 | 0.884 | HCP | + | -415 | 132673469 | 132692504 |
| *Rem1* | 152452743 | 152453004 | 261 | 2.41 | 1.542 | ICP | + | 130 | 152452743 | 152460927 |
| *Mapre1* | 153567123 | 153567486 | 363 | 2.09 | 1.743 | HCP | + | 282 | 153567022 | 153599050 |
| *Trp53inp2* | 155207785 | 155208033 | 248 | 2.18 | 1.996 | HCP | + | 318 | 155207591 | 155215583 |
| *D630003M21Rik* | 158054785 | 158055370 | 585 | 2.36 | 1.810 | ICP | - | -119 | 158054958 | 158021483 |
| *Fam83d* | 158593819 | 158594067 | 248 | 2.07 | 1.605 | HCP | + | 109 | 158593834 | 158612373 |
| *Plcg1* | 160557009 | 160557258 | 249 | 2.91 | 2.365 | HCP | + | 88 | 160557045 | 160601496 |
| *Chd6* | 160934359 | 160934656 | 297 | 2.51 | 1.580 | HCP | - | 284 | 160934792 | 160772713 |
| *Tomm34* | 163896753 | 163896998 | 245 | 2.07 | 1.825 | HCP | - | -37 | 163896838 | 163879276 |
| *4833422F24Rik* | 165226120 | 165226602 | 482 | 2.27 | 1.089 | LCP | - | -467 | 165225894 | 165220949 |
| *Magi3* | 104023880 | 104024201 | 321 | 2.53 | 2.237 | HCP | - | 288 | 104024329 | 103817187 |
| *Pdlim5* | 142058161 | 142058416 | 255 | 2.49 | 2.227 | HCP | - | 371 | 142058660 | 141965043 |
| *4930412F15Rik* | 43682622 | 43682935 | 313 | 2.49 | 1.793 | HCP | - | -1047 | 43681731 | 43672493 |
| *Foxe1* | 46356459 | 46356721 | 262 | 2.27 | 1.622 | HCP | + | -475 | 46357065 | 46358181 |
| *4930473A06Rik* | 83171662 | 83171925 | 263 | 2.06 | 1.292 | HCP | + | 345 | 83171448 | 83510574 |
| *Dock7* | 98787121 | 98787368 | 247 | 2.43 | 1.644 | HCP | - | 361 | 98787606 | 98603355 |
| *Eif2c1* | 126145167 | 126145430 | 263 | 2.39 | 1.576 | HCP | - | 366 | 126145665 | 126112256 |
| *Gpr3* | 132767959 | 132768215 | 256 | 2.69 | 2.231 | HCP | - | 364 | 132768451 | 132765254 |
| *Wnt4* | 136833554 | 136833806 | 252 | 2.34 | 1.679 | HCP | + | 131 | 136833549 | 136852694 |
| *Nadk* | 154937841 | 154938309 | 468 | 2.49 | 1.682 | HCP | + | 152 | 154937923 | 154965110 |
| *Phtf2* | 20387603 | 20387840 | 237 | 2.71 | 1.361 | HCP | - | 220 | 20387942 | 20264481 |
| *Tmem60* | 20387603 | 20387840 | 237 | 2.71 | 1.361 | HCP | + | -548 | 20388270 | 20392688 |
| *A630072M18Rik* | 20456864 | 20457113 | 249 | 2.09 | 2.132 | ICP | + | 182 | 20456806 | 20462216 |

Table S2-1 (continued)

| **Gene**  **Name** | **Peak**  **Start** | **Peak End** | **Peak**  **Length** | **Peak**  **Score** | **PeakMValue** | **Promoter_Classification** | **Strand** | **Peak ToTSS** | **TSS** | **TTS** |
| --- | --- | --- | --- | --- | --- | --- | --- | --- | --- | --- |
| *Ube3c* | 29895392 | 29896086 | 694 | 2.13 | 2.184 | HCP | + | -42 | 29895781 | 30002617 |
| *Cpz* | 35868190 | 35868442 | 252 | 2.29 | 1.002 | ICP | - | -41 | 35868275 | 35844866 |
| *Slain2* | 73305840 | 73306077 | 237 | 2.27 | 3.147 | HCP | + | 403 | 73305555 | 73370067 |
| *Tpst1* | 130555456 | 130555703 | 247 | 2.38 | 1.490 | HCP | + | 224 | 130555355 | 130611602 |
| *Elfn1* | 140383132 | 140383905 | 773 | 2.45 | 1.557 | ICP | + | -377 | 140383896 | 140450678 |
| *Chst12* | 140981227 | 140981486 | 259 | 2.34 | 1.591 | HCP | + | -205 | 140981562 | 141001192 |
| *Lmod2* | 24547113 | 24547366 | 253 | 2.07 | 0.758 | LCP | + | -530 | 24547770 | 24555414 |
| *Wasl* | 24614572 | 24614835 | 263 | 2.27 | 1.227 | HCP | - | 291 | 24614995 | 24582685 |
| *Cul1* | 47404106 | 47404622 | 516 | 2.65 | 1.713 | HCP | + | 42 | 47404322 | 47476138 |
| *Zfp467* | 48394738 | 48395197 | 459 | 2.65 | 1.880 | HCP | - | 121 | 48395089 | 48386611 |
| *Gars* | 54988022 | 54988272 | 250 | 2.06 | 2.928 | HCP | + | 153 | 54987994 | 55029498 |
| *Rnf103* | 71443918 | 71444378 | 460 | 2.52 | 1.724 | HCP | + | 261 | 71443887 | 71460875 |
| *Bola3* | 83299643 | 83299903 | 260 | 2.2 | 1.238 | HCP | + | 296 | 83299477 | 83308386 |
| *Klf15* | 90412347 | 90412862 | 515 | 2.54 | 2.162 | HCP | + | -14 | 90412619 | 90425203 |
| *Brpf1* | 113257328 | 113257581 | 253 | 2.48 | 1.675 | HCP | + | 264 | 113257190 | 113274698 |
| *Rassf4* | 116623426 | 116623872 | 446 | 2.17 | 1.460 | ICP | - | 205 | 116623854 | 116583025 |
| *Peg3* | 6682361 | 6683222 | 861 | 2.31 | 1.525 | ICP | - | 338 | 6683130 | 6658670 |
| *Usp29* | 6682361 | 6683222 | 861 | 2.31 | 1.525 | ICP | + | -659 | 6683451 | 6919931 |
| *Prr24* | 16858769 | 16859210 | 441 | 2.64 | 1.196 | ICP | - | 51 | 16859041 | 16857361 |
| *Mark4* | 20043552 | 20044268 | 716 | 2.22 | 1.703 | HCP | - | -67 | 20043843 | 20011423 |
| *Kcnk6* | 30017055 | 30017729 | 674 | 2.47 | 1.930 | HCP | - | 149 | 30017541 | 30006946 |
| *Spint2* | 30066510 | 30066751 | 241 | 2.48 | 1.934 | HCP | - | 365 | 30066996 | 30041348 |
| *Arhgap33* | 31319595 | 31319955 | 360 | 2.33 | 1.395 | HCP | - | 253 | 31320028 | 31307244 |
| *Wbp7* | 31374037 | 31374779 | 742 | 2.4 | 1.193 | HCP | - | -663 | 31373745 | 31353873 |
| *Fxyd5* | 31827070 | 31827340 | 270 | 2.2 | 2.215 | ICP | - | -368 | 31826837 | 31817741 |
| *Fgf21* | 52870574 | 52870830 | 256 | 2.4 | 2.044 | LCP | - | 158 | 52870860 | 52869259 |
| *Aldh1a3* | 73571987 | 73572236 | 249 | 2.33 | 1.648 | HCP | - | 251 | 73572363 | 73535778 |
| *Lysmd4* | 74367367 | 74367845 | 478 | 2.48 | 1.573 | ICP | + | 105 | 74367501 | 74373354 |
| *Mef2a* | 74517707 | 74517958 | 251 | 2.77 | 1.950 | HCP | - | -88 | 74517744 | 74378716 |
| *Adamtsl3* | 89483326 | 89484595 | 1269 | 2.38 | 2.043 | HCP | + | -242 | 89484203 | 89762958 |
| *Rab6* | 107756431 | 107757184 | 753 | 2.49 | 2.038 | HCP | + | 708 | 107756099 | 107789782 |
| *Mrpl48* | 107756431 | 107757184 | 753 | 2.49 | 2.038 | HCP | - | -297 | 107756510 | 107697630 |
| *Prkcb* | 129432871 | 129433105 | 234 | 2.68 | 2.584 | HCP | + | 350 | 129432638 | 129777915 |
| *Il21r* | 132746746 | 132746984 | 238 | 2.03 | 1.677 | LCP | + | -77 | 132746942 | 132777084 |
| *Prr14* | 134615320 | 134615580 | 260 | 2.46 | 1.517 | HCP | + | 323 | 134615127 | 134620272 |
| *Setd1a* | 134920779 | 134921035 | 256 | 2.41 | 1.669 | HCP | + | 5 | 134920902 | 134943633 |
| *Pddc1* | 148599531 | 148599801 | 270 | 2.26 | 1.538 | HCP | - | 358 | 148600024 | 148594082 |
| *Cd151* | 148653588 | 148654649 | 1061 | 2.72 | 1.754 | LCP | + | -568 | 148654687 | 148657380 |

Table S2-1 (continued)

| **Gene**  **Name** | **Peak**  **Start** | **Peak End** | **Peak**  **Length** | **Peak**  **Score** | **PeakM-Value** | **Promoter_C-lassification** | **Strand** | **Peak ToTSS** | **TSS** | **TTS** |
| --- | --- | --- | --- | --- | --- | --- | --- | --- | --- | --- |
| *Dlc1* | 37676498 | 37676747 | 249 | 2.48 | 2.410 | HCP | - | 374 | 37676997 | 37630792 |
| *Tmem59l* | 73011949 | 73012209 | 260 | 2.62 | 1.583 | ICP | - | -822 | 73011257 | 73007769 |
| *Pgpep1* | 73183143 | 73183686 | 543 | 2.24 | 1.982 | HCP | - | 165 | 73183580 | 73173009 |
| *Nr2f6* | 73905897 | 73906161 | 264 | 2.32 | 1.598 | HCP | - | -178 | 73905851 | 73898017 |
| *Adcy7* | 90817308 | 90817969 | 661 | 2.21 | 0.968 | LCP | + | -463 | 90818102 | 90853861 |
| *Tmem208* | 107849379 | 107849633 | 254 | 2.25 | 1.453 | HCP | + | -757 | 107850263 | 107852957 |
| *Plekhg4* | 107898896 | 107899149 | 253 | 2.39 | 0.982 | ICP | + | -257 | 107899280 | 107906762 |
| *Znrf1* | 114061167 | 114061411 | 244 | 2.03 | 1.737 | LCP | + | -953 | 114062242 | 114149930 |
| *Gse1* | 123011470 | 123012693 | 1223 | 2.54 | 1.878 | ICP | + | -683 | 123012765 | 123105283 |
| *Mvd* | 124966990 | 124967228 | 238 | 2.23 | 1.002 | HCP | - | 213 | 124967322 | 124957495 |
| *BC021891* | 128433565 | 128434233 | 668 | 2.59 | 1.425 | ICP | + | -450 | 128434349 | 128471339 |
| *Irf2bp2* | 129117667 | 129118468 | 801 | 2.33 | 1.575 | HCP | - | -731 | 129117336 | 129112195 |
| *1810026J23Rik* | 21397360 | 21397604 | 244 | 2.04 | 1.392 | ICP | + | 317 | 21397165 | 21400391 |
| *Yipf2* | 21397360 | 21397604 | 244 | 2.04 | 1.392 | HCP | - | -292 | 21397190 | 21393125 |
| *Hspa8* | 40609109 | 40609346 | 237 | 2.38 | 0.872 | HCP | + | -127 | 40609355 | 40613282 |
| *AI593442* | 52487108 | 52487389 | 281 | 2.54 | 1.230 | ICP | - | 285 | 52487534 | 52481146 |
| *Sin3a* | 56920296 | 56920991 | 695 | 2.51 | 2.203 | ICP | + | 797 | 56919846 | 56976175 |
| *Myo1e* | 70055051 | 70055490 | 439 | 2.01 | 1.530 | HCP | + | 114 | 70055156 | 70247874 |
| *Mapk6* | 75257282 | 75257778 | 496 | 2.33 | 2.202 | HCP | - | -364 | 75257166 | 75234707 |
| *Hmgcll1* | 75862673 | 75862932 | 259 | 2.18 | 1.026 | ICP | + | 19 | 75862783 | 75984157 |
| *Atp1b3* | 96264228 | 96264520 | 292 | 2.44 | 1.569 | HCP | - | 344 | 96264718 | 96233094 |
| *Glyctk* | 106059985 | 106060234 | 249 | 2.45 | 2.244 | LCP | - | 359 | 106060469 | 106055190 |
| *Dock3* | 107133748 | 107134041 | 293 | 2.76 | 2.454 | HCP | - | 345 | 107134240 | 106795155 |
| *Apeh* | 107996737 | 107996971 | 234 | 2.29 | 1.688 | HCP | - | -43 | 107996811 | 107987744 |
| *Gm5634* | 8540322 | 8540582 | 260 | 2.01 | 0.454 | ICP | - | -351 | 8540101 | 8539259 |
| *Nyx* | 13044026 | 13044477 | 451 | 2.26 | 0.944 | LCP | + | -545 | 13044797 | 13066439 |
| *Dock11* | 33428885 | 33429130 | 245 | 2.38 | 1.728 | ICP | + | 181 | 33428826 | 33616557 |
| *Hs6st2* | 49033157 | 49034392 | 1235 | 3.02 | 2.154 | HCP | - | -120 | 49033654 | 48740388 |
| *Gpc3* | 49967091 | 49967339 | 248 | 2.35 | 1.332 | HCP | - | -64 | 49967151 | 49625603 |
| *Plac1* | 50467824 | 50468086 | 262 | 2.05 | 1.313 | LCP | - | -373 | 50467582 | 50423178 |
| *2610030H06Rik* | 69061332 | 69061796 | 464 | 2.24 | 1.388 | HCP | + | -553 | 69062117 | 69070045 |
| *Nsdhl* | 70163504 | 70163959 | 455 | 2.01 | 1.325 | HCP | + | -127 | 70163859 | 70203867 |
| *Cetn2* | 70163504 | 70163959 | 455 | 2.01 | 1.325 | HCP | - | -48 | 70163683 | 70158903 |
| *Sh3kbp1* | 156065456 | 156065699 | 243 | 2.36 | 1.733 | HCP | + | 238 | 156065339 | 156413849 |
| *Rbbp7* | 159198142 | 159198516 | 374 | 2.26 | 2.561 | HCP | + | 26 | 159198303 | 159217022 |

GeneName: The gene which the peak overlaps its promoter region; PeakStart: The start base pair of the peak on the chromosome; PeakEnd: The end base pair of the peak on the chromosome; PeakLength: The length of the peak; PeakScore: The average -log10 (P-value) from probes within the peak. The scores reflect the probability of positive enrichment. (Cut off=2); PeakMValue: The median log2-ratio from probes within the peak region. The score reflects the methylation level of the region; Promoter_Classification: The promoter type decided by the CpG contents in this region; Strand: Strand of the transcript; PeakToTSS: The distance from the centre of the peak to the TSS. (“-” means the peak centre locate in upstream of the TSS); TSS: The start site of the transcript; TTS: The termination site of the transcript. The same below.

Table S2-2 The genes demethylated by HFD

| **Gene**  **Name** | **Peak**  **Start** | **Peak End** | **Peak**  **Length** | **Peak**  **Score** | **PeakMValue** | **Promoter_Classification** | **Strand** | **Peak ToTSS** | **TSS** | **TTS** |
| --- | --- | --- | --- | --- | --- | --- | --- | --- | --- | --- |
| *Kcnq5* | 21951605 | 21951880 | 275 | 2.75 | 2.149 | ICP | - | 280 | 21952023 | 21388483 |
| *Hs6st1* | 36124619 | 36124883 | 264 | 2.45 | 1.258 | HCP | + | -493 | 36125244 | 36163291 |
| *Speg* | 75371802 | 75372327 | 525 | 2.48 | 1.460 | ICP | + | 193 | 75371871 | 75428881 |
| *Aqp12* | 94901831 | 94902073 | 242 | 2.23 | 0.910 | LCP | + | -958 | 94902910 | 94908846 |
| *Gli2* | 120950629 | 120950879 | 250 | 2.24 | 1.078 | HCP | - | -558 | 120950196 | 120730637 |
| *Etnk2* | 135259471 | 135259798 | 327 | 2.08 | 1.313 | ICP | + | -513 | 135260148 | 135276895 |
| *Pogk* | 168339874 | 168340129 | 255 | 2.12 | 1.937 | HCP | - | -42 | 168339959 | 168323741 |
| *Tgfb2* | 188529387 | 188529634 | 247 | 2.19 | 2.436 | LCP | - | 360 | 188529871 | 188447064 |
| *Ado* | 67011216 | 67011468 | 252 | 2.51 | 2.771 | HCP | - | 361 | 67011703 | 67007258 |
| *Tbxa2r* | 80791435 | 80791692 | 257 | 2.39 | 1.653 | LCP | + | 88 | 80791475 | 80797917 |
| *Agap2* | 126515651 | 126515999 | 348 | 2.31 | 1.559 | ICP | + | -137 | 126515962 | 126530225 |
| *Naca* | 127471955 | 127472885 | 930 | 2.25 | 1.402 | ICP | + | 19 | 127472401 | 127485693 |
| *Pex13* | 23565647 | 23565886 | 239 | 2.22 | 1.819 | HCP | - | 168 | 23565935 | 23546478 |
| *Pus10* | 23565647 | 23565886 | 239 | 2.22 | 1.819 | HCP | + | -208 | 23565975 | 23632876 |
| *Hnrnph1* | 50191195 | 50191649 | 454 | 2.2 | 2.337 | HCP | + | 202 | 50191220 | 50200030 |
| *Hes7* | 68933572 | 68934103 | 531 | 2.41 | 1.461 | LCP | + | -116 | 68933954 | 68936761 |
| *Nlgn2* | 69648156 | 69649592 | 1436 | 2.35 | 1.938 | LCP | - | -523 | 69648351 | 69636624 |
| *Stat5a* | 100722014 | 100722276 | 262 | 2.48 | 2.141 | ICP | + | 348 | 100721797 | 100746483 |
| *Rab37* | 115015014 | 115015484 | 470 | 2.49 | 1.698 | ICP | + | -242 | 115015491 | 115023554 |
| *Cyth1* | 118109417 | 118110215 | 798 | 2.42 | 2.150 | HCP | - | 90 | 118109906 | 118025479 |
| *Arhgdia* | 120442443 | 120442823 | 380 | 2.14 | 1.486 | HCP | - | 301 | 120442934 | 120438548 |
| *2010109K11Rik* | 33063612 | 33064055 | 443 | 2.41 | 2.676 | HCP | + | 180 | 33063653 | 33067808 |
| *Ston2* | 93025004 | 93025245 | 241 | 2.22 | 1.839 | LCP | - | -248 | 93024876 | 92871448 |
| *Gpr68* | 102145922 | 102146371 | 449 | 2.95 | 1.927 | ICP | - | 261 | 102146408 | 102114891 |
| *Akt1* | 113911992 | 113912402 | 410 | 2.48 | 2.887 | HCP | - | 290 | 113912487 | 113892031 |
| *Tex22* | 114312036 | 114312279 | 243 | 2.39 | 1.228 | ICP | + | -554 | 114312712 | 114327125 |
| *Hist1h2ag* | 22134856 | 22135107 | 251 | 2.33 | 1.571 | HCP | - | -163 | 22134818 | 22134345 |
| *Hist1h2bj* | 22134856 | 22135107 | 251 | 2.33 | 1.571 | HCP | + | -116 | 22135098 | 22135479 |
| *Sfxn1* | 54167384 | 54167674 | 290 | 2.21 | 2.048 | HCP | + | 316 | 54167213 | 54203714 |
| *Caml* | 55724186 | 55724443 | 257 | 2.27 | 0.808 | HCP | + | -50 | 55724365 | 55733777 |
| *Ccdc66* | 28321155 | 28321401 | 246 | 2.5 | 2.129 | HCP | - | 368 | 28321646 | 28295595 |
| *Mphosph8* | 57287232 | 57287494 | 262 | 2.37 | 2.505 | HCP | + | 279 | 57287084 | 57316266 |
| *Ropn1l* | 31383067 | 31383312 | 245 | 2.05 | 2.164 | HCP | - | 254 | 31383444 | 31370964 |
| *Cyc1* | 76174050 | 76174309 | 259 | 2.1 | 2.395 | HCP | + | 227 | 76173952 | 76176364 |
| *Arfgap3* | 83180217 | 83180463 | 246 | 2.19 | 2.527 | HCP | - | 337 | 83180677 | 83130169 |
| *Samm50* | 84022403 | 84022872 | 469 | 2.33 | 1.688 | ICP | + | -24 | 84022662 | 84044733 |
| *Tubgcp6* | 88954110 | 88954365 | 255 | 2.19 | 1.522 | HCP | - | -657 | 88953580 | 88929527 |

Table S2-2 (continued)

| **Gene**  **Name** | **Peak**  **Start** | **Peak End** | **Peak**  **Length** | **Peak**  **Score** | **PeakMValue** | **Promoter_Classification** | **Strand** | **Peak ToTSS** | **TSS** | **TTS** |
| --- | --- | --- | --- | --- | --- | --- | --- | --- | --- | --- |
| *Mkl2* | 13256514 | 13257030 | 516 | 3.04 | 3.080 | HCP | + | 199 | 13256573 | 13417622 |
| *Bdh1* | 31428334 | 31428881 | 547 | 2.45 | 1.099 | ICP | + | -230 | 31428838 | 31458987 |
| *Dcbld2* | 58408623 | 58409028 | 405 | 2.25 | 2.484 | HCP | + | 178 | 58408647 | 58469858 |
| *Mrpl39* | 84735260 | 84735938 | 678 | 2.43 | 1.082 | HCP | - | -173 | 84735426 | 84718525 |
| *4931408A02Rik* | 90830609 | 90830858 | 249 | 2.03 | 0.522 | HCP | + | -623 | 90831357 | 90904940 |
| *Wiz* | 32524688 | 32525614 | 926 | 2.35 | 0.982 | LCP | - | -389 | 32524762 | 32490994 |
| *Tspo2* | 48590920 | 48591455 | 535 | 2.17 | 1.590 | LCP | - | -361 | 48590826 | 48587759 |
| *Wnt8a* | 34701890 | 34702124 | 234 | 2.32 | 1.096 | LCP | + | 26 | 34701981 | 34707715 |
| *March3* | 57084713 | 57085169 | 456 | 2.56 | 2.088 | HCP | - | 261 | 57085202 | 56921369 |
| *Malt1* | 65590818 | 65591072 | 254 | 2.36 | 1.674 | HCP | + | 295 | 65590650 | 65638446 |
| *Rasgrp2* | 6399494 | 6399752 | 258 | 2.23 | 1.389 | ICP | + | -959 | 6400582 | 6415216 |
| *Best1* | 10075857 | 10076097 | 240 | 2.31 | 1.298 | LCP | - | 146 | 10076123 | 10059661 |
| *Kif11* | 37450941 | 37451185 | 244 | 2.44 | 2.702 | HCP | + | 171 | 37450892 | 37496349 |
| *Abca2* | 25283716 | 25284576 | 860 | 2.35 | 1.606 | HCP | + | -47 | 25284193 | 25304059 |
| *Edf1* | 25413148 | 25413617 | 469 | 2.24 | 1.631 | HCP | + | -36 | 25413419 | 25417602 |
| *Atrn* | 130732022 | 130732273 | 251 | 2.34 | 1.550 | HCP | + | -83 | 130732231 | 130856062 |
| *A730017L22Rik* | 130732022 | 130732273 | 251 | 2.34 | 1.550 | HCP | - | -15 | 130732132 | 130698276 |
| *Cbln4* | 171868891 | 171869393 | 502 | 2.05 | 0.930 | ICP | - | -176 | 171868966 | 171861835 |
| *Lama5* | 179960119 | 179960859 | 740 | 2.31 | 2.344 | ICP | - | 75 | 179960564 | 179911077 |
| *Ddx20* | 105490034 | 105490480 | 446 | 2.33 | 2.124 | HCP | - | 232 | 105490489 | 105481379 |
| *Cyp2u1* | 131005648 | 131005900 | 252 | 2.36 | 2.020 | HCP | - | 371 | 131006145 | 130993408 |
| *Palm2* | 57580631 | 57581169 | 538 | 2.18 | 1.252 | HCP | + | -219 | 57581119 | 57730000 |
| *Prkaa2* | 104782302 | 104782820 | 518 | 2.33 | 1.575 | HCP | - | -58 | 104782503 | 104702254 |
| *Edn2* | 119834182 | 119834424 | 242 | 2.53 | 2.331 | LCP | + | 275 | 119834028 | 119839965 |
| *Lin28a* | 133574995 | 133575399 | 404 | 2.35 | 0.404 | LCP | - | -466 | 133574731 | 133559244 |
| *Sh2d5* | 137806569 | 137806817 | 248 | 2.48 | 1.733 | HCP | + | 368 | 137806325 | 137816883 |
| *Fblim1* | 141161678 | 141162175 | 497 | 2.05 | 1.898 | LCP | - | 40 | 141161967 | 141131976 |
| *Dpp6* | 27375276 | 27375529 | 253 | 2.08 | 0.876 | LCP | + | -335 | 27375738 | 28054040 |
| *Spon2* | 33561016 | 33561470 | 454 | 2.07 | 0.624 | LCP | - | -356 | 33560887 | 33556166 |
| *Prom1* | 44492476 | 44492740 | 264 | 2.4 | 2.327 | LCP | - | 367 | 44492975 | 44384860 |
| *Ywhag* | 136410026 | 136410264 | 238 | 2.27 | 2.020 | HCP | - | 366 | 136410511 | 136384248 |
| *Lrwd1* | 136612266 | 136612570 | 304 | 2.73 | 1.630 | HCP | - | -474 | 136611944 | 136598935 |
| *Cyth3* | 144411209 | 144411464 | 255 | 2.02 | 1.378 | LCP | + | -772 | 144412109 | 144471117 |
| *Klhdc10* | 30351661 | 30351914 | 253 | 2.1 | 1.724 | HCP | + | -120 | 30351908 | 30405174 |
| *Zfp862* | 48454381 | 48454624 | 243 | 2.43 | 1.767 | ICP | + | 165 | 48454337 | 48484831 |
| *Tmem176a* | 48791929 | 48792318 | 389 | 2.47 | 1.697 | LCP | + | 469 | 48791654 | 48795363 |
| *Tmem176b* | 48791929 | 48792318 | 389 | 2.47 | 1.697 | LCP | - | -750 | 48791373 | 48783810 |
| *Zfp773* | 7088968 | 7089707 | 739 | 2.32 | 1.981 | LCP | - | 128 | 7089466 | 7083388 |

Table S2-2 (continued)

| **Gene**  **Name** | **Peak**  **Start** | **Peak End** | **Peak**  **Length** | **Peak**  **Score** | **PeakMValue** | **Promoter_Classification** | **Strand** | **Peak ToTSS** | **TSS** | **TTS** |
| --- | --- | --- | --- | --- | --- | --- | --- | --- | --- | --- |
| *Ccdc9* | 16872084 | 16872363 | 279 | 2.34 | 1.636 | HCP | - | -79 | 16872144 | 16859390 |
| *2210010C17Rik* | 20535596 | 20536255 | 659 | 2.15 | 1.656 | LCP | - | 166 | 20536092 | 20522653 |
| *Tex101* | 25456570 | 25457054 | 484 | 2.36 | 2.222 | ICP | - | 257 | 25457069 | 25453030 |
| *Prr12* | 52308162 | 52308913 | 751 | 2.21 | 1.904 | HCP | - | -286 | 52308251 | 52283076 |
| *Tm2d3* | 72837582 | 72837843 | 261 | 2.46 | 1.261 | HCP | + | -589 | 72838302 | 72846799 |
| *Il4ra* | 132695965 | 132696201 | 236 | 2.66 | 2.456 | HCP | + | 288 | 132695795 | 132722988 |
| *1600016N20Rik* | 148399686 | 148399949 | 263 | 2.37 | 1.421 | LCP | - | 161 | 148399979 | 148395941 |
| *Igf2* | 149844830 | 149845684 | 854 | 2.67 | 1.486 | HCP | - | -548 | 149844709 | 149836672 |
| *Ccnd1* | 152125356 | 152125604 | 248 | 2.56 | 2.072 | ICP | - | 350 | 152125830 | 152115835 |
| *AI316807* | 23586282 | 23587302 | 1020 | 2.34 | 0.981 | HCP | - | 559 | 23587351 | 23573085 |
| *Slc20a2* | 23586282 | 23587302 | 1020 | 2.34 | 0.981 | HCP | + | -379 | 23587171 | 23680088 |
| *Gm4975* | 66430555 | 66430806 | 251 | 2.23 | 1.925 | ICP | - | 286 | 66430967 | 66403490 |
| *Olfr370* | 86064794 | 86065301 | 507 | 2.25 | 2.052 | LCP | + | 56 | 86064991 | 86066087 |
| *Slc9a5* | 107871685 | 107872214 | 529 | 2.13 | 0.575 | HCP | + | -207 | 107872157 | 107893781 |
| *Fhod1* | 107871685 | 107872214 | 529 | 2.13 | 0.575 | HCP | - | -79 | 107871870 | 107853059 |
| *Dpep2* | 108520063 | 108520305 | 242 | 2.22 | 0.927 | LCP | - | 139 | 108520323 | 108508956 |
| *Clec18a* | 113606136 | 113606589 | 453 | 2.41 | 1.806 | LCP | - | -754 | 113605608 | 113593396 |
| *Loxl1* | 58161135 | 58161589 | 454 | 2.02 | 0.811 | LCP | - | -343 | 58161019 | 58135529 |
| *Wdr72* | 73957253 | 73957742 | 489 | 2.41 | 0.876 | LCP | + | -642 | 73958140 | 74131010 |
| *Tmem115* | 107436471 | 107436707 | 236 | 2.37 | 2.117 | HCP | + | 314 | 107436275 | 107440987 |
| *Prkar2a* | 108594613 | 108594859 | 246 | 2.41 | 1.724 | HCP | + | 263 | 108594473 | 108651842 |
| *Rpl14* | 120480570 | 120480818 | 248 | 2.12 | 1.539 | HCP | + | 61 | 120480633 | 120483770 |
| *Dlg3* | 97962857 | 97963095 | 238 | 2.42 | 1.478 | ICP | + | -89 | 97963065 | 98013749 |
| *Fndc3c1* | 103681505 | 103681751 | 246 | 2.5 | 1.416 | HCP | - | -1060 | 103680568 | 103615381 |
| *1700045I19Rik* | 160199258 | 160199515 | 257 | 2.19 | 0.891 | ICP | - | -125 | 160199261 | 160198071 |
